# A Four-miRNA-Based Diagnostic Signature for Rheumatoid Arthritis

**DOI:** 10.1155/2022/6693589

**Published:** 2022-02-22

**Authors:** Xu Jiang, Zhenjie Wei, Chao Wang, Qianqian Wang, Yanzhuo Zhang, Chengai Wu

**Affiliations:** ^1^Department of Orthopaedics, Beijing Jishuitan Hospital, The Fourth Clinical Medical College of Peking University, Beijing 100035, China; ^2^Department of Molecular Orthopaedics, Beijing Research Institute of Traumatology and Orthopaedics, Beijing 100035, China

## Abstract

**Background:**

As a chronic inflammatory disease, rheumatoid arthritis (RA) usually leads to cartilage and bone damage, even disability. Earlier detection and diagnosis are crucial to improve the therapeutic efficacy, and the aim of our study is to identify a potential diagnostic signature for RA.

**Methods:**

We downloaded the GSE124373 dataset from the Gene Expression Omnibus (GEO) database. And differential expression analysis of miRNAs was conducted using the *limma* package of R language. The potential targeted mRNAs of differentially expressed miRNAs were predicted using the MiRTarBase database. The *clusterProfiler* package in R language was used to conduct functional enrichment analysis (GO term and KEGG pathway). Then, based on the key miRNAs screened by stepwise regression analysis, the logistic regression model was built and it was evaluated using a 5-fold cross-validation method.

**Results:**

A total of 19 differentially expressed miRNAs in the blood sample of RA patients compared with that of healthy subjects were identified. Nine optimal miRNAs were screened by using stepwise regression analysis, and four key miRNAs hsa-miR-142-5p, hsa-miR-1184, hsa-miR-1246, and hsa-miR-99b-5p were further optimized. Finally, a logistic regression model was built based on the four key miRNAs, which could reliably separate RA patients from healthy subjects.

**Conclusion:**

Our study established a logistic regression diagnostic model based on four crucial miRNAs, which could separate the sample type reliably.

## 1. Introduction

As a systemic chronic inflammatory disease, rheumatoid arthritis (RA) mainly affects the diarthrodial joint and even results in disability [1, 2]. The incidence of RA increased annually from 0.28% in 2009 to 0.32% in 2012, and RA cases are diagnosed with approximately 28.5 per 100,000 person-years in 2010 [3]. The conventional RA therapy is characterized by the poor bioavailability and high and frequent dosing; thus, many medications cannot accurately act on the target zone and might lead to side effects in some extra-articular tissues [4]. For the RA patients with obvious risk factors like early joint damage and high disease activity, their prognoses may benefit from getting earlier diagnoses and timely treatments [1]. Therefore, identification of specific and potential diagnostic biomarkers for RA contributes to improve the therapeutic efficacy and is more urgent.

MicroRNAs (miRNAs) are a class of noncoding RNAs with 20-22 nucleotides in length in eukaryocyte [5]. More and more miRNAs have been identified to regulate a large number of gene expression involved in various biological processes like cell proliferation, invasion, migration, and epithelial-mesenchymal transition (EMT) in multiple human cancers [6–8]. Meanwhile, the abnormal expression of many miRNAs in human diseases is always observed, some of which are found to be potential diagnostic biomarkers. For example, Liu et al. found that miR-940 was significantly downregulated in breast cancer patients compared with the normal samples, suggesting that serum downregulated miR-940 might be a reliable diagnostic biomarker in breast cancer patients [9]. miRNA-122/222 levels have been reported to be a potential diagnostic biomarker in Egyptian patients with chronic hepatitis C [10]. Kong et al. revealed that miR-142-5p in colon cancer specimens is lower than that in adjacent samples and indicated that miR-142-5p might be a possible diagnostic biomarker for colon cancer [11]. In addition, there were also several miRNAs predicted to be potential diagnostic biomarkers for RA such as miR-146a, miR-499 [12], miR-5196 [13], and miR-146a [14]. However, the research associated with a diagnostic model based on multiple key miRNAs in RA has not been well explored and attracted us to focus on it.

In our present study, the expression profile of patients with and without RA was obtained from the GEO database. Our study identified 19 miRNAs which exhibited significant difference between RA patients and normal samples. Furthermore, four key miRNAs (hsa-miR-142-5p, hsa-miR-1184, hsa-miR-1246, and hsa-miR-99b-5p) that might be closely involved in RA development were screened. Finally, the logistic regression diagnostic model was successfully established based on the four crucial miRNAs and proved that it could separate RA patients from healthy subjects.

## 2. Materials and Methods

### 2.1. Data Collection

The mRNA expression profile (Number: GSE124373) consists of 28 RA blood samples and 18 healthy specimens (Table S1), which was obtained from the Gene Expression Omnibus database (GEO) database (https://www.ncbi.nlm.nih.gov/geo/). The expression analysis of mRNA profile was detected by Affymetrix Multispecies miRNA-4 Array.

The clinical samples used for subsequent validation were collected from the Beijing Jishuitan Hospital. A total of 8 RA and 5 control blood samples were obtained. The informed consents were obtained from all participants. Our study was approved by the local ethics committee, in accordance with the Helsinki Declaration.

### 2.2. Differential Expression Analyses

To further analyze the differentially expressed genes, we removed the genes with missing value, and standardization was conducted according to the robust multiarray (RMA) method. Then, the *limma* function package in R language [15] was used to perform the differential expression analysis of miRNAs, with ∣log2(fold change (FC)) | >1 and FDR (false discovery rate) < 0.05 as the significant threshold.

### 2.3. The Prediction of Potential Targets of miRNAs

The potential targets of differentially expressed miRNAs were predicted using MiRTarBase (Release 7.0: Sept. 15, 2017 http://mirtarbase.mbc.nctu.edu.tw). Then, these potential targeted mRNAs were applied for the functional enrichment analysis.

### 2.4. Functional Enrichment Analysis

The Gene Ontology (GO) and Kyoto Encyclopedia of Genes and Genomes (KEGG) pathway enrichment analyses were conducted using *clusterProfiler* function package in R language [16]. And terms with *P* < 0.05 were considered as significantly enriched ones.

### 2.5. Logistic Regression Model Construction

Logistic regression is a widely used method for classification according to a set of variables. Here, the expression values of miRNAs were used as variables to predict the sample type (RA or not). Then, stepwise regression analysis was used to further screen the optimal miRNAs based on their *P* values, and the variables with *P* < 0.05 were selected to construct the final logistic regression model.

To determine the accuracy of the final model, a 5-fold cross-validation method was conducted. Briefly, all the samples in GSE124373 were randomly divided into 5 groups, of which 4 groups of samples were regarded as the training sets to construct a logistic model and another group of sample was considered as the verification set to explore the reliability of this model. This process was repeated for 5 times in total. The cross-validation process can ensure that each subsample is trained and tested, which can reduce the error and provide a more realistic detection capability of the model.

### 2.6. qRT-PCR

Total RNA was extracted from the blood samples using Trizol reagent (Tiangen Biochemical, China), which was then reverse transcribed to cDNA utilizing FastQuant RT Kit containing gDNase (Tiangen Biochemical, China). The PCR amplification was conducted on ABI7500 real-time fluorescent quantitative PCR instrument, with Power SYBR® Green PCR Kit (Applied Biosystems). The primer sequences are shown in Table 1. The following procedure was carried out: predenaturation 95°C for 10 min, 95°C for 15 sec, and 60°C for 60 sec, 40 cycles. Three repeats were adopted. And the relative expression level was calculated using the 2^-*ΔΔ*CT^ method.

## 3. Results

### 3.1. A Total of 19 Differentially Expressed miRNAs in GSE124373 Were Identified

**Figure 1:**
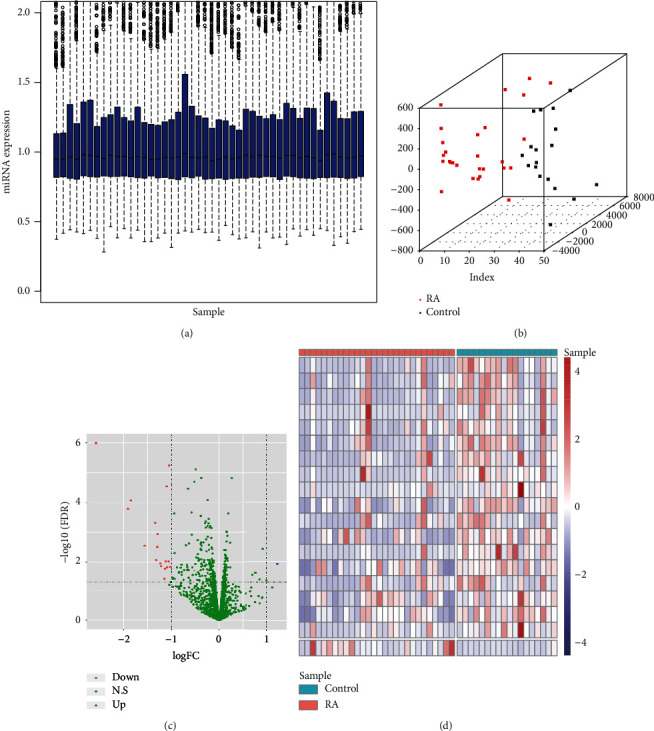
The results of differentially expressed miRNAs. (a) The boxplot of expression of miRNAs after standardization in the GSE124373 dataset. Horizontal axis: sample; vertical axis: relative expression of miRNAs. (b) The PCA results of miRNAs in the GSE124373 dataset. Red: rheumatoid arthritis (RA); black: healthy subjects. The closer the distance, the more similar the miRNAs. (c) The volcano plot of differentially expressed miRNAs between RA samples vs. healthy samples. Horizontal axis: log2FC; vertical axis: -log10(FDR). Red: downregulated miRNAs; blue: upregulated miRNAs; green: nonsignificance. (d) The heat map of differentially expressed miRNAs between RA samples vs. healthy samples. Horizontal axis: differentially expressed miRNAs; vertical axis: samples. Red: high expression; blue: low expression.

To remove the batch effects, all data in GSE124373 was standardized based on the RMA method, and we found that there was no obvious deviation in each sample (Figure 1(a)), indicating that the data could be used for the following analysis. To further confirm the repeatability of the data, we have performed the principle component analysis (PCA) on all miRNAs, and the results showed that the RA group and control group were efficiently separated (Figure 1(b)), which indicated a relatively high reproducibility. Subsequently, differential expression analysis was performed based on the expression values of miRNAs in all samples, and the analysis identified 19 differentially expressed miRNAs (1 upregulated miRNA and 18 downregulated miRNAs) in RA specimens compared with healthy samples (Figure 1(c)). Moreover, there were significant differences in the expression values of 19 differentially expressed miRNAs between two groups (Figure 1(d)). The results above suggested that these 19 differentially expressed miRNAs might be involved in the progression of RA.

### 3.2. Functional Enrichment Analysis

**Figure 2:**
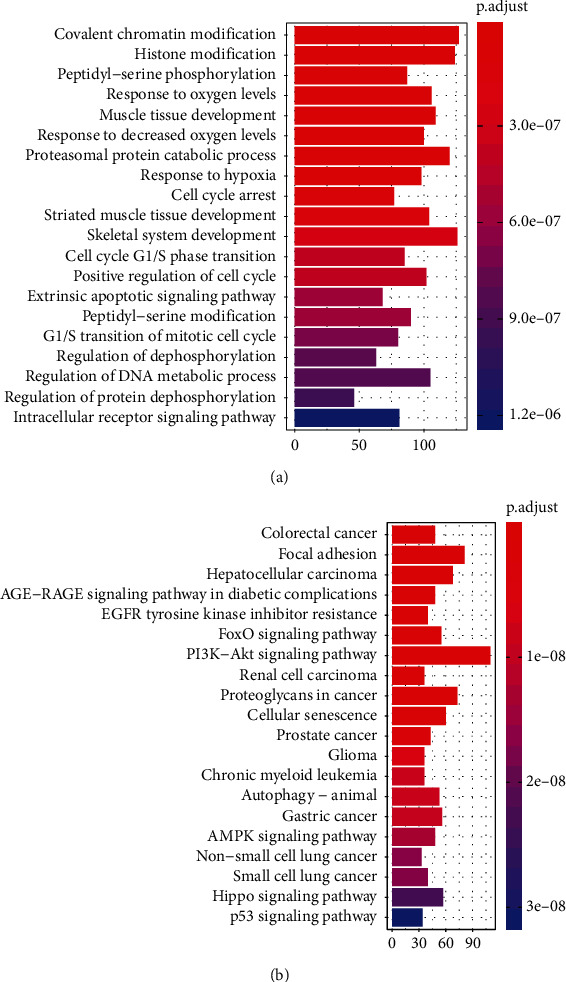
The functional enrichment results based on 2899 targeted mRNAs. (a) The top 20 significantly enriched GO terms. Horizontal axis: number of mRNAs; vertical axis: title of GO terms. (b) The top 20 significantly enriched KEGG pathways. Horizontal axis: number of mRNAs; vertical axis: title of KEGG pathways.

To investigate the metabolic pathways closely associated with the occurrence or development of RA, the potential target mRNAs of the 19 differentially expressed miRNAs were predicted by using the miRTarBase database, which obtained 2899 potential targeted mRNAs in total. Then, functional enrichment analysis was performed on the 2899 mRNAs and there were 1325 significantly enriched GO terms and 113 significantly enriched KEGG pathways (*P* adjust < 0.05). The top 20 significantly enriched GO terms are shown in Figure 2(a), and the top 20 significantly enriched KEGG pathways are shown in Figure 2(b). The detailed GO and KEGG pathway enriched results are shown in Table S2. These significantly enriched GO terms and KEGG pathways were probably related to the progression of RA.

### 3.3. The Logistic Regression Model Could Reliably Separate RA Samples from Healthy Samples

**Figure 3:**
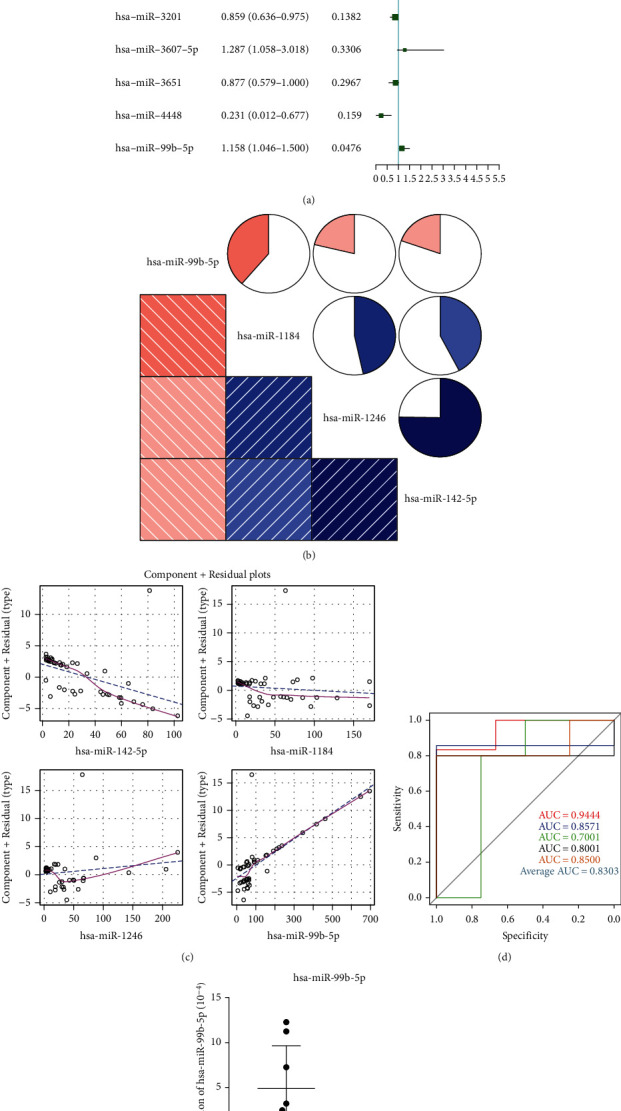
The construction of the logistic regression diagnostic model. (a) Nine miRNAs screened based on the stepwise regression analysis. *P* < 0.05 suggested that the miRNA contributed more to the model. (b) The correlation heat map of four key miRNAs in the model. The deeper the red and blue, the greater the correlation between them. (c) The component plus residual plot of 4 key miRNAs in the model. The obvious linear relationship between the horizontal and vertical axis implies that the independent variables are suitable to be brought in the model. (d) The ROC curve of the logistic regression diagnostic model. The AUC (area under the curve) value can intuitively evaluate the quality of the model; the larger the AUC value from 0 to 1, the better the model. (e) Higher hsa-miR-99b-5p expression was observed in RA samples compared with controls.

Firstly, the logistic regression model 1 was constructed based on the 19 differentially expressed miRNAs. Subsequently, we have conducted the stepwise regression analysis on these miRNAs in order to build the model based on fewer miRNAs with stronger interpretation, and 9 miRNAs were further identified. Then, the logistic regression model was constructed again by bringing in these 9 miRNAs as variables and found that the *P* value of 4 miRNAs (hsa-miR-142-5p, hsa-miR-1184, hsa-miR-1246, and hsa-miR-99b-5p) was less than 0.05 (Figure 3(a)), implying that these 4 miRNAs contributed more to the logistic regression model and might be closely associated with the development of RA. Meanwhile, based on a correlation coefficient greater than 0.8, there was no strong correlation among these four miRNAs (Figure 3(b) and Table S3), suggesting that these four miRNAs did not influence the accuracy of our logistic regression model. Subsequently, these four miRNAs were used to reconstruct the final logistic regression model, the results of which showed that the final model obeyed the normal distribution (Fig. S1A). The independent variables included in the model had a good linear relationship with the response variables (Figure 3(c)), and there were no extreme points that significantly affected the accuracy of the model (Fig. S1B). Finally, the 5-fold cross-validation method was applied to determine the reliability of the model. The AUC values of the 5 logistic models in the 5 validation sets were 0.9444, 0.8571, 0.7001, 0.8001, and 0.8500, respectively, with the average AUC 0.8303 (Figure 3(d)). The results indicated that the logistic regression model constructed based on four key miRNAs was able to reliably separate RA samples from healthy samples.

Among these 4 crucial miRNAs, hsa-miR-142-5p, hsa-miR-1184, and hsa-miR-1246 were downregulated in RA samples, while hsa-miR-99b-5p showed an opposite tendency in RA samples. Thus, we verified hsa-miR-99b-5p's expression in clinical samples. Our results indicated that there was higher hsa-miR-99b-5p expression in RA samples compared with controls (Figure 3(e)), which was consistent with our bioinformatic analysis.

## 4. Discussion

Due to the poor therapeutic efficacy for RA at present, the early diagnosis is helpful for the treatment and prognosis of RA [4]. The current diagnostic methods for RA are mainly based on clinical manifestations and imaging examination, and some atypical cases are easy to be missed or misdiagnosed [17, 18]. In the present study, basing on the publicly obtained mRNA expression profile of blood samples from RA and healthy subjects, we have analyzed the differentially expressed miRNAs, as well as the potential diagnostic values of miRNAs.

Increasing evidence demonstrates that RA is an autoimmune disease, which is often accompanied by chronic and aggressive polyarthritis [19]. The occurrence of RA results from various genetic and complex environmental factors, which lead to the immune perturbation in the innate and adaptive immune system, of which chronic inflammation is one of the most notable effects [20]. Recent studies have linked RA with a network of cytokines, and these multifunctional proteins can stimulate specific immune responses [21]. For example, in RA experimental models, IL-1 blockers like IL-1 receptor antagonist (IL-1Ra) can markedly attenuate clinical and histological disease parameters [22]. IL-1*β* is usually generated by the macrophages belonging to the synovial lining, which is demonstrated to contribute for the joint inflammation and damage in RA [23]. In addition, innate immune sensors, such as Toll-like receptors (TLRs) and nucleotide-binding oligomerization domain-like receptors (NLRs), are evidenced to be closely involved in the innate inflammatory induction and adaptive immune responses in RA [24]. These studies all reveal that immune response plays crucial roles in the development and progression of RA. In our study, we identified that 19 differentially expressed miRNAs in the blood samples of RA compared with healthy subjects and 2899 potential target mRNAs were predicted. Moreover, GO term and KEGG pathway enrichment analyses were performed based on 2899 predictive mRNAs and found that several pathways including EGFR tyrosine kinase inhibitor resistance, PI3K-AKT signaling pathway, and FOXO signaling pathway were significantly enriched when RA occurred. These results suggested that our analysis confirmed the previous study in RA, which provided the reliable theoretical basis for the subsequent model construction.

To build a more explanatory model, stepwise regression analysis was conducted and four key miRNAs (hsa-miR-142-5p, hsa-miR-1184, hsa-miR-1246, and hsa-miR-99b-5p) were screened, which contributed more to the model. Krissansen et al. revealed that miR-1246 was significantly upregulated in the sera of RA patients compared with healthy subjects, which indicated that miR-1246 could indirectly activate the proinflammatory nuclear factor of activated T cells [25]. Another recent study has documented the role of miR-99b-5p in erosion progression of RA patients, and significantly higher miR-99b-5p was observed in early RA patients with erosions [26]. miR-142-5p has been identified to regulate CD4^+^ T cells through PD-L1 expression via regulating the PTEN pathway in non-small-cell lung cancer [27]. In addition, Grenda et al. demonstrated that there was a positive correlation between PD-L1-mRNA and miR-1184 in non-small-cell lung cancer [28]. All the above evidence suggested that these 4 miRNAs probably participate in the immune response in the progression of RA and could be potentially considered as biomarkers. Subsequently, a logistic regression diagnostic model was constructed based on these four miRNAs, and the 5-fold cross-validation method was applied to determine the reliability of this model. The results of validation demonstrated that this diagnostic model could reliably separate RA patients from healthy subjects.

Although our results revealed the potential diagnostic values of these four miRNAs in RA, their actual functions needed to be further studied in the future. Moreover, though our diagnostic model was successfully validated in clinical samples, the validation in a larger sample size in the near future should be done to improve the reliability of our diagnostic model.

## 5. Conclusions

To summarize, our study has established a relatively reliable diagnostic model by bringing in four key miRNAs (hsa-miR-142-5p, hsa-miR-1184, hsa-miR-1246, and hsa-miR-99b-5p), which might contribute to separate RA samples from healthy subjects. Our findings are expected to give more alternatives for the clinical management of RA.

## Figures and Tables

**Table 1:** miRNA primer sequences for RT-PCR.

Genes	RT primer (5′-3′)	Forward primer (5′-3′)
hsa-miR-99b-5p	GTCGTATCCAGTGCAGGGTCCGAGGTATTCGCACTGGATACGACCGCAAG	CACCCGTAGAACCGACC
hsa-miR-16-5p	GTCGTATCCAGTGCAGGGTCCGAGGTATTCGCACTGGATACGACCGCCAA	CATAGCAGCACGTAAATATTGGC

Universe-R: GTGCAGGGTCCGAGGT.

## Data Availability

The mRNA expression profile (Number: GSE124373) that consists of 28 blood samples from RA patients and 18 blood samples from healthy subjects was downloaded from the Gene Expression Omnibus database (GEO, https://www.ncbi.nlm.nih.gov/geo/).

## Additional Points

*Highlight*. (i) A total of 19 differentially expressed miRNAs between patients with and without rheumatoid arthritis were identified. (ii) Four miRNAs, hsa-miR-142-5p, hsa-miR-1184, hsa-miR-1246, and hsa-miR-99b-5p, appear to be involved in the development of rheumatoid arthritis.(iii) The logistic regression model constructed based on the four key miRNAs could reliably separate rheumatoid arthritis patients from healthy subjects.

## Supplementary Materials

Supplementary 1 Table S1: detailed sample information.

Supplementary 2 Table S2: the full list of significantly enriched GO terms (BP terms, CC terms, and MF terms) and KEGG pathways.

Supplementary 3 Table S3: the correlation analysis results of four key miRNAs.

Supplementary 4 Fig. S1: the diagnosis diagram of the logistic regression model. (A) The normal Q-Q graph. The points on the graph should fall on a line at an angle of 45 degrees. If the deviation is too large, then the model violates the normal assumption. (B) The diagram of residuals vs. leverage. The red dotted line indicates the COOK distance. Generally, a point with the COOK greater than 0.5 is a very “influential” point, which affects the reliability of the model.
