# Supplementary material for: A Four-miRNA-Based Diagnostic Signature for Rheumatoid Arthritis
Source: Dis Markers. 2022 Feb 22;2022:6693589. doi: 10.1155/2022/6693589 (PMC8889404; doi:10.1155/2022/6693589)
Supplement: Supplementary 1 — Table S1: detailed sample information. [file 6693589.f1.pdf]

Table S1. Detailed sample information.

!Sample_g	!Sample_title	!Sample_status	!Sample_submission_date
GSM3530	RNA from RA pateint P01	Public on Mar 17	Dec 26 2018
GSM3530	RNA from RA pateint P02	Public on Mar 17	Dec 26 2018
GSM3530	RNA from RA pateint P03	Public on Mar 17	Dec 26 2018
GSM3530	RNA from RA pateint P04	Public on Mar 17	Dec 26 2018
GSM3530	RNA from RA pateint P05	Public on Mar 17	Dec 26 2018
GSM3530	RNA from RA pateint P06	Public on Mar 17	Dec 26 2018
GSM3530	RNA from RA pateint P07	Public on Mar 17	Dec 26 2018
GSM3530	RNA from RA pateint P08	Public on Mar 17	Dec 26 2018
GSM3530	RNA from RA pateint P09	Public on Mar 17	Dec 26 2018
GSM3530	RNA from RA pateint P10	Public on Mar 17	Dec 26 2018
GSM3530	RNA from RA pateint P11	Public on Mar 17	Dec 26 2018
GSM3530	RNA from RA pateint P12	Public on Mar 17	Dec 26 2018
GSM3530	RNA from RA pateint P13	Public on Mar 17	Dec 26 2018
GSM3530	RNA from RA pateint P14	Public on Mar 17	Dec 26 2018
GSM3530	RNA from RA pateint P15	Public on Mar 17	Dec 26 2018
GSM3530	RNA from RA pateint P16	Public on Mar 17	Dec 26 2018
GSM3530	RNA from RA pateint P17	Public on Mar 17	Dec 26 2018
GSM3530	RNA from RA pateint P18	Public on Mar 17	Dec 26 2018
GSM3530	RNA from RA pateint P19	Public on Mar 17	Dec 26 2018
GSM3530	RNA from RA pateint P20	Public on Mar 17	Dec 26 2018
GSM3530	RNA from RA pateint P21	Public on Mar 17	Dec 26 2018
GSM3530	RNA from RA pateint P22	Public on Mar 17	Dec 26 2018
GSM3530	RNA from RA pateint P23	Public on Mar 17	Dec 26 2018
GSM3530	RNA from RA pateint P24	Public on Mar 17	Dec 26 2018
GSM3530	RNA from RA pateint P25	Public on Mar 17	Dec 26 2018
GSM3530	RNA from RA pateint P26	Public on Mar 17	Dec 26 2018
GSM3530	RNA from RA pateint P27	Public on Mar 17	Dec 26 2018
GSM3530	RNA from RA pateint P28	Public on Mar 17	Dec 26 2018
GSM3530	RNA from healthy control H01	Public on Mar 17	Dec 26 2018
GSM3530	RNA from healthy control H02	Public on Mar 17	Dec 26 2018
GSM3530	RNA from healthy control H03	Public on Mar 17	Dec 26 2018
GSM3530	RNA from healthy control H04	Public on Mar 17	Dec 26 2018
GSM3530	RNA from healthy control H05	Public on Mar 17	Dec 26 2018
GSM3530	RNA from healthy control H06	Public on Mar 17	Dec 26 2018
GSM3530	RNA from healthy control H07	Public on Mar 17	Dec 26 2018
GSM3530	RNA from healthy control H08	Public on Mar 17	Dec 26 2018
GSM3530	RNA from healthy control H09	Public on Mar 17	Dec 26 2018
GSM3530	RNA from healthy control H10	Public on Mar 17	Dec 26 2018
GSM3530	RNA from healthy control H11	Public on Mar 17	Dec 26 2018
GSM3530	RNA from healthy control H12	Public on Mar 17	Dec 26 2018
GSM3530	RNA from healthy control H13	Public on Mar 17	Dec 26 2018
GSM3530	RNA from healthy control H14	Public on Mar 17	Dec 26 2018
GSM3530	RNA from healthy control H15	Public on Mar 17	Dec 26 2018
GSM3530	RNA from healthy control H16	Public on Mar 17	Dec 26 2018
GSM3530	RNA from healthy control H17	Public on Mar 17	Dec 26 2018
GSM3530	RNA from healthy control H18	Public on Mar 17	Dec 26 2018

!Sample_last_update_date	!Sample_o	!Sample_characteristi	!Sample_c	!Sample_t	!Sample_g	!Sample_n
Mar 17 2020	Homo sapi	gender: female	tissue: peri	Trizol reag	15-ml peri	total RNA
Mar 17 2020	Homo sapi	gender: female	tissue: peri	Trizol reag	15-ml peri	total RNA
Mar 17 2020	Homo sapi	gender: female	tissue: peri	Trizol reag	15-ml peri	total RNA
Mar 17 2020	Homo sapi	gender: female	tissue: peri	Trizol reag	15-ml peri	total RNA
Mar 17 2020	Homo sapi	gender: female	tissue: peri	Trizol reag	15-ml peri	total RNA
Mar 17 2020	Homo sapi	gender: female	tissue: peri	Trizol reag	15-ml peri	total RNA
Mar 17 2020	Homo sapi	gender: female	tissue: peri	Trizol reag	15-ml peri	total RNA
Mar 17 2020	Homo sapi	gender: female	tissue: peri	Trizol reag	15-ml peri	total RNA
Mar 17 2020	Homo sapi	gender: female	tissue: peri	Trizol reag	15-ml peri	total RNA
Mar 17 2020	Homo sapi	gender: female	tissue: peri	Trizol reag	15-ml peri	total RNA
Mar 17 2020	Homo sapi	gender: female	tissue: peri	Trizol reag	15-ml peri	total RNA
Mar 17 2020	Homo sapi	gender: female	tissue: peri	Trizol reag	15-ml peri	total RNA
Mar 17 2020	Homo sapi	gender: female	tissue: peri	Trizol reag	15-ml peri	total RNA
Mar 17 2020	Homo sapi	gender: female	tissue: peri	Trizol reag	15-ml peri	total RNA
Mar 17 2020	Homo sapi	gender: female	tissue: peri	Trizol reag	15-ml peri	total RNA
Mar 17 2020	Homo sapi	gender: female	tissue: peri	Trizol reag	15-ml peri	total RNA
Mar 17 2020	Homo sapi	gender: female	tissue: peri	Trizol reag	15-ml peri	total RNA
Mar 17 2020	Homo sapi	gender: female	tissue: peri	Trizol reag	15-ml peri	total RNA
Mar 17 2020	Homo sapi	gender: female	tissue: peri	Trizol reag	15-ml peri	total RNA
Mar 17 2020	Homo sapi	gender: female	tissue: peri	Trizol reag	15-ml peri	total RNA
Mar 17 2020	Homo sapi	gender: female	tissue: peri	Trizol reag	15-ml peri	total RNA
Mar 17 2020	Homo sapi	gender: female	tissue: peri	Trizol reag	15-ml peri	total RNA
Mar 17 2020	Homo sapi	gender: female	tissue: peri	Trizol reag	15-ml peri	total RNA
Mar 17 2020	Homo sapi	gender: female	tissue: peri	Trizol reag	15-ml peri	total RNA
Mar 17 2020	Homo sapi	gender: female	tissue: peri	Trizol reag	15-ml peri	total RNA
Mar 17 2020	Homo sapi	gender: female	tissue: peri	Trizol reag	15-ml peri	total RNA
Mar 17 2020	Homo sapi	gender: female	tissue: peri	Trizol reag	15-ml peri	total RNA
Mar 17 2020	Homo sapi	gender: female	tissue: peri	Trizol reag	15-ml peri	total RNA
Mar 17 2020	Homo sapi	gender: female	tissue: peri	Trizol reag	15-ml peri	total RNA
Mar 17 2020	Homo sapi	gender: female	tissue: peri	Trizol reag	15-ml peri	total RNA
Mar 17 2020	Homo sapi	gender: female	tissue: peri	Trizol reag	15-ml peri	total RNA
Mar 17 2020	Homo sapi	gender: female	tissue: peri	Trizol reag	15-ml peri	total RNA
Mar 17 2020	Homo sapi	gender: female	tissue: peri	Trizol reag	15-ml peri	total RNA
Mar 17 2020	Homo sapi	gender: female	tissue: peri	Trizol reag	15-ml peri	total RNA
Mar 17 2020	Homo sapi	gender: female	tissue: peri	Trizol reag	15-ml peri	total RNA
Mar 17 2020	Homo sapi	gender: female	tissue: peri	Trizol reag	15-ml peri	total RNA
Mar 17 2020	Homo sapi	gender: female	tissue: peri	Trizol reag	15-ml peri	total RNA
Mar 17 2020	Homo sapi	gender: female	tissue: peri	Trizol reag	15-ml peri	total RNA
Mar 17 2020	Homo sapi	gender: female	tissue: peri	Trizol reag	15-ml peri	total RNA
Mar 17 2020	Homo sapi	gender: female	tissue: peri	Trizol reag	15-ml peri	total RNA
Mar 17 2020	Homo sapi	gender: female	tissue: peri	Trizol reag	15-ml peri	total RNA
Mar 17 2020	Homo sapi	gender: female	tissue: peri	Trizol reag	15-ml peri	total RNA
Mar 17 2020	Homo sapi	gender: female	tissue: peri	Trizol reag	15-ml peri	total RNA
Mar 17 2020	Homo sapi	gender: female	tissue: peri	Trizol reag	15-ml peri	total RNA
Mar 17 2020	Homo sapi	gender: female	tissue: peri	Trizol reag	15-ml peri	total RNA
Mar 17 2020	Homo sapi	gender: female	tissue: peri	Trizol reag	15-ml peri	total RNA

!Sample_e	!Sample_c	!Sample_contact_country
Total RNA	Suzhou	China
Total RNA	Suzhou	China
Total RNA	Suzhou	China
Total RNA	Suzhou	China
Total RNA	Suzhou	China
Total RNA	Suzhou	China
Total RNA	Suzhou	China
Total RNA	Suzhou	China
Total RNA	Suzhou	China
Total RNA	Suzhou	China
Total RNA	Suzhou	China
Total RNA	Suzhou	China
Total RNA	Suzhou	China
Total RNA	Suzhou	China
Total RNA	Suzhou	China
Total RNA	Suzhou	China
Total RNA	Suzhou	China
Total RNA	Suzhou	China
Total RNA	Suzhou	China
Total RNA	Suzhou	China
Total RNA	Suzhou	China
Total RNA	Suzhou	China
Total RNA	Suzhou	China
Total RNA	Suzhou	China
Total RNA	Suzhou	China
Total RNA	Suzhou	China
Total RNA	Suzhou	China
Total RNA	Suzhou	China
Total RNA	Suzhou	China
Total RNA	Suzhou	China
Total RNA	Suzhou	China
Total RNA	Suzhou	China
Total RNA	Suzhou	China
Total RNA	Suzhou	China
Total RNA	Suzhou	China
Total RNA	Suzhou	China
Total RNA	Suzhou	China
Total RNA	Suzhou	China
Total RNA	Suzhou	China
Total RNA	Suzhou	China
Total RNA	Suzhou	China
Total RNA	Suzhou	China
Total RNA	Suzhou	China
Total RNA	Suzhou	China
Total RNA	Suzhou	China
Total RNA	Suzhou	China
