# Supplementary material for: A Four-miRNA-Based Diagnostic Signature for Rheumatoid Arthritis
Source: Dis Markers. 2022 Feb 22;2022:6693589. doi: 10.1155/2022/6693589 (PMC8889404; doi:10.1155/2022/6693589)
Supplement: Supplementary 2 — Table S2: the full list of significantly enriched GO terms (BP terms, CC terms, and MF terms) and KEGG pathways. [file 6693589.f2.pdf]

	ONTOLOGY	ID	Descriptio	GeneRatio	BgRatio	pvalue	p.adjust
GO:0016569	BP	GO:0016569	covalent c	127/2653	464/17653	2.91E-12	9.68E-09
GO:0016570	BP	GO:0016570	histone mo	124/2653	450/17653	3.21E-12	9.68E-09
GO:0018105	BP	GO:0018105	peptidyl-s	87/2653	288/17653	3.50E-11	5.98E-08
GO:0070482	BP	GO:0070482	response t	106/2653	378/17653	3.97E-11	5.98E-08
GO:0060537	BP	GO:0060537	muscle tis	109/2653	396/17653	7.17E-11	8.65E-08
GO:0036293	BP	GO:0036293	response t	100/2653	354/17653	9.02E-11	8.92E-08
GO:0010498	BP	GO:0010498	proteasoma	120/2653	453/17653	1.15E-10	8.92E-08
GO:0001666	BP	GO:0001666	response t	98/2653	346/17653	1.18E-10	8.92E-08
GO:0007050	BP	GO:0007050	cell cycle	77/2653	250/17653	1.69E-10	1.13E-07
GO:0014706	BP	GO:0014706	striated m	104/2653	379/17653	2.37E-10	1.43E-07
GO:0001501	BP	GO:0001501	skeletal s	126/2653	493/17653	4.79E-10	2.63E-07
GO:0044843	BP	GO:0044843	cell cycle	85/2653	295/17653	8.04E-10	3.83E-07
GO:0045787	BP	GO:0045787	positive r	102/2653	377/17653	8.27E-10	3.83E-07
GO:0097191	BP	GO:0097191	extrinsic	68/2653	219/17653	1.34E-09	5.66E-07
GO:0018209	BP	GO:0018209	peptidyl-s	90/2653	322/17653	1.41E-09	5.66E-07
GO:0000082	BP	GO:0000082	G1/S trans	80/2653	276/17653	1.86E-09	7.00E-07
GO:0035303	BP	GO:0035303	regulation	63/2653	199/17653	2.28E-09	8.08E-07
GO:0051052	BP	GO:0051052	regulation	105/2653	399/17653	2.48E-09	8.32E-07
GO:0035304	BP	GO:0035304	regulation	46/2653	127/17653	2.95E-09	9.38E-07
GO:0030522	BP	GO:0030522	intracellu	81/2653	285/17653	4.02E-09	1.21E-06
GO:0048193	BP	GO:0048193	Golgi vesi	96/2653	360/17653	5.80E-09	1.66E-06
GO:0048545	BP	GO:0048545	response t	102/2653	391/17653	6.91E-09	1.89E-06
GO:0034330	BP	GO:0034330	cell junct	77/2653	270/17653	8.21E-09	2.15E-06
GO:0043161	BP	GO:0043161	proteasome	104/2653	403/17653	9.15E-09	2.22E-06
GO:0009896	BP	GO:0009896	positive r	102/2653	393/17653	9.22E-09	2.22E-06
GO:0048732	BP	GO:0048732	gland deve	109/2653	430/17653	1.16E-08	2.62E-06
GO:0031331	BP	GO:0031331	positive r	90/2653	335/17653	1.17E-08	2.62E-06
GO:0010810	BP	GO:0010810	regulation	59/2653	191/17653	1.99E-08	4.23E-06
GO:0043200	BP	GO:0043200	response t	40/2653	109/17653	2.04E-08	4.23E-06
GO:0071230	BP	GO:0071230	cellular r	28/2653	63/17653	2.23E-08	4.47E-06
GO:0048660	BP	GO:0048660	regulation	48/2653	143/17653	2.30E-08	4.48E-06
GO:0051054	BP	GO:0051054	positive r	65/2653	220/17653	2.68E-08	4.87E-06
GO:0031647	BP	GO:0031647	regulation	72/2653	253/17653	2.73E-08	4.87E-06
GO:0001655	BP	GO:0001655	urogenital	85/2653	316/17653	2.79E-08	4.87E-06
GO:0033002	BP	GO:0033002	muscle cel	63/2653	211/17653	2.83E-08	4.87E-06
GO:0098727	BP	GO:0098727	maintenanc	51/2653	157/17653	2.91E-08	4.87E-06
GO:0071375	BP	GO:0071375	cellular r	84/2653	312/17653	3.22E-08	4.97E-06
GO:0018107	BP	GO:0018107	peptidyl-t	42/2653	119/17653	3.37E-08	4.97E-06
GO:0061458	BP	GO:0061458	reproducti	108/2653	433/17653	3.39E-08	4.97E-06
GO:0010921	BP	GO:0010921	regulation	54/2653	171/17653	3.42E-08	4.97E-06
GO:0001837	BP	GO:0001837	epithelial	46/2653	136/17653	3.45E-08	4.97E-06
GO:0003170	BP	GO:0003170	heart valv	21/2653	40/17653	3.46E-08	4.97E-06
GO:0006470	BP	GO:0006470	protein de	82/2653	303/17653	3.66E-08	5.13E-06
GO:2001233	BP	GO:2001233	regulation	98/2653	383/17653	3.85E-08	5.27E-06
GO:0048608	BP	GO:0048608	reproducti	107/2653	430/17653	4.47E-08	5.99E-06
GO:0048659	BP	GO:0048659	smooth mus	48/2653	146/17653	4.82E-08	6.32E-06
GO:0071229	BP	GO:0071229	cellular r	60/2653	200/17653	4.96E-08	6.37E-06
GO:0090068	BP	GO:0090068	positive r	76/2653	277/17653	6.16E-08	7.74E-06
GO:0043666	BP	GO:0043666	regulation	39/2653	109/17653	6.75E-08	8.31E-06
GO:0031589	BP	GO:0031589	cell-subst	84/2653	319/17653	9.36E-08	1.13E-05

GO:0031056	BP	GO:0031056	regulation	45/2653	136/17653	1.01E-07	1.19E-05
GO:0097193	BP	GO:0097193	intrinsic	78/2653	290/17653	1.03E-07	1.19E-05
GO:0035196	BP	GO:0035196	production	21/2653	42/17653	1.05E-07	1.19E-05
GO:0043434	BP	GO:0043434	response t	105/2653	427/17653	1.13E-07	1.26E-05
GO:0006260	BP	GO:0006260	DNA replic	79/2653	296/17653	1.22E-07	1.33E-05
GO:0071559	BP	GO:0071559	response t	66/2653	233/17653	1.24E-07	1.33E-05
GO:0045216	BP	GO:0045216	cell-cell	67/2653	238/17653	1.29E-07	1.36E-05
GO:0019827	BP	GO:0019827	stem cell	49/2653	155/17653	1.40E-07	1.40E-05
GO:0003179	BP	GO:0003179	heart valv	19/2653	36/17653	1.40E-07	1.40E-05
GO:0071560	BP	GO:0071560	cellular r	65/2653	229/17653	1.41E-07	1.40E-05
GO:0031050	BP	GO:0031050	dsRNA frag	22/2653	46/17653	1.44E-07	1.40E-05
GO:0070918	BP	GO:0070918	production	22/2653	46/17653	1.44E-07	1.40E-05
GO:1901654	BP	GO:1901654	response t	55/2653	183/17653	1.65E-07	1.56E-05
GO:0007568	BP	GO:0007568	aging	79/2653	298/17653	1.66E-07	1.56E-05
GO:0035966	BP	GO:0035966	response t	58/2653	198/17653	2.04E-07	1.89E-05
GO:0071902	BP	GO:0071902	positive r	90/2653	355/17653	2.14E-07	1.96E-05
GO:0042692	BP	GO:0042692	muscle cel	92/2653	366/17653	2.39E-07	2.15E-05
GO:1901652	BP	GO:1901652	response t	116/2653	491/17653	2.43E-07	2.16E-05
GO:0051169	BP	GO:0051169	nuclear tr	89/2653	351/17653	2.48E-07	2.17E-05
GO:0034329	BP	GO:0034329	cell junct	63/2653	223/17653	2.63E-07	2.26E-05
GO:0048705	BP	GO:0048705	skeletal s	60/2653	209/17653	2.75E-07	2.33E-05
GO:0006913	BP	GO:0006913	nucleocyto	88/2653	347/17653	2.87E-07	2.35E-05
GO:0006914	BP	GO:0006914	autophagy	114/2653	482/17653	2.90E-07	2.35E-05
GO:0061919	BP	GO:0061919	process ut	114/2653	482/17653	2.90E-07	2.35E-05
GO:1902275	BP	GO:1902275	regulation	50/2653	163/17653	2.97E-07	2.35E-05
GO:0010634	BP	GO:0010634	positive r	48/2653	154/17653	3.00E-07	2.35E-05
GO:0071496	BP	GO:0071496	cellular r	80/2653	307/17653	3.00E-07	2.35E-05
GO:1901653	BP	GO:1901653	cellular r	89/2653	353/17653	3.24E-07	2.51E-05
GO:0009743	BP	GO:0009743	response t	62/2653	220/17653	3.58E-07	2.74E-05
GO:0018205	BP	GO:0018205	peptidyl-l	96/2653	390/17653	3.73E-07	2.81E-05
GO:0001101	BP	GO:0001101	response t	84/2653	329/17653	3.91E-07	2.91E-05
GO:0071383	BP	GO:0071383	cellular r	70/2653	260/17653	4.40E-07	3.24E-05
GO:0072001	BP	GO:0072001	renal syst	74/2653	280/17653	4.59E-07	3.33E-05
GO:0006986	BP	GO:0006986	response t	53/2653	179/17653	4.64E-07	3.33E-05
GO:0009791	BP	GO:0009791	post-embry	33/2653	91/17653	4.73E-07	3.35E-05
GO:0034284	BP	GO:0034284	response t	57/2653	198/17653	4.89E-07	3.41E-05
GO:0034333	BP	GO:0034333	adherens j	31/2653	83/17653	4.93E-07	3.41E-05
GO:0006479	BP	GO:0006479	protein me	53/2653	180/17653	5.63E-07	3.80E-05
GO:0008213	BP	GO:0008213	protein al	53/2653	180/17653	5.63E-07	3.80E-05
GO:0050684	BP	GO:0050684	regulation	39/2653	117/17653	5.68E-07	3.80E-05
GO:0007160	BP	GO:0007160	cell-matri	59/2653	209/17653	6.40E-07	4.24E-05
GO:0016236	BP	GO:0016236	macroautop	76/2653	293/17653	7.15E-07	4.68E-05
GO:0006888	BP	GO:0006888	ER to Golg	58/2653	205/17653	7.33E-07	4.75E-05
GO:0001654	BP	GO:0001654	eye develo	86/2653	344/17653	7.47E-07	4.79E-05
GO:1901861	BP	GO:1901861	regulation	45/2653	145/17653	7.95E-07	5.05E-05
GO:0050821	BP	GO:0050821	protein st	48/2653	159/17653	8.54E-07	5.37E-05
GO:0048738	BP	GO:0048738	cardiac mu	61/2653	221/17653	9.62E-07	5.98E-05
GO:0070997	BP	GO:0070997	neuron dea	80/2653	316/17653	1.06E-06	6.46E-05
GO:0033044	BP	GO:0033044	regulation	79/2653	311/17653	1.07E-06	6.46E-05
GO:0009746	BP	GO:0009746	response t	55/2653	193/17653	1.10E-06	6.46E-05
GO:0016579	BP	GO:0016579	protein de	75/2653	291/17653	1.10E-06	6.46E-05

GO:1903829	BP	GO:1903829	positive r	86/2653	347/17653	1.10E-06	6.46E-05
GO:1901214	BP	GO:1901214	regulation	73/2653	281/17653	1.10E-06	6.46E-05
GO:0043484	BP	GO:0043484	regulation	39/2653	120/17653	1.17E-06	6.80E-05
GO:0009749	BP	GO:0009749	response t	54/2653	189/17653	1.25E-06	7.18E-05
GO:0030335	BP	GO:0030335	positive r	112/2653	486/17653	1.43E-06	8.11E-05
GO:0051302	BP	GO:0051302	regulation	47/2653	157/17653	1.44E-06	8.11E-05
GO:0018210	BP	GO:0018210	peptidyl-t	43/2653	139/17653	1.52E-06	8.51E-05
GO:0007369	BP	GO:0007369	gastrulati	53/2653	186/17653	1.71E-06	9.37E-05
GO:0016311	BP	GO:0016311	dephosphor	107/2653	461/17653	1.72E-06	9.37E-05
GO:0048024	BP	GO:0048024	regulation	29/2653	79/17653	1.72E-06	9.37E-05
GO:0071359	BP	GO:0071359	cellular r	26/2653	67/17653	1.74E-06	9.37E-05
GO:0050673	BP	GO:0050673	epithelial	97/2653	409/17653	1.94E-06	0.0001033
GO:0034504	BP	GO:0034504	protein lc	66/2653	250/17653	1.95E-06	0.0001034
GO:0000079	BP	GO:0000079	regulation	31/2653	88/17653	2.13E-06	0.0001118
GO:0048762	BP	GO:0048762	mesenchyma	56/2653	202/17653	2.27E-06	0.0001181
GO:0035024	BP	GO:0035024	negative r	12/2653	19/17653	2.32E-06	0.0001195
GO:0043010	BP	GO:0043010	camera-typ	75/2653	297/17653	2.51E-06	0.0001281
GO:0060148	BP	GO:0060148	positive r	13/2653	22/17653	2.53E-06	0.0001281
GO:0043491	BP	GO:0043491	protein ki	66/2653	252/17653	2.63E-06	0.0001315
GO:1904029	BP	GO:1904029	regulation	32/2653	93/17653	2.64E-06	0.0001315
GO:0043405	BP	GO:0043405	regulation	84/2653	344/17653	2.76E-06	0.0001364
GO:2001234	BP	GO:2001234	negative r	58/2653	213/17653	2.81E-06	0.000138
GO:0019216	BP	GO:0019216	regulation	88/2653	366/17653	3.18E-06	0.0001544
GO:0034332	BP	GO:0034332	adherens j	40/2653	129/17653	3.26E-06	0.0001574
GO:0035967	BP	GO:0035967	cellular r	48/2653	166/17653	3.31E-06	0.0001583
GO:1903311	BP	GO:1903311	regulation	73/2653	289/17653	3.36E-06	0.0001594
GO:0016202	BP	GO:0016202	regulation	43/2653	143/17653	3.49E-06	0.0001643
GO:0008406	BP	GO:0008406	gonad deve	58/2653	215/17653	3.87E-06	0.0001808
GO:0048661	BP	GO:0048661	positive r	30/2653	86/17653	3.90E-06	0.0001808
GO:0061448	BP	GO:0061448	connective	63/2653	240/17653	4.05E-06	0.0001858
GO:0043401	BP	GO:0043401	steroid hc	53/2653	191/17653	4.07E-06	0.0001858
GO:0022613	BP	GO:0022613	ribonuclec	108/2653	475/17653	4.22E-06	0.0001914
GO:0034620	BP	GO:0034620	cellular r	44/2653	149/17653	4.57E-06	0.0002047
GO:0045137	BP	GO:0045137	developmen	59/2653	221/17653	4.58E-06	0.0002047
GO:0006352	BP	GO:0006352	DNA-templa	64/2653	246/17653	4.70E-06	0.0002084
GO:0051098	BP	GO:0051098	regulation	84/2653	349/17653	5.05E-06	0.0002222
GO:0031058	BP	GO:0031058	positive r	29/2653	83/17653	5.42E-06	0.0002368
GO:0035306	BP	GO:0035306	positive r	21/2653	51/17653	5.66E-06	0.0002453
GO:1905037	BP	GO:1905037	autophagos	32/2653	96/17653	5.70E-06	0.0002455
GO:2001236	BP	GO:2001236	regulation	45/2653	155/17653	5.89E-06	0.0002517
GO:0048872	BP	GO:0048872	homeostasi	62/2653	238/17653	6.28E-06	0.0002666
GO:0008584	BP	GO:0008584	male gonac	41/2653	137/17653	6.64E-06	0.0002798
GO:0010506	BP	GO:0010506	regulation	76/2653	310/17653	6.93E-06	0.0002902
GO:0001822	BP	GO:0001822	kidney dev	67/2653	264/17653	7.04E-06	0.0002929
GO:0051402	BP	GO:0051402	neuron apc	58/2653	219/17653	7.18E-06	0.0002964
GO:0030278	BP	GO:0030278	regulation	51/2653	185/17653	7.50E-06	0.0003077
GO:0048634	BP	GO:0048634	regulation	43/2653	147/17653	7.62E-06	0.0003086
GO:0001952	BP	GO:0001952	regulation	34/2653	106/17653	7.67E-06	0.0003086
GO:0010256	BP	GO:0010256	endomembra	98/2653	427/17653	7.68E-06	0.0003086
GO:0000045	BP	GO:0000045	autophagos	31/2653	93/17653	7.93E-06	0.0003168
GO:0070646	BP	GO:0070646	protein mc	75/2653	306/17653	8.02E-06	0.0003181

GO:0046546	BP	GO:0046546	developmer	41/2653	138/17653	8.09E-06	0.0003187
GO:0048008	BP	GO:0048008	platelet-c	21/2653	52/17653	8.15E-06	0.0003191
GO:2001235	BP	GO:2001235	positive r	49/2653	176/17653	8.37E-06	0.0003242
GO:0046777	BP	GO:0046777	protein au	61/2653	235/17653	8.39E-06	0.0003242
GO:0007044	BP	GO:0007044	cell-subst	30/2653	89/17653	8.59E-06	0.0003298
GO:0001701	BP	GO:0001701	in utero e	76/2653	312/17653	8.87E-06	0.0003385
GO:0045931	BP	GO:0045931	positive r	43/2653	148/17653	9.20E-06	0.0003487
GO:0031062	BP	GO:0031062	positive r	16/2653	34/17653	9.50E-06	0.0003574
GO:0030111	BP	GO:0030111	regulatior	82/2653	344/17653	9.59E-06	0.0003574
GO:0000910	BP	GO:0000910	cytokinesi	48/2653	172/17653	9.60E-06	0.0003574
GO:0010821	BP	GO:0010821	regulatior	59/2653	226/17653	9.70E-06	0.0003587
GO:0061640	BP	GO:0061640	cytoskelet	28/2653	81/17653	9.88E-06	0.0003631
GO:0060065	BP	GO:0060065	uterus dev	12/2653	21/17653	1.01E-05	0.0003658
GO:2000637	BP	GO:2000637	positive r	12/2653	21/17653	1.01E-05	0.0003658
GO:0000381	BP	GO:0000381	regulatior	19/2653	45/17653	1.01E-05	0.000366
GO:0006605	BP	GO:0006605	protein ta	96/2653	419/17653	1.02E-05	0.000366
GO:2001237	BP	GO:2001237	negative r	33/2653	103/17653	1.07E-05	0.0003788
GO:0007517	BP	GO:0007517	muscle org	92/2653	398/17653	1.07E-05	0.0003788
GO:0060485	BP	GO:0060485	mesenchyme	65/2653	257/17653	1.08E-05	0.0003809
GO:0060996	BP	GO:0060996	dendritic	30/2653	90/17653	1.10E-05	0.000385
GO:1905269	BP	GO:1905269	positive r	30/2653	90/17653	1.10E-05	0.000385
GO:1903747	BP	GO:1903747	regulatior	38/2653	126/17653	1.17E-05	0.0004025
GO:0061614	BP	GO:0061614	pri-miRNA	17/2653	38/17653	1.17E-05	0.0004025
GO:0000077	BP	GO:0000077	DNA damage	45/2653	159/17653	1.22E-05	0.0004165
GO:0030198	BP	GO:0030198	extracellu	81/2653	341/17653	1.24E-05	0.0004235
GO:0001763	BP	GO:0001763	morphogene	53/2653	198/17653	1.26E-05	0.0004241
GO:0016241	BP	GO:0016241	regulatior	46/2653	164/17653	1.26E-05	0.0004241
GO:0050678	BP	GO:0050678	regulatior	84/2653	357/17653	1.27E-05	0.0004241
GO:0051168	BP	GO:0051168	nuclear ex	54/2653	203/17653	1.27E-05	0.0004247
GO:0045445	BP	GO:0045445	myoblast d	28/2653	82/17653	1.29E-05	0.0004262
GO:0031570	BP	GO:0031570	DNA integr	47/2653	169/17653	1.31E-05	0.0004282
GO:0070507	BP	GO:0070507	regulatior	47/2653	169/17653	1.31E-05	0.0004282
GO:0051146	BP	GO:0051146	striated m	69/2653	279/17653	1.32E-05	0.0004307
GO:0034968	BP	GO:0034968	histone ly	33/2653	104/17653	1.34E-05	0.0004335
GO:0071156	BP	GO:0071156	regulatior	35/2653	113/17653	1.34E-05	0.0004335
GO:0046677	BP	GO:0046677	response t	78/2653	326/17653	1.36E-05	0.0004356
GO:0043523	BP	GO:0043523	regulatior	52/2653	194/17653	1.46E-05	0.0004624
GO:0043536	BP	GO:0043536	positive r	26/2653	74/17653	1.46E-05	0.0004624
GO:0016571	BP	GO:0016571	histone me	39/2653	132/17653	1.53E-05	0.0004844
GO:0002011	BP	GO:0002011	morphogene	21/2653	54/17653	1.63E-05	0.000512
GO:0051236	BP	GO:0051236	establishm	53/2653	200/17653	1.71E-05	0.0005354
GO:1903312	BP	GO:1903312	negative r	26/2653	75/17653	1.91E-05	0.0005949
GO:0032963	BP	GO:0032963	collagen m	37/2653	124/17653	1.97E-05	0.0006072
GO:0030518	BP	GO:0030518	intracellu	40/2653	138/17653	1.97E-05	0.0006072
GO:0036294	BP	GO:0036294	cellular r	54/2653	206/17653	2.00E-05	0.0006123
GO:0000281	BP	GO:0000281	mitotic cy	24/2653	67/17653	2.13E-05	0.0006443
GO:0071479	BP	GO:0071479	cellular r	24/2653	67/17653	2.13E-05	0.0006443
GO:0030099	BP	GO:0030099	myeloid ce	90/2653	394/17653	2.15E-05	0.0006493
GO:0042594	BP	GO:0042594	response t	48/2653	177/17653	2.20E-05	0.0006587
GO:0006367	BP	GO:0006367	transcript	50/2653	187/17653	2.26E-05	0.0006672
GO:0010332	BP	GO:0010332	response t	21/2653	55/17653	2.27E-05	0.0006672

GO:0007548	BP	GO:0007548	sex differ	67/2653	273/17653	2.28E-05	0.0006672
GO:0050679	BP	GO:0050679	positive r	51/2653	192/17653	2.29E-05	0.0006672
GO:0071453	BP	GO:0071453	cellular r	57/2653	222/17653	2.30E-05	0.0006672
GO:0050657	BP	GO:0050657	nucleic ac	52/2653	197/17653	2.30E-05	0.0006672
GO:0050658	BP	GO:0050658	RNA transp	52/2653	197/17653	2.30E-05	0.0006672
GO:0010464	BP	GO:0010464	regulation	16/2653	36/17653	2.33E-05	0.00067
GO:0043414	BP	GO:0043414	macromolec	71/2653	294/17653	2.34E-05	0.00067
GO:0030168	BP	GO:0030168	platelet a	44/2653	158/17653	2.34E-05	0.00067
GO:0009267	BP	GO:0009267	cellular r	40/2653	139/17653	2.37E-05	0.0006753
GO:0050686	BP	GO:0050686	negative r	14/2653	29/17653	2.40E-05	0.0006781
GO:0003007	BP	GO:0003007	heart morp	61/2653	243/17653	2.53E-05	0.0007139
GO:0051817	BP	GO:0051817	modificati	33/2653	107/17653	2.58E-05	0.0007247
GO:1903035	BP	GO:1903035	negative r	28/2653	85/17653	2.74E-05	0.0007656
GO:2001252	BP	GO:2001252	positive r	44/2653	159/17653	2.78E-05	0.0007715
GO:0006397	BP	GO:0006397	mRNA proce	108/2653	495/17653	2.83E-05	0.0007764
GO:0009991	BP	GO:0009991	response t	108/2653	495/17653	2.83E-05	0.0007764
GO:0042326	BP	GO:0042326	negative r	104/2653	473/17653	2.83E-05	0.0007764
GO:0061326	BP	GO:0061326	renal tubu	30/2653	94/17653	2.87E-05	0.0007827
GO:1902751	BP	GO:1902751	positive r	13/2653	26/17653	2.92E-05	0.000794
GO:0031098	BP	GO:0031098	stress-act	71/2653	296/17653	2.97E-05	0.0007999
GO:0009755	BP	GO:0009755	hormone-me	59/2653	234/17653	2.97E-05	0.0007999
GO:1904030	BP	GO:1904030	negative r	15/2653	33/17653	3.03E-05	0.0008112
GO:0010718	BP	GO:0010718	positive r	19/2653	48/17653	3.05E-05	0.0008145
GO:0010212	BP	GO:0010212	response t	42/2653	150/17653	3.09E-05	0.0008154
GO:0035051	BP	GO:0035051	cardiocyte	42/2653	150/17653	3.09E-05	0.0008154
GO:0071456	BP	GO:0071456	cellular r	52/2653	199/17653	3.10E-05	0.0008154
GO:0042176	BP	GO:0042176	regulation	82/2653	355/17653	3.24E-05	0.000849
GO:0045862	BP	GO:0045862	positive r	81/2653	350/17653	3.36E-05	0.0008768
GO:0010586	BP	GO:0010586	miRNA meta	12/2653	23/17653	3.45E-05	0.0008932
GO:0000075	BP	GO:0000075	cell cycle	57/2653	225/17653	3.48E-05	0.0008932
GO:0007045	BP	GO:0007045	cell-subst	25/2653	73/17653	3.48E-05	0.0008932
GO:0048041	BP	GO:0048041	focal adhe	25/2653	73/17653	3.48E-05	0.0008932
GO:0016572	BP	GO:0016572	histone ph	16/2653	37/17653	3.54E-05	0.0009028
GO:0061138	BP	GO:0061138	morphogene	49/2653	185/17653	3.55E-05	0.0009028
GO:0018022	BP	GO:0018022	peptidyl-l	35/2653	118/17653	3.75E-05	0.0009498
GO:1904888	BP	GO:1904888	cranial sk	23/2653	65/17653	3.93E-05	0.0009878
GO:1903362	BP	GO:1903362	regulation	58/2653	231/17653	3.93E-05	0.0009878
GO:0032869	BP	GO:0032869	cellular r	52/2653	201/17653	4.14E-05	0.0010353
GO:0071260	BP	GO:0071260	cellular r	26/2653	78/17653	4.17E-05	0.0010382
GO:0000380	BP	GO:0000380	alternativ	21/2653	57/17653	4.24E-05	0.0010455
GO:1903391	BP	GO:1903391	regulation	21/2653	57/17653	4.24E-05	0.0010455
GO:0010595	BP	GO:0010595	positive r	34/2653	114/17653	4.25E-05	0.0010455
GO:0010171	BP	GO:0010171	body morph	19/2653	49/17653	4.29E-05	0.0010511
GO:0035065	BP	GO:0035065	regulation	20/2653	53/17653	4.31E-05	0.0010513
GO:0010717	BP	GO:0010717	regulation	28/2653	87/17653	4.42E-05	0.0010618
GO:1901989	BP	GO:1901989	positive r	28/2653	87/17653	4.42E-05	0.0010618
GO:0031668	BP	GO:0031668	cellular r	59/2653	237/17653	4.42E-05	0.0010618
GO:0010508	BP	GO:0010508	positive r	32/2653	105/17653	4.42E-05	0.0010618
GO:0034109	BP	GO:0034109	homotypic	25/2653	74/17653	4.51E-05	0.0010801
GO:0045732	BP	GO:0045732	positive r	54/2653	212/17653	4.71E-05	0.0011221
GO:0007179	BP	GO:0007179	transformi	48/2653	182/17653	4.77E-05	0.0011289

GO:0032886	BP	GO:0032886	regulatior	51/2653	197/17653	4.79E-05	0.0011289
GO:0072655	BP	GO:0072655	establishm	50/2653	192/17653	4.79E-05	0.0011289
GO:0045995	BP	GO:0045995	regulatior	36/2653	124/17653	4.84E-05	0.0011348
GO:0034976	BP	GO:0034976	response t	66/2653	274/17653	4.86E-05	0.0011348
GO:0001933	BP	GO:0001933	negative r	96/2653	435/17653	4.88E-05	0.0011366
GO:0007265	BP	GO:0007265	Ras protei	101/2653	463/17653	5.11E-05	0.0011749
GO:0006403	BP	GO:0006403	RNA locali	58/2653	233/17653	5.11E-05	0.0011749
GO:0046879	BP	GO:0046879	hormone se	72/2653	306/17653	5.12E-05	0.0011749
GO:0048754	BP	GO:0048754	branching	42/2653	153/17653	5.13E-05	0.0011749
GO:0045785	BP	GO:0045785	positive r	88/2653	392/17653	5.20E-05	0.0011877
GO:0003205	BP	GO:0003205	cardiac ch	43/2653	158/17653	5.24E-05	0.0011896
GO:0035307	BP	GO:0035307	positive r	16/2653	38/17653	5.27E-05	0.0011896
GO:0040019	BP	GO:0040019	positive r	16/2653	38/17653	5.27E-05	0.0011896
GO:0021543	BP	GO:0021543	pallium de	44/2653	163/17653	5.34E-05	0.0011973
GO:0060070	BP	GO:0060070	canonical	74/2653	317/17653	5.34E-05	0.0011973
GO:0032868	BP	GO:0032868	response t	62/2653	254/17653	5.36E-05	0.0011973
GO:0031669	BP	GO:0031669	cellular r	53/2653	208/17653	5.45E-05	0.0012128
GO:0016239	BP	GO:0016239	positive r	22/2653	62/17653	5.47E-05	0.0012131
GO:0017038	BP	GO:0017038	protein im	51/2653	198/17653	5.52E-05	0.001219
GO:0060541	BP	GO:0060541	respirator	50/2653	193/17653	5.54E-05	0.001219
GO:0002062	BP	GO:0002062	chondrocyt	30/2653	97/17653	5.57E-05	0.0012221
GO:2000756	BP	GO:2000756	regulatior	21/2653	58/17653	5.71E-05	0.0012481
GO:0051058	BP	GO:0051058	negative r	20/2653	54/17653	5.88E-05	0.0012635
GO:0051893	BP	GO:0051893	regulatior	20/2653	54/17653	5.88E-05	0.0012635
GO:0090109	BP	GO:0090109	regulatior	20/2653	54/17653	5.88E-05	0.0012635
GO:0090183	BP	GO:0090183	regulatior	20/2653	54/17653	5.88E-05	0.0012635
GO:0010948	BP	GO:0010948	negative r	80/2653	350/17653	5.89E-05	0.0012635
GO:0010922	BP	GO:0010922	positive r	14/2653	31/17653	6.09E-05	0.0012879
GO:0045736	BP	GO:0045736	negative r	14/2653	31/17653	6.09E-05	0.0012879
GO:0031667	BP	GO:0031667	response t	101/2653	465/17653	6.10E-05	0.0012879
GO:0030968	BP	GO:0030968	endoplasmi	37/2653	130/17653	6.11E-05	0.0012879
GO:1901987	BP	GO:1901987	regulatior	99/2653	454/17653	6.13E-05	0.0012879
GO:0048511	BP	GO:0048511	rhythmic p	66/2653	276/17653	6.15E-05	0.0012879
GO:0051781	BP	GO:0051781	positive r	27/2653	84/17653	6.15E-05	0.0012879
GO:0031571	BP	GO:0031571	mitotic G1	24/2653	71/17653	6.30E-05	0.0013054
GO:0044819	BP	GO:0044819	mitotic G1	24/2653	71/17653	6.30E-05	0.0013054
GO:0090114	BP	GO:0090114	COPII-coat	24/2653	71/17653	6.30E-05	0.0013054
GO:0010631	BP	GO:0010631	epithelial	75/2653	324/17653	6.40E-05	0.0013196
GO:0051216	BP	GO:0051216	cartilage	48/2653	184/17653	6.41E-05	0.0013196
GO:0003231	BP	GO:0003231	cardiac ve	35/2653	121/17653	6.65E-05	0.0013587
GO:0055007	BP	GO:0055007	cardiac mu	35/2653	121/17653	6.65E-05	0.0013587
GO:0031330	BP	GO:0031330	negative r	57/2653	230/17653	6.74E-05	0.0013728
GO:0001656	BP	GO:0001656	metanephrc	28/2653	89/17653	6.96E-05	0.0014127
GO:0006622	BP	GO:0006622	protein ta	11/2653	21/17653	7.05E-05	0.001426
GO:0072331	BP	GO:0072331	signal tra	65/2653	272/17653	7.11E-05	0.0014341
GO:0051988	BP	GO:0051988	regulatior	8/2653	12/17653	7.22E-05	0.0014505
GO:0007519	BP	GO:0007519	skeletal m	44/2653	165/17653	7.30E-05	0.0014615
GO:0070585	BP	GO:0070585	protein lc	50/2653	195/17653	7.36E-05	0.0014693
GO:0070527	BP	GO:0070527	platelet a	20/2653	55/17653	7.95E-05	0.0015813
GO:0044783	BP	GO:0044783	G1 DNA dam	24/2653	72/17653	8.12E-05	0.0016099
GO:0022618	BP	GO:0022618	ribonuclec	53/2653	211/17653	8.19E-05	0.0016185

GO:0002064	BP	GO:0002064	epithelial	51/2653	201/17653	8.38E-05	0.001652
GO:1903034	BP	GO:1903034	regulation	43/2653	161/17653	8.43E-05	0.0016531
GO:0043331	BP	GO:0043331	response t	30/2653	99/17653	8.48E-05	0.0016531
GO:0009266	BP	GO:0009266	response t	58/2653	237/17653	8.51E-05	0.0016531
GO:0021537	BP	GO:0021537	telencepha	58/2653	237/17653	8.51E-05	0.0016531
GO:0031099	BP	GO:0031099	regenerati	48/2653	186/17653	8.56E-05	0.0016531
GO:1901888	BP	GO:1901888	regulation	26/2653	81/17653	8.58E-05	0.0016531
GO:0006611	BP	GO:0006611	protein ex	47/2653	181/17653	8.58E-05	0.0016531
GO:1903749	BP	GO:1903749	positive r	33/2653	113/17653	8.64E-05	0.0016584
GO:0031124	BP	GO:0031124	mRNA 3'-en	28/2653	90/17653	8.67E-05	0.0016584
GO:0034605	BP	GO:0034605	cellular r	37/2653	132/17653	8.69E-05	0.0016584
GO:0090132	BP	GO:0090132	epithelium	75/2653	327/17653	8.78E-05	0.0016701
GO:0001889	BP	GO:0001889	liver deve	38/2653	137/17653	8.98E-05	0.0017024
GO:1902893	BP	GO:1902893	regulation	14/2653	32/17653	9.33E-05	0.0017635
GO:0090130	BP	GO:0090130	tissue mig	76/2653	333/17653	9.37E-05	0.0017649
GO:0010721	BP	GO:0010721	negative r	70/2653	301/17653	9.49E-05	0.0017826
GO:1990823	BP	GO:1990823	response t	29/2653	95/17653	9.56E-05	0.0017845
GO:1990830	BP	GO:1990830	cellular r	29/2653	95/17653	9.56E-05	0.0017845
GO:0032147	BP	GO:0032147	activation	75/2653	328/17653	9.74E-05	0.0018089
GO:0032259	BP	GO:0032259	methylatio	80/2653	355/17653	9.75E-05	0.0018089
GO:0060348	BP	GO:0060348	bone devel	48/2653	187/17653	9.87E-05	0.0018244
GO:0002753	BP	GO:0002753	cytoplasmi	21/2653	60/17653	0.0001009	0.0018539
GO:0010812	BP	GO:0010812	negative r	21/2653	60/17653	0.0001009	0.0018539
GO:0071826	BP	GO:0071826	ribonucleo	55/2653	223/17653	0.0001027	0.0018825
GO:0045740	BP	GO:0045740	positive r	20/2653	56/17653	0.0001063	0.0019422
GO:0022612	BP	GO:0022612	gland morp	34/2653	119/17653	0.0001097	0.0019935
GO:0051271	BP	GO:0051271	negative r	77/2653	340/17653	0.0001103	0.0019935
GO:0017145	BP	GO:0017145	stem cell	16/2653	40/17653	0.0001104	0.0019935
GO:0090184	BP	GO:0090184	positive r	16/2653	40/17653	0.0001104	0.0019935
GO:0002088	BP	GO:0002088	lens devel	23/2653	69/17653	0.0001134	0.0020408
GO:1903706	BP	GO:1903706	regulation	97/2653	450/17653	0.0001139	0.002043
GO:0060538	BP	GO:0060538	skeletal m	45/2653	173/17653	0.0001149	0.0020501
GO:0061136	BP	GO:0061136	regulation	45/2653	173/17653	0.0001149	0.0020501
GO:0017015	BP	GO:0017015	regulation	32/2653	110/17653	0.000119	0.0021157
GO:1901216	BP	GO:1901216	positive r	25/2653	78/17653	0.0001196	0.0021207
GO:0048145	BP	GO:0048145	regulation	27/2653	87/17653	0.0001206	0.0021314
GO:0097150	BP	GO:0097150	neuronal s	11/2653	22/17653	0.0001218	0.0021446
GO:0000209	BP	GO:0000209	protein po	69/2653	298/17653	0.000122	0.0021446
GO:0070306	BP	GO:0070306	lens fiber	13/2653	29/17653	0.0001225	0.0021472
GO:0015931	BP	GO:0015931	nucleobase	58/2653	240/17653	0.0001229	0.0021474
GO:0007178	BP	GO:0007178	transmembr	76/2653	336/17653	0.0001269	0.0022116
GO:0072593	BP	GO:0072593	reactive o	61/2653	256/17653	0.0001273	0.0022116
GO:0023061	BP	GO:0023061	signal rel	94/2653	435/17653	0.0001313	0.0022745
GO:0072080	BP	GO:0072080	nephron tu	28/2653	92/17653	0.0001326	0.0022878
GO:0009408	BP	GO:0009408	response t	45/2653	174/17653	0.0001328	0.0022878
GO:0033148	BP	GO:0033148	positive r	7/2653	10/17653	0.0001355	0.0023273
GO:0001704	BP	GO:0001704	formation	35/2653	125/17653	0.0001363	0.0023345
GO:1901990	BP	GO:1901990	regulation	91/2653	419/17653	0.0001385	0.0023658
GO:0008608	BP	GO:0008608	attachment	14/2653	33/17653	0.0001398	0.0023675
GO:0071214	BP	GO:0071214	cellular r	71/2653	310/17653	0.0001398	0.0023675
GO:0104004	BP	GO:0104004	cellular r	71/2653	310/17653	0.0001398	0.0023675

GO:0045444	BP	GO:0045444	fat cell c	52/2653	210/17653	0.0001405	0.0023725
GO:0034250	BP	GO:0034250	positive r	38/2653	140/17653	0.0001474	0.002476
GO:0061008	BP	GO:0061008	hepaticobi	38/2653	140/17653	0.0001474	0.002476
GO:0048144	BP	GO:0048144	fibroblast	27/2653	88/17653	0.0001493	0.0024936
GO:0060349	BP	GO:0060349	bone morph	27/2653	88/17653	0.0001493	0.0024936
GO:0043406	BP	GO:0043406	positive r	63/2653	268/17653	0.0001502	0.0024958
GO:0007492	BP	GO:0007492	endoderm c	25/2653	79/17653	0.0001503	0.0024958
GO:0046661	BP	GO:0046661	male sex c	42/2653	160/17653	0.0001537	0.0025341
GO:0051099	BP	GO:0051099	positive r	44/2653	170/17653	0.0001538	0.0025341
GO:0099518	BP	GO:0099518	vesicle cy	18/2653	49/17653	0.0001538	0.0025341
GO:0006275	BP	GO:0006275	regulation	34/2653	121/17653	0.0001565	0.0025608
GO:0043467	BP	GO:0043467	regulation	34/2653	121/17653	0.0001565	0.0025608
GO:0048538	BP	GO:0048538	thymus dev	17/2653	45/17653	0.0001567	0.0025608
GO:0033137	BP	GO:0033137	negative r	12/2653	26/17653	0.0001587	0.0025865
GO:0090110	BP	GO:0090110	cargo load	8/2653	13/17653	0.0001629	0.0026464
GO:0044773	BP	GO:0044773	mitotic DN	31/2653	107/17653	0.0001638	0.002654
GO:1903050	BP	GO:1903050	regulation	50/2653	201/17653	0.0001657	0.0026778
GO:0030900	BP	GO:0030900	forebrain	81/2653	366/17653	0.0001664	0.0026825
GO:0022407	BP	GO:0022407	regulation	85/2653	388/17653	0.0001671	0.0026873
GO:0010632	BP	GO:0010632	regulation	63/2653	269/17653	0.0001679	0.0026915
GO:0006977	BP	GO:0006977	DNA damage	21/2653	62/17653	0.0001719	0.0027467
GO:1903844	BP	GO:1903844	regulation	32/2653	112/17653	0.0001722	0.0027467
GO:0048736	BP	GO:0048736	appendage	45/2653	176/17653	0.0001763	0.0027973
GO:0060173	BP	GO:0060173	limb devel	45/2653	176/17653	0.0001763	0.0027973
GO:0050768	BP	GO:0050768	negative r	61/2653	259/17653	0.0001793	0.0028379
GO:0046486	BP	GO:0046486	glycerolip	96/2653	450/17653	0.0001825	0.0028733
GO:0009914	BP	GO:0009914	hormone tr	72/2653	318/17653	0.0001825	0.0028733
GO:0046685	BP	GO:0046685	response t	13/2653	30/17653	0.0001866	0.0029143
GO:1900739	BP	GO:1900739	regulation	13/2653	30/17653	0.0001866	0.0029143
GO:1900740	BP	GO:1900740	positive r	13/2653	30/17653	0.0001866	0.0029143
GO:0048194	BP	GO:0048194	Golgi vesi	25/2653	80/17653	0.0001877	0.0029249
GO:0006402	BP	GO:0006402	mRNA catab	78/2653	351/17653	0.0001899	0.0029504
GO:0006401	BP	GO:0006401	RNA catabo	84/2653	384/17653	0.0001916	0.0029689
GO:0051896	BP	GO:0051896	regulation	53/2653	218/17653	0.0002005	0.0030993
GO:0003281	BP	GO:0003281	ventricula	22/2653	67/17653	0.0002029	0.0031284
GO:0030324	BP	GO:0030324	lung devel	43/2653	167/17653	0.0002055	0.0031521
GO:0046890	BP	GO:0046890	regulation	43/2653	167/17653	0.0002055	0.0031521
GO:0051568	BP	GO:0051568	histone H3	18/2653	50/17653	0.0002069	0.003166
GO:0045778	BP	GO:0045778	positive r	26/2653	85/17653	0.0002075	0.0031664
GO:0042110	BP	GO:0042110	T cell act	98/2653	463/17653	0.0002118	0.0032239
GO:1904019	BP	GO:1904019	epithelial	29/2653	99/17653	0.000213	0.0032342
GO:0032007	BP	GO:0032007	negative r	17/2653	46/17653	0.0002141	0.0032348
GO:0007223	BP	GO:0007223	Wnt signal	15/2653	38/17653	0.0002146	0.0032348
GO:1901030	BP	GO:1901030	positive r	15/2653	38/17653	0.0002146	0.0032348
GO:0030010	BP	GO:0030010	establishm	34/2653	123/17653	0.0002206	0.0033169
GO:0072431	BP	GO:0072431	signal tra	21/2653	63/17653	0.0002218	0.0033177
GO:1902400	BP	GO:1902400	intracellu	21/2653	63/17653	0.0002218	0.0033177
GO:0033157	BP	GO:0033157	regulation	61/2653	261/17653	0.0002241	0.0033439
GO:0061045	BP	GO:0061045	negative r	23/2653	72/17653	0.000233	0.003468
GO:1903364	BP	GO:1903364	positive r	37/2653	138/17653	0.000234	0.0034751
GO:0010822	BP	GO:0010822	positive r	43/2653	168/17653	0.0002366	0.0035055

GO:0009314	BP	GO:0009314	response t	94/2653	442/17653	0.0002375	0.0035091
GO:0048015	BP	GO:0048015	phosphatic	41/2653	158/17653	0.0002387	0.0035192
GO:0031060	BP	GO:0031060	regulation	20/2653	59/17653	0.0002406	0.0035298
GO:1902041	BP	GO:1902041	regulation	20/2653	59/17653	0.0002406	0.0035298
GO:0045667	BP	GO:0045667	regulation	32/2653	114/17653	0.0002459	0.0035977
GO:0031954	BP	GO:0031954	positive r	12/2653	27/17653	0.0002468	0.003603
GO:0051961	BP	GO:0051961	negative r	64/2653	278/17653	0.0002496	0.0036353
GO:0045666	BP	GO:0045666	positive r	76/2653	343/17653	0.0002504	0.0036384
GO:0048662	BP	GO:0048662	negative r	19/2653	55/17653	0.0002588	0.0037503
GO:0008380	BP	GO:0008380	RNA splici	92/2653	432/17653	0.0002618	0.0037857
GO:0001503	BP	GO:0001503	ossificati	81/2653	371/17653	0.0002633	0.0037984
GO:2000146	BP	GO:2000146	negative r	70/2653	311/17653	0.0002672	0.003845
GO:0061041	BP	GO:0061041	regulation	36/2653	134/17653	0.0002704	0.0038815
GO:0002521	BP	GO:0002521	leukocyte	101/2653	483/17653	0.0002715	0.0038857
GO:0043534	BP	GO:0043534	blood vess	43/2653	169/17653	0.000272	0.0038857
GO:0002262	BP	GO:0002262	myeloid ce	37/2653	139/17653	0.0002735	0.0038857
GO:0097327	BP	GO:0097327	response t	27/2653	91/17653	0.0002756	0.0038857
GO:0007033	BP	GO:0007033	vacuole or	41/2653	159/17653	0.0002757	0.0038857
GO:0035107	BP	GO:0035107	appendage	39/2653	149/17653	0.0002764	0.0038857
GO:0035108	BP	GO:0035108	limb morph	39/2653	149/17653	0.0002764	0.0038857
GO:0051028	BP	GO:0051028	mRNA trans	39/2653	149/17653	0.0002764	0.0038857
GO:0038127	BP	GO:0038127	ERBB signa	40/2653	154/17653	0.0002765	0.0038857
GO:0030330	BP	GO:0030330	DNA damage	31/2653	110/17653	0.0002824	0.0039592
GO:2000573	BP	GO:2000573	positive r	21/2653	64/17653	0.0002839	0.0039707
GO:1901028	BP	GO:1901028	regulation	17/2653	47/17653	0.0002889	0.0040315
GO:0051204	BP	GO:0051204	protein in	14/2653	35/17653	0.0002946	0.0041021
GO:0016358	BP	GO:0016358	dendrite d	52/2653	216/17653	0.0002959	0.0041106
GO:0010642	BP	GO:0010642	negative r	9/2653	17/17653	0.000297	0.0041161
GO:0030336	BP	GO:0030336	negative r	67/2653	296/17653	0.0003024	0.004181
GO:0051701	BP	GO:0051701	interactio	51/2653	211/17653	0.0003038	0.0041914
GO:1904951	BP	GO:1904951	positive r	97/2653	462/17653	0.0003066	0.0042095
GO:0032355	BP	GO:0032355	response t	34/2653	125/17653	0.0003072	0.0042095
GO:0035270	BP	GO:0035270	endocrine	34/2653	125/17653	0.0003072	0.0042095
GO:0043062	BP	GO:0043062	extracellu	85/2653	395/17653	0.0003102	0.0042402
GO:0048013	BP	GO:0048013	ephrin rec	26/2653	87/17653	0.0003129	0.0042577
GO:0060021	BP	GO:0060021	roof of mo	26/2653	87/17653	0.0003129	0.0042577
GO:0022604	BP	GO:0022604	regulation	98/2653	468/17653	0.0003156	0.0042746
GO:0035690	BP	GO:0035690	cellular r	75/2653	340/17653	0.0003157	0.0042746
GO:0001678	BP	GO:0001678	cellular g	36/2653	135/17653	0.0003162	0.0042746
GO:0072073	BP	GO:0072073	kidney epi	37/2653	140/17653	0.0003187	0.0042989
GO:0030072	BP	GO:0030072	peptide ho	57/2653	243/17653	0.0003202	0.0043083
GO:0003171	BP	GO:0003171	atrioventr	11/2653	24/17653	0.0003223	0.0043083
GO:0010640	BP	GO:0010640	regulation	11/2653	24/17653	0.0003223	0.0043083
GO:0033119	BP	GO:0033119	negative r	11/2653	24/17653	0.0003223	0.0043083
GO:1902894	BP	GO:1902894	negative r	7/2653	11/17653	0.0003241	0.0043118
GO:0072401	BP	GO:0072401	signal tra	24/2653	78/17653	0.0003247	0.0043118
GO:0072422	BP	GO:0072422	signal tra	24/2653	78/17653	0.0003247	0.0043118
GO:1901983	BP	GO:1901983	regulation	22/2653	69/17653	0.0003255	0.0043124
GO:0031054	BP	GO:0031054	pre-miRNA	8/2653	14/17653	0.0003299	0.0043611
GO:0031929	BP	GO:0031929	TOR signal	31/2653	111/17653	0.0003362	0.0044359
GO:2000027	BP	GO:2000027	regulation	58/2653	249/17653	0.0003456	0.0045402

GO:0051101	BP	GO:0051101	regulation	32/2653	116/17653	0.0003463	0.0045402
GO:0002221	BP	GO:0002221	pattern re	45/2653	181/17653	0.0003464	0.0045402
GO:0050769	BP	GO:0050769	positive r	91/2653	430/17653	0.0003518	0.0046014
GO:0008625	BP	GO:0008625	extrinsic	25/2653	83/17653	0.0003545	0.004619
GO:0006900	BP	GO:0006900	vesicle bu	28/2653	97/17653	0.0003547	0.004619
GO:0030323	BP	GO:0030323	respirator	43/2653	171/17653	0.0003573	0.0046202
GO:0045637	BP	GO:0045637	regulation	57/2653	244/17653	0.0003578	0.0046202
GO:0072413	BP	GO:0072413	signal tra	21/2653	65/17653	0.0003607	0.0046202
GO:1902402	BP	GO:1902402	signal tra	21/2653	65/17653	0.0003607	0.0046202
GO:1902403	BP	GO:1902403	signal tra	21/2653	65/17653	0.0003607	0.0046202
GO:0030705	BP	GO:0030705	cytoskelet	42/2653	166/17653	0.0003618	0.0046202
GO:0007059	BP	GO:0007059	chromosome	78/2653	358/17653	0.000362	0.0046202
GO:0035904	BP	GO:0035904	aorta deve	18/2653	52/17653	0.0003625	0.0046202
GO:0046626	BP	GO:0046626	regulation	18/2653	52/17653	0.0003625	0.0046202
GO:0048641	BP	GO:0048641	regulation	18/2653	52/17653	0.0003625	0.0046202
GO:0048017	BP	GO:0048017	inositol l	41/2653	161/17653	0.0003655	0.0046486
GO:0001823	BP	GO:0001823	mesonephro	29/2653	102/17653	0.0003719	0.0046969
GO:0046854	BP	GO:0046854	phosphatic	29/2653	102/17653	0.0003719	0.0046969
GO:0002053	BP	GO:0002053	positive r	12/2653	28/17653	0.0003732	0.0046969
GO:0033598	BP	GO:0033598	mammary gl	12/2653	28/17653	0.0003732	0.0046969
GO:0071480	BP	GO:0071480	cellular r	12/2653	28/17653	0.0003732	0.0046969
GO:0033500	BP	GO:0033500	carbohydra	52/2653	218/17653	0.0003749	0.0046994
GO:0042593	BP	GO:0042593	glucose ho	52/2653	218/17653	0.0003749	0.0046994
GO:0040013	BP	GO:0040013	negative r	77/2653	353/17653	0.0003791	0.0047425
GO:0071158	BP	GO:0071158	positive r	26/2653	88/17653	0.0003814	0.0047513
GO:0110020	BP	GO:0110020	regulation	26/2653	88/17653	0.0003814	0.0047513
GO:0046580	BP	GO:0046580	negative r	17/2653	48/17653	0.0003853	0.00477
GO:0060324	BP	GO:0060324	face devel	17/2653	48/17653	0.0003853	0.00477
GO:0000086	BP	GO:0000086	G2/M trans	58/2653	250/17653	0.0003853	0.00477
GO:0072395	BP	GO:0072395	signal tra	24/2653	79/17653	0.0004008	0.0049516
GO:0001844	BP	GO:0001844	protein in	13/2653	32/17653	0.0004033	0.0049727
GO:0003181	BP	GO:0003181	atrioventr	10/2653	21/17653	0.0004131	0.0050563
GO:2000114	BP	GO:2000114	regulation	10/2653	21/17653	0.0004131	0.0050563
GO:1901617	BP	GO:1901617	organic hy	56/2653	240/17653	0.0004135	0.0050563
GO:0051147	BP	GO:0051147	regulation	42/2653	167/17653	0.0004143	0.0050563
GO:0050863	BP	GO:0050863	regulation	71/2653	321/17653	0.0004143	0.0050563
GO:0014009	BP	GO:0014009	glial cell	14/2653	36/17653	0.0004159	0.005066
GO:0044839	BP	GO:0044839	cell cycle	61/2653	267/17653	0.0004254	0.0051309
GO:0051403	BP	GO:0051403	stress-act	61/2653	267/17653	0.0004254	0.0051309
GO:0006476	BP	GO:0006476	protein de	28/2653	98/17653	0.0004264	0.0051309
GO:0072163	BP	GO:0072163	mesonephri	28/2653	98/17653	0.0004264	0.0051309
GO:0072164	BP	GO:0072164	mesonephri	28/2653	98/17653	0.0004264	0.0051309
GO:0018393	BP	GO:0018393	internal p	39/2653	152/17653	0.0004269	0.0051309
GO:1902806	BP	GO:1902806	regulation	47/2653	193/17653	0.0004286	0.0051309
GO:0016573	BP	GO:0016573	histone ac	38/2653	147/17653	0.000429	0.0051309
GO:0072006	BP	GO:0072006	nephron de	36/2653	137/17653	0.0004291	0.0051309
GO:0071901	BP	GO:0071901	negative r	37/2653	142/17653	0.0004298	0.0051309
GO:0001776	BP	GO:0001776	leukocyte	25/2653	84/17653	0.0004336	0.0051564
GO:1901863	BP	GO:1901863	positive r	25/2653	84/17653	0.0004336	0.0051564
GO:0031032	BP	GO:0031032	actomyosin	46/2653	188/17653	0.0004386	0.0052049
GO:0051656	BP	GO:0051656	establishm	94/2653	450/17653	0.000451	0.0053417

GO:0072384	BP	GO:0072384	organelle	23/2653	75/17653	0.0004521	0.0053444
GO:0035601	BP	GO:0035601	protein de	30/2653	108/17653	0.0004596	0.0054123
GO:0007163	BP	GO:0007163	establishm	49/2653	204/17653	0.0004596	0.0054123
GO:0030038	BP	GO:0030038	contractil	26/2653	89/17653	0.0004629	0.0054296
GO:0043149	BP	GO:0043149	stress fib	26/2653	89/17653	0.0004629	0.0054296
GO:0048568	BP	GO:0048568	embryonic	90/2653	428/17653	0.0004707	0.0055103
GO:0044774	BP	GO:0044774	mitotic DN	31/2653	113/17653	0.0004719	0.0055116
GO:0048146	BP	GO:0048146	positive r	18/2653	53/17653	0.0004726	0.0055116
GO:0032386	BP	GO:0032386	regulation	84/2653	395/17653	0.0004963	0.0057623
GO:0035264	BP	GO:0035264	multicellu	37/2653	143/17653	0.0004972	0.0057623
GO:0060351	BP	GO:0060351	cartilage	11/2653	25/17653	0.0004978	0.0057623
GO:0030879	BP	GO:0030879	mammary gl	36/2653	138/17653	0.000498	0.0057623
GO:0048285	BP	GO:0048285	organelle	95/2653	457/17653	0.0005004	0.0057794
GO:0032516	BP	GO:0032516	positive r	9/2653	18/17653	0.0005149	0.0059358
GO:0007254	BP	GO:0007254	JNK cascad	48/2653	200/17653	0.0005324	0.0061256
GO:0098732	BP	GO:0098732	macromolec	30/2653	109/17653	0.0005444	0.0062514
GO:0001782	BP	GO:0001782	B cell hom	12/2653	29/17653	0.0005501	0.0063053
GO:0046834	BP	GO:0046834	lipid phos	31/2653	114/17653	0.0005564	0.00634
GO:0016050	BP	GO:0016050	vesicle or	85/2653	402/17653	0.0005567	0.00634
GO:0018394	BP	GO:0018394	peptidyl-l	40/2653	159/17653	0.0005572	0.00634
GO:1904035	BP	GO:1904035	regulation	23/2653	76/17653	0.0005573	0.00634
GO:0060840	BP	GO:0060840	artery dev	26/2653	90/17653	0.0005592	0.0063497
GO:0006473	BP	GO:0006473	protein ac	46/2653	190/17653	0.0005616	0.0063542
GO:1904036	BP	GO:1904036	negative r	15/2653	41/17653	0.0005641	0.0063542
GO:0035567	BP	GO:0035567	non-canoni	39/2653	154/17653	0.0005642	0.0063542
GO:0006644	BP	GO:0006644	phospholip	97/2653	470/17653	0.0005672	0.0063542
GO:0007596	BP	GO:0007596	blood coag	74/2653	341/17653	0.0005698	0.0063542
GO:0071695	BP	GO:0071695	anatomical	38/2653	149/17653	0.0005698	0.0063542
GO:0030521	BP	GO:0030521	androgen r	21/2653	67/17653	0.0005701	0.0063542
GO:0003206	BP	GO:0003206	cardiac ch	33/2653	124/17653	0.0005718	0.0063542
GO:0000302	BP	GO:0000302	response t	53/2653	227/17653	0.000572	0.0063542
GO:0048592	BP	GO:0048592	eye morpho	37/2653	144/17653	0.0005739	0.0063542
GO:1905332	BP	GO:1905332	positive r	13/2653	33/17653	0.0005747	0.0063542
GO:0030326	BP	GO:0030326	embryonic	34/2653	129/17653	0.0005755	0.0063542
GO:0035113	BP	GO:0035113	embryonic	34/2653	129/17653	0.0005755	0.0063542
GO:0051962	BP	GO:0051962	positive r	101/2653	493/17653	0.0005766	0.0063542
GO:0042770	BP	GO:0042770	signal tra	35/2653	134/17653	0.000577	0.0063542
GO:0061462	BP	GO:0061462	protein lo	14/2653	37/17653	0.0005774	0.0063542
GO:0009895	BP	GO:0009895	negative r	61/2653	270/17653	0.0005776	0.0063542
GO:0050817	BP	GO:0050817	coagulatio	75/2653	347/17653	0.0005935	0.0065177
GO:2000045	BP	GO:2000045	regulation	43/2653	175/17653	0.0006025	0.006605
GO:0003279	BP	GO:0003279	cardiac se	28/2653	100/17653	0.0006091	0.0066642
GO:0010801	BP	GO:0010801	negative r	8/2653	15/17653	0.0006138	0.0066916
GO:1903358	BP	GO:1903358	regulation	8/2653	15/17653	0.0006138	0.0066916
GO:0043543	BP	GO:0043543	protein ac	54/2653	233/17653	0.0006158	0.0067016
GO:0045089	BP	GO:0045089	positive r	76/2653	353/17653	0.0006173	0.0067057
GO:0051188	BP	GO:0051188	cofactor b	66/2653	298/17653	0.0006243	0.0067701
GO:0001667	BP	GO:0001667	ameboidal-	89/2653	426/17653	0.0006269	0.0067856
GO:1901655	BP	GO:1901655	cellular r	25/2653	86/17653	0.0006395	0.0069099
GO:0010971	BP	GO:0010971	positive r	10/2653	22/17653	0.0006558	0.0070732
GO:0006661	BP	GO:0006661	phosphatid	37/2653	145/17653	0.0006609	0.0071157

GO:0036092	BP	GO:0036092	phosphatic	17/2653	50/17653	0.0006635	0.0071301
GO:0043620	BP	GO:0043620	regulation	33/2653	125/17653	0.0006666	0.0071485
GO:0061351	BP	GO:0061351	neural pre	35/2653	135/17653	0.0006683	0.0071485
GO:0014065	BP	GO:0014065	phosphatic	34/2653	130/17653	0.0006687	0.0071485
GO:0010639	BP	GO:0010639	negative r	76/2653	354/17653	0.0006727	0.0071656
GO:0055024	BP	GO:0055024	regulation	26/2653	91/17653	0.0006727	0.0071656
GO:0061333	BP	GO:0061333	renal tubu	23/2653	77/17653	0.0006833	0.0072658
GO:0044706	BP	GO:0044706	multi-mult	51/2653	218/17653	0.0006846	0.0072663
GO:0045017	BP	GO:0045017	glycerolip	61/2653	272/17653	0.0007044	0.007464
GO:1904705	BP	GO:1904705	regulation	19/2653	59/17653	0.0007074	0.0074694
GO:1990874	BP	GO:1990874	vascular s	19/2653	59/17653	0.0007074	0.0074694
GO:0030574	BP	GO:0030574	collagen c	21/2653	68/17653	0.0007096	0.007479
GO:0007093	BP	GO:0007093	mitotic ce	41/2653	166/17653	0.0007133	0.0075052
GO:0090316	BP	GO:0090316	positive r	46/2653	192/17653	0.0007145	0.0075052
GO:0019058	BP	GO:0019058	viral life	69/2653	316/17653	0.0007192	0.0075405
GO:2000278	BP	GO:2000278	regulation	28/2653	101/17653	0.0007237	0.0075754
GO:0032465	BP	GO:0032465	regulation	24/2653	82/17653	0.0007305	0.0076197
GO:0050810	BP	GO:0050810	regulation	24/2653	82/17653	0.0007305	0.0076197
GO:0007565	BP	GO:0007565	female pre	45/2653	187/17653	0.0007347	0.0076506
GO:0046434	BP	GO:0046434	organophos	52/2653	224/17653	0.0007378	0.007669
GO:0007569	BP	GO:0007569	cell aging	30/2653	111/17653	0.0007561	0.0078403
GO:0031952	BP	GO:0031952	regulation	15/2653	42/17653	0.0007568	0.0078403
GO:2000134	BP	GO:2000134	negative r	31/2653	116/17653	0.000766	0.0079025
GO:0044262	BP	GO:0044262	cellular c	59/2653	262/17653	0.0007663	0.0079025
GO:0071236	BP	GO:0071236	cellular r	36/2653	141/17653	0.0007668	0.0079025
GO:1901992	BP	GO:1901992	positive r	22/2653	73/17653	0.0007753	0.0079765
GO:0001706	BP	GO:0001706	endoderm f	18/2653	55/17653	0.0007817	0.0080135
GO:0043388	BP	GO:0043388	positive r	18/2653	55/17653	0.0007817	0.0080135
GO:0006839	BP	GO:0006839	mitochondr	71/2653	328/17653	0.0007829	0.0080135
GO:1902042	BP	GO:1902042	negative r	14/2653	38/17653	0.000789	0.0080625
GO:0060325	BP	GO:0060325	face morph	12/2653	30/17653	0.0007924	0.008073
GO:0035265	BP	GO:0035265	organ grow	42/2653	172/17653	0.0007927	0.008073
GO:2000379	BP	GO:2000379	positive r	26/2653	92/17653	0.0008057	0.0081918
GO:0010038	BP	GO:0010038	response t	72/2653	334/17653	0.0008148	0.0082698
GO:0006338	BP	GO:0006338	chromatin	45/2653	188/17653	0.0008279	0.0083752
GO:1901215	BP	GO:1901215	negative r	45/2653	188/17653	0.0008279	0.0083752
GO:0001570	BP	GO:0001570	vasculogen	23/2653	78/17653	0.0008333	0.0083818
GO:0051492	BP	GO:0051492	regulation	23/2653	78/17653	0.0008333	0.0083818
GO:1905897	BP	GO:1905897	regulation	23/2653	78/17653	0.0008333	0.0083818
GO:0001657	BP	GO:0001657	ureteric b	27/2653	97/17653	0.0008341	0.0083818
GO:0048025	BP	GO:0048025	negative r	9/2653	19/17653	0.0008484	0.0084966
GO:0070734	BP	GO:0070734	histone H3	9/2653	19/17653	0.0008484	0.0084966
GO:0002504	BP	GO:0002504	antigen pr	28/2653	102/17653	0.0008569	0.0085672
GO:0090150	BP	GO:0090150	establishm	65/2653	296/17653	0.0008641	0.0086248
GO:0010811	BP	GO:0010811	positive r	29/2653	107/17653	0.0008742	0.0087121
GO:0036498	BP	GO:0036498	IRE1-media	21/2653	69/17653	0.0008776	0.0087248
GO:0007599	BP	GO:0007599	hemostasis	74/2653	346/17653	0.0008784	0.0087248
GO:0006890	BP	GO:0006890	retrograde	24/2653	83/17653	0.0008835	0.0087318
GO:0045844	BP	GO:0045844	positive r	24/2653	83/17653	0.0008835	0.0087318
GO:0048636	BP	GO:0048636	positive r	24/2653	83/17653	0.0008835	0.0087318
GO:0042254	BP	GO:0042254	ribosome b	72/2653	335/17653	0.0008883	0.0087655

GO:0007173	BP	GO:0007173	epidermal	34/2653	132/17653	0.0008957	0.0088232
GO:2000377	BP	GO:2000377	regulation	42/2653	173/17653	0.0008975	0.0088232
GO:0071333	BP	GO:0071333	cellular r	32/2653	122/17653	0.0008986	0.0088232
GO:0032330	BP	GO:0032330	regulation	16/2653	47/17653	0.0009325	0.009112
GO:0035722	BP	GO:0035722	interleuki	16/2653	47/17653	0.0009325	0.009112
GO:0071349	BP	GO:0071349	cellular r	16/2653	47/17653	0.0009325	0.009112
GO:1905475	BP	GO:1905475	regulation	40/2653	163/17653	0.0009405	0.0091756
GO:0009952	BP	GO:0009952	anterior/p	48/2653	205/17653	0.0009479	0.0092325
GO:0051783	BP	GO:0051783	regulation	44/2653	184/17653	0.0009594	0.0093241
GO:0006475	BP	GO:0006475	internal p	39/2653	158/17653	0.0009604	0.0093241
GO:0035821	BP	GO:0035821	modificati	38/2653	153/17653	0.0009789	0.0094881
GO:0043281	BP	GO:0043281	regulation	50/2653	216/17653	0.0009856	0.0095266
GO:0031346	BP	GO:0031346	positive r	75/2653	353/17653	0.0009891	0.0095266
GO:0019886	BP	GO:0019886	antigen pr	27/2653	98/17653	0.0009892	0.0095266
GO:0042752	BP	GO:0042752	regulation	27/2653	98/17653	0.0009892	0.0095266
GO:0061035	BP	GO:0061035	regulation	20/2653	65/17653	0.0009907	0.0095266
GO:0072215	BP	GO:0072215	regulation	10/2653	23/17653	0.001005	0.0096483
GO:0051384	BP	GO:0051384	response t	36/2653	143/17653	0.0010104	0.0096851
GO:0032956	BP	GO:0032956	regulation	71/2653	331/17653	0.0010157	0.0097197
GO:0071322	BP	GO:0071322	cellular r	34/2653	133/17653	0.0010327	0.0098671
GO:0008654	BP	GO:0008654	phospholip	63/2653	287/17653	0.001038	0.0098989
GO:0002576	BP	GO:0002576	platelet d	33/2653	128/17653	0.0010394	0.0098989
GO:0048706	BP	GO:0048706	embryonic	32/2653	123/17653	0.0010426	0.0098989
GO:1902807	BP	GO:1902807	negative r	32/2653	123/17653	0.0010426	0.0098989
GO:0019080	BP	GO:0019080	viral gene	45/2653	190/17653	0.0010462	0.0099177
GO:0043542	BP	GO:0043542	endothelia	56/2653	249/17653	0.0010602	0.0100338
GO:0003198	BP	GO:0003198	epithelial	8/2653	16/17653	0.0010663	0.0100338
GO:0070875	BP	GO:0070875	positive r	8/2653	16/17653	0.0010663	0.0100338
GO:1905314	BP	GO:1905314	semi-lunar	8/2653	16/17653	0.0010663	0.0100338
GO:0051170	BP	GO:0051170	import int	40/2653	164/17653	0.0010668	0.0100338
GO:0042063	BP	GO:0042063	gliogenesi	58/2653	260/17653	0.0010737	0.0100835
GO:0030258	BP	GO:0030258	lipid modi	62/2653	282/17653	0.0010863	0.0101547
GO:0035459	BP	GO:0035459	cargo load	11/2653	27/17653	0.0010898	0.0101547
GO:0043921	BP	GO:0043921	modulation	11/2653	27/17653	0.0010898	0.0101547
GO:0052472	BP	GO:0052472	modulation	11/2653	27/17653	0.0010898	0.0101547
GO:1900101	BP	GO:1900101	regulation	11/2653	27/17653	0.0010898	0.0101547
GO:2001242	BP	GO:2001242	regulation	39/2653	159/17653	0.0010914	0.0101547
GO:0007015	BP	GO:0007015	actin fila	81/2653	388/17653	0.0010964	0.0101847
GO:0035909	BP	GO:0035909	aorta morp	13/2653	35/17653	0.0011043	0.0102267
GO:1904707	BP	GO:1904707	positive r	13/2653	35/17653	0.0011043	0.0102267
GO:0062012	BP	GO:0062012	regulation	98/2653	485/17653	0.0011066	0.0102328
GO:0071478	BP	GO:0071478	cellular r	43/2653	180/17653	0.001112	0.0102668
GO:2000352	BP	GO:2000352	negative r	12/2653	31/17653	0.0011178	0.0103042
GO:0032970	BP	GO:0032970	regulation	79/2653	377/17653	0.0011208	0.0103165
GO:1903320	BP	GO:1903320	regulation	49/2653	212/17653	0.0011391	0.0104693
GO:0030512	BP	GO:0030512	negative r	22/2653	75/17653	0.0011553	0.0105753
GO:0051702	BP	GO:0051702	interactio	22/2653	75/17653	0.0011553	0.0105753
GO:0051017	BP	GO:0051017	actin fila	36/2653	144/17653	0.0011559	0.0105753
GO:0051249	BP	GO:0051249	regulation	93/2653	457/17653	0.0011605	0.0106011
GO:0007030	BP	GO:0007030	Golgi orga	35/2653	139/17653	0.0011732	0.0107005
GO:0090287	BP	GO:0090287	regulation	60/2653	272/17653	0.0011892	0.0108306

GO:0010463	BP	GO:0010463	mesenchyma	16/2653	48/17653	0.0012061	0.0109371
GO:0043525	BP	GO:0043525	positive r	16/2653	48/17653	0.0012061	0.0109371
GO:0071331	BP	GO:0071331	cellular r	32/2653	124/17653	0.0012064	0.0109371
GO:0043618	BP	GO:0043618	regulation	31/2653	119/17653	0.0012097	0.0109504
GO:0051145	BP	GO:0051145	smooth mus	20/2653	66/17653	0.0012237	0.0110572
GO:0030073	BP	GO:0030073	insulin se	47/2653	202/17653	0.0012251	0.0110572
GO:0031960	BP	GO:0031960	response t	39/2653	160/17653	0.0012379	0.0111558
GO:0016575	BP	GO:0016575	histone de	24/2653	85/17653	0.0012741	0.0114497
GO:0043970	BP	GO:0043970	histone H3	7/2653	13/17653	0.0012762	0.0114497
GO:0070208	BP	GO:0070208	protein he	7/2653	13/17653	0.0012762	0.0114497
GO:1900076	BP	GO:1900076	regulation	21/2653	71/17653	0.0013184	0.0118106
GO:0006309	BP	GO:0006309	apoptotic	9/2653	20/17653	0.001338	0.0119334
GO:2000737	BP	GO:2000737	negative r	9/2653	20/17653	0.001338	0.0119334
GO:2001014	BP	GO:2001014	regulation	9/2653	20/17653	0.001338	0.0119334
GO:2000060	BP	GO:2000060	positive r	26/2653	95/17653	0.001351	0.0120316
GO:0002218	BP	GO:0002218	activation	65/2653	301/17653	0.0013545	0.0120444
GO:0006090	BP	GO:0006090	pyruvate m	34/2653	135/17653	0.0013624	0.0120975
GO:0000375	BP	GO:0000375	RNA splici	70/2653	329/17653	0.0013741	0.0121826
GO:0071326	BP	GO:0071326	cellular r	32/2653	125/17653	0.0013921	0.0122372
GO:0033327	BP	GO:0033327	Leydig cel	6/2653	10/17653	0.0013924	0.0122372
GO:0045955	BP	GO:0045955	negative r	6/2653	10/17653	0.0013924	0.0122372
GO:0061088	BP	GO:0061088	regulation	6/2653	10/17653	0.0013924	0.0122372
GO:1900221	BP	GO:1900221	regulation	6/2653	10/17653	0.0013924	0.0122372
GO:2000586	BP	GO:2000586	regulation	6/2653	10/17653	0.0013924	0.0122372
GO:0061647	BP	GO:0061647	histone H3	14/2653	40/17653	0.0014112	0.0123846
GO:0030217	BP	GO:0030217	T cell dif	51/2653	225/17653	0.0014429	0.0126444
GO:0000956	BP	GO:0000956	nuclear-tr	48/2653	209/17653	0.001465	0.0128191
GO:0032878	BP	GO:0032878	regulation	10/2653	24/17653	0.0014927	0.0129916
GO:0030098	BP	GO:0030098	lymphocyte	70/2653	330/17653	0.0014931	0.0129916
GO:0038202	BP	GO:0038202	TORC1 sign	13/2653	36/17653	0.0014933	0.0129916
GO:0060323	BP	GO:0060323	head morph	13/2653	36/17653	0.0014933	0.0129916
GO:0061383	BP	GO:0061383	trabecula	16/2653	49/17653	0.0015445	0.0133955
GO:0070671	BP	GO:0070671	response t	16/2653	49/17653	0.0015445	0.0133955
GO:0060317	BP	GO:0060317	cardiac ep	12/2653	32/17653	0.0015464	0.0133955
GO:0009108	BP	GO:0009108	coenzyme b	54/2653	242/17653	0.0015514	0.0134185
GO:0052312	BP	GO:0052312	modulation	11/2653	28/17653	0.0015535	0.0134185
GO:0051865	BP	GO:0051865	protein au	18/2653	58/17653	0.0015609	0.0134436
GO:0072665	BP	GO:0072665	protein lo	18/2653	58/17653	0.0015609	0.0134436
GO:0008585	BP	GO:0008585	female gon	26/2653	96/17653	0.0015926	0.0136971
GO:0048199	BP	GO:0048199	vesicle ta	21/2653	72/17653	0.0016019	0.013758
GO:0002495	BP	GO:0002495	antigen pr	27/2653	101/17653	0.0016135	0.0138376
GO:0051897	BP	GO:0051897	positive r	38/2653	157/17653	0.0016262	0.0139266
GO:0072089	BP	GO:0072089	stem cell	29/2653	111/17653	0.0016294	0.013934
GO:0046488	BP	GO:0046488	phosphatid	50/2653	221/17653	0.0016645	0.0142141
GO:0031100	BP	GO:0031100	animal org	22/2653	77/17653	0.0016852	0.0143501
GO:1903845	BP	GO:1903845	negative r	22/2653	77/17653	0.0016852	0.0143501
GO:0032570	BP	GO:0032570	response t	15/2653	45/17653	0.001699	0.0144458
GO:0000377	BP	GO:0000377	RNA splici	69/2653	326/17653	0.0017036	0.0144458
GO:0000398	BP	GO:0000398	mRNA splic	69/2653	326/17653	0.0017036	0.0144458
GO:0000280	BP	GO:0000280	nuclear di	84/2653	411/17653	0.0017178	0.0145461
GO:0035089	BP	GO:0035089	establishm	8/2653	17/17653	0.0017501	0.0147573

GO:0070102	BP	GO:0070102	interleuki	8/2653	17/17653	0.0017501	0.0147573
GO:2000136	BP	GO:2000136	regulation	8/2653	17/17653	0.0017501	0.0147573
GO:0006903	BP	GO:0006903	vesicle ta	24/2653	87/17653	0.0018049	0.0151977
GO:0032102	BP	GO:0032102	negative r	66/2653	310/17653	0.0018183	0.0152896
GO:0010799	BP	GO:0010799	regulation	14/2653	41/17653	0.0018505	0.015495
GO:0044088	BP	GO:0044088	regulation	14/2653	41/17653	0.0018505	0.015495
GO:0047496	BP	GO:0047496	vesicle tr	14/2653	41/17653	0.0018505	0.015495
GO:1905477	BP	GO:1905477	positive r	30/2653	117/17653	0.0018785	0.0157079
GO:0060562	BP	GO:0060562	epithelial	67/2653	316/17653	0.0018811	0.0157079
GO:0000724	BP	GO:0000724	double-str	29/2653	112/17653	0.0018889	0.0157292
GO:0002065	BP	GO:0002065	columnar/c	29/2653	112/17653	0.0018889	0.0157292
GO:0070302	BP	GO:0070302	regulation	49/2653	217/17653	0.00192	0.0159664
GO:0001658	BP	GO:0001658	branching	18/2653	59/17653	0.0019356	0.0159947
GO:0003208	BP	GO:0003208	cardiac ve	21/2653	73/17653	0.0019358	0.0159947
GO:0035019	BP	GO:0035019	somatic st	21/2653	73/17653	0.0019358	0.0159947
GO:0106027	BP	GO:0106027	neuron pro	21/2653	73/17653	0.0019358	0.0159947
GO:0061572	BP	GO:0061572	actin fila	36/2653	148/17653	0.0019367	0.0159947
GO:0046328	BP	GO:0046328	regulation	40/2653	169/17653	0.0019472	0.0160594
GO:0002758	BP	GO:0002758	innate imm	61/2653	283/17653	0.0019539	0.0160701
GO:0060350	BP	GO:0060350	endochondr	16/2653	50/17653	0.0019591	0.0160701
GO:0061971	BP	GO:0061971	replacemen	16/2653	50/17653	0.0019591	0.0160701
GO:2000351	BP	GO:2000351	regulation	16/2653	50/17653	0.0019591	0.0160701
GO:0006310	BP	GO:0006310	DNA recomb	57/2653	261/17653	0.0019751	0.0161568
GO:0051648	BP	GO:0051648	vesicle lo	57/2653	261/17653	0.0019751	0.0161568
GO:2000058	BP	GO:2000058	regulation	35/2653	143/17653	0.0019836	0.0161649
GO:0032881	BP	GO:0032881	regulation	13/2653	37/17653	0.0019894	0.0161649
GO:0042149	BP	GO:0042149	cellular r	13/2653	37/17653	0.0019894	0.0161649
GO:0070423	BP	GO:0070423	nucleotide	13/2653	37/17653	0.0019894	0.0161649
GO:2000785	BP	GO:2000785	regulation	13/2653	37/17653	0.0019894	0.0161649
GO:0007009	BP	GO:0007009	plasma mem	22/2653	78/17653	0.0020198	0.0163678
GO:1900034	BP	GO:1900034	regulation	22/2653	78/17653	0.0020198	0.0163678
GO:0042542	BP	GO:0042542	response t	34/2653	138/17653	0.0020278	0.0163738
GO:0043524	BP	GO:0043524	negative r	34/2653	138/17653	0.0020278	0.0163738
GO:0003272	BP	GO:0003272	endocardia	9/2653	21/17653	0.0020317	0.0163738
GO:0032986	BP	GO:0032986	protein-DN	9/2653	21/17653	0.0020317	0.0163738
GO:0043122	BP	GO:0043122	regulation	50/2653	223/17653	0.0020342	0.0163738
GO:0060968	BP	GO:0060968	regulation	33/2653	133/17653	0.0020687	0.0165876
GO:0007249	BP	GO:0007249	I-kappaB k	56/2653	256/17653	0.002069	0.0165876
GO:1902105	BP	GO:1902105	regulation	56/2653	256/17653	0.002069	0.0165876
GO:0048207	BP	GO:0048207	vesicle ta	19/2653	64/17653	0.0020915	0.0167015
GO:0048208	BP	GO:0048208	COPII vesi	19/2653	64/17653	0.0020915	0.0167015
GO:0060675	BP	GO:0060675	ureteric b	19/2653	64/17653	0.0020915	0.0167015
GO:0010039	BP	GO:0010039	response t	12/2653	33/17653	0.0021017	0.0167165
GO:0010907	BP	GO:0010907	positive r	12/2653	33/17653	0.0021017	0.0167165
GO:0032885	BP	GO:0032885	regulation	12/2653	33/17653	0.0021017	0.0167165
GO:0002573	BP	GO:0002573	myeloid le	44/2653	191/17653	0.0021132	0.0167856
GO:0051196	BP	GO:0051196	regulation	24/2653	88/17653	0.0021345	0.0169325
GO:0051569	BP	GO:0051569	regulation	10/2653	25/17653	0.0021559	0.0170675
GO:0000083	BP	GO:0000083	regulation	11/2653	29/17653	0.0021667	0.0170675
GO:0003203	BP	GO:0003203	endocardia	11/2653	29/17653	0.0021667	0.0170675
GO:1903432	BP	GO:1903432	regulation	11/2653	29/17653	0.0021667	0.0170675

GO:0048704	BP	GO:0048704	embryonic	25/2653	93/17653	0.0021682	0.0170675
GO:0008631	BP	GO:0008631	intrinsic	15/2653	46/17653	0.0021758	0.0170675
GO:0044003	BP	GO:0044003	modificati	15/2653	46/17653	0.0021758	0.0170675
GO:0072132	BP	GO:0072132	mesenchyme	15/2653	46/17653	0.0021758	0.0170675
GO:0000725	BP	GO:0000725	recombinat	29/2653	113/17653	0.0021831	0.0170675
GO:0032922	BP	GO:0032922	circadian	17/2653	55/17653	0.0021876	0.0170675
GO:0046605	BP	GO:0046605	regulation	17/2653	55/17653	0.0021876	0.0170675
GO:0097345	BP	GO:0097345	mitochondr	17/2653	55/17653	0.0021876	0.0170675
GO:0043535	BP	GO:0043535	regulation	36/2653	149/17653	0.0021916	0.0170675
GO:2001020	BP	GO:2001020	regulation	43/2653	186/17653	0.0021944	0.0170675
GO:0019218	BP	GO:0019218	regulation	28/2653	108/17653	0.0021945	0.0170675
GO:0048524	BP	GO:0048524	positive r	27/2653	103/17653	0.0021968	0.0170675
GO:0002683	BP	GO:0002683	negative r	86/2653	426/17653	0.0022092	0.0171133
GO:0050708	BP	GO:0050708	regulation	86/2653	426/17653	0.0022092	0.0171133
GO:0006369	BP	GO:0006369	terminatic	20/2653	69/17653	0.0022214	0.0171133
GO:0060411	BP	GO:0060411	cardiac se	20/2653	69/17653	0.0022214	0.0171133
GO:0007183	BP	GO:0007183	SMAD prote	7/2653	14/17653	0.0022225	0.0171133
GO:0030812	BP	GO:0030812	negative r	7/2653	14/17653	0.0022225	0.0171133
GO:0072216	BP	GO:0072216	positive r	7/2653	14/17653	0.0022225	0.0171133
GO:0002791	BP	GO:0002791	regulation	91/2653	455/17653	0.0022381	0.0172111
GO:0010975	BP	GO:0010975	regulation	90/2653	450/17653	0.0023577	0.0181074
GO:0019083	BP	GO:0019083	viral tran	41/2653	176/17653	0.002361	0.0181103
GO:0045088	BP	GO:0045088	regulation	86/2653	427/17653	0.0023675	0.0181369
GO:1903037	BP	GO:1903037	regulation	64/2653	302/17653	0.002371	0.0181409
GO:0043966	BP	GO:0043966	histone H3	18/2653	60/17653	0.0023831	0.0181639
GO:0045669	BP	GO:0045669	positive r	18/2653	60/17653	0.0023831	0.0181639
GO:1901222	BP	GO:1901222	regulation	18/2653	60/17653	0.0023831	0.0181639
GO:0043124	BP	GO:0043124	negative r	14/2653	42/17653	0.0023973	0.0182489
GO:0090276	BP	GO:0090276	regulation	46/2653	203/17653	0.0024071	0.0182939
GO:0046902	BP	GO:0046902	regulation	22/2653	79/17653	0.0024093	0.0182939
GO:0008286	BP	GO:0008286	insulin re	31/2653	124/17653	0.0024479	0.0185641
GO:0070936	BP	GO:0070936	protein K4	16/2653	51/17653	0.0024627	0.0186528
GO:0090092	BP	GO:0090092	regulation	50/2653	225/17653	0.0024735	0.0186995
GO:0001890	BP	GO:0001890	placenta c	36/2653	150/17653	0.0024751	0.0186995
GO:0032434	BP	GO:0032434	regulation	30/2653	119/17653	0.0024853	0.0187299
GO:1903052	BP	GO:1903052	positive r	30/2653	119/17653	0.0024853	0.0187299
GO:0072009	BP	GO:0072009	nephron ep	28/2653	109/17653	0.0025379	0.0190457
GO:0032006	BP	GO:0032006	regulation	25/2653	94/17653	0.0025402	0.0190457
GO:0006997	BP	GO:0006997	nucleus or	35/2653	145/17653	0.0025452	0.0190457
GO:0071384	BP	GO:0071384	cellular r	19/2653	65/17653	0.0025462	0.0190457
GO:0072171	BP	GO:0072171	mesonephri	19/2653	65/17653	0.0025462	0.0190457
GO:1903313	BP	GO:1903313	positive r	19/2653	65/17653	0.0025462	0.0190457
GO:1901800	BP	GO:1901800	positive r	27/2653	104/17653	0.0025504	0.0190538
GO:0046165	BP	GO:0046165	alcohol bi	34/2653	140/17653	0.0026132	0.0194062
GO:0019048	BP	GO:0019048	modulation	13/2653	38/17653	0.0026137	0.0194062
GO:0031057	BP	GO:0031057	negative r	13/2653	38/17653	0.0026137	0.0194062
GO:0035872	BP	GO:0035872	nucleotide	13/2653	38/17653	0.0026137	0.0194062
GO:2000142	BP	GO:2000142	regulation	13/2653	38/17653	0.0026137	0.0194062
GO:0007088	BP	GO:0007088	regulation	38/2653	161/17653	0.0026179	0.0194135
GO:1905214	BP	GO:1905214	regulation	6/2653	11/17653	0.0026744	0.0197415
GO:1990403	BP	GO:1990403	embryonic	6/2653	11/17653	0.0026744	0.0197415

GO:2000615	BP	GO:2000615	regulation	6/2653	11/17653	0.0026744	0.0197415
GO:0009411	BP	GO:0009411	response t	33/2653	135/17653	0.0026782	0.0197415
GO:0097061	BP	GO:0097061	dendritic	20/2653	70/17653	0.0026785	0.0197415
GO:0046824	BP	GO:0046824	positive r	17/2653	56/17653	0.0027086	0.0199367
GO:0045165	BP	GO:0045165	cell fate	56/2653	259/17653	0.0027116	0.0199367
GO:0010544	BP	GO:0010544	negative r	8/2653	18/17653	0.0027383	0.0199868
GO:0030859	BP	GO:0030859	polarized	8/2653	18/17653	0.0027383	0.0199868
GO:0033194	BP	GO:0033194	response t	8/2653	18/17653	0.0027383	0.0199868
GO:0033599	BP	GO:0033599	regulation	8/2653	18/17653	0.0027383	0.0199868
GO:0051412	BP	GO:0051412	response t	8/2653	18/17653	0.0027383	0.0199868
GO:1903055	BP	GO:1903055	positive r	8/2653	18/17653	0.0027383	0.0199868
GO:0032964	BP	GO:0032964	collagen b	15/2653	47/17653	0.0027586	0.0200143
GO:0035088	BP	GO:0035088	establishm	15/2653	47/17653	0.0027586	0.0200143
GO:0045599	BP	GO:0045599	negative r	15/2653	47/17653	0.0027586	0.0200143
GO:0061245	BP	GO:0061245	establishm	15/2653	47/17653	0.0027586	0.0200143
GO:1905268	BP	GO:1905268	negative r	15/2653	47/17653	0.0027586	0.0200143
GO:0021766	BP	GO:0021766	hippocampu	21/2653	75/17653	0.0027823	0.0201416
GO:0050878	BP	GO:0050878	regulation	98/2653	499/17653	0.0027829	0.0201416
GO:0043254	BP	GO:0043254	regulation	88/2653	441/17653	0.0027973	0.0202218
GO:0006623	BP	GO:0006623	protein ta	12/2653	34/17653	0.0028098	0.0202634
GO:0061384	BP	GO:0061384	heart trab	12/2653	34/17653	0.0028098	0.0202634
GO:0046474	BP	GO:0046474	glyceropho	53/2653	243/17653	0.0028574	0.0205818
GO:0002292	BP	GO:0002292	T cell dif	18/2653	61/17653	0.002914	0.0209398
GO:0071385	BP	GO:0071385	cellular r	18/2653	61/17653	0.002914	0.0209398
GO:0014013	BP	GO:0014013	regulation	27/2653	105/17653	0.0029514	0.0211297
GO:0045446	BP	GO:0045446	endothelia	27/2653	105/17653	0.0029514	0.0211297
GO:0046620	BP	GO:0046620	regulation	27/2653	105/17653	0.0029514	0.0211297
GO:0044068	BP	GO:0044068	modulation	11/2653	30/17653	0.0029623	0.0211297
GO:0070873	BP	GO:0070873	regulation	11/2653	30/17653	0.0029623	0.0211297
GO:0016925	BP	GO:0016925	protein su	25/2653	95/17653	0.0029649	0.0211297
GO:0051983	BP	GO:0051983	regulation	26/2653	100/17653	0.002965	0.0211297
GO:0032872	BP	GO:0032872	regulation	48/2653	216/17653	0.0029776	0.0211921
GO:0051650	BP	GO:0051650	establishm	54/2653	249/17653	0.0029832	0.0211921
GO:0061213	BP	GO:0061213	positive r	9/2653	22/17653	0.0029843	0.0211921
GO:0005979	BP	GO:0005979	regulation	10/2653	26/17653	0.0030369	0.0215152
GO:0010962	BP	GO:0010962	regulation	10/2653	26/17653	0.0030369	0.0215152
GO:0060425	BP	GO:0060425	lung morph	16/2653	52/17653	0.0030692	0.021677
GO:0006270	BP	GO:0006270	DNA replic	14/2653	43/17653	0.0030705	0.021677
GO:1901224	BP	GO:1901224	positive r	14/2653	43/17653	0.0030705	0.021677
GO:0006513	BP	GO:0006513	protein mo	19/2653	66/17653	0.0030808	0.021724
GO:0006650	BP	GO:0006650	glyceropho	74/2653	362/17653	0.0030968	0.0218114
GO:0007162	BP	GO:0007162	negative r	55/2653	255/17653	0.0031077	0.0218627
GO:0002819	BP	GO:0002819	regulation	32/2653	131/17653	0.0031135	0.0218777
GO:0031396	BP	GO:0031396	regulation	42/2653	184/17653	0.0031418	0.0220514
GO:0031349	BP	GO:0031349	positive r	92/2653	466/17653	0.0031464	0.0220576
GO:0007623	BP	GO:0007623	circadian	44/2653	195/17653	0.003208	0.0224123
GO:0030279	BP	GO:0030279	negative r	20/2653	71/17653	0.0032119	0.0224123
GO:0030811	BP	GO:0030811	regulation	20/2653	71/17653	0.0032119	0.0224123
GO:0051851	BP	GO:0051851	modificati	20/2653	71/17653	0.0032119	0.0224123
GO:0046883	BP	GO:0046883	regulation	56/2653	261/17653	0.0032307	0.0225176
GO:0000187	BP	GO:0000187	activation	35/2653	147/17653	0.0032383	0.022519

GO:0006405	BP	GO:0006405	RNA export	35/2653	147/17653	0.0032383	0.022519
GO:0051592	BP	GO:0051592	response t	30/2653	121/17653	0.0032524	0.022566
GO:0043393	BP	GO:0043393	regulation	46/2653	206/17653	0.0032526	0.022566
GO:0000819	BP	GO:0000819	sister chr	52/2653	239/17653	0.0032802	0.0227314
GO:0060627	BP	GO:0060627	regulation	96/2653	490/17653	0.003286	0.0227341
GO:0006732	BP	GO:0006732	coenzyme m	77/2653	380/17653	0.0032881	0.0227341
GO:0031016	BP	GO:0031016	pancreas d	21/2653	76/17653	0.0033105	0.0228627
GO:0010770	BP	GO:0010770	positive r	34/2653	142/17653	0.0033379	0.0230254
GO:0060147	BP	GO:0060147	regulation	28/2653	111/17653	0.0033634	0.0231485
GO:0060966	BP	GO:0060966	regulation	28/2653	111/17653	0.0033634	0.0231485
GO:0033146	BP	GO:0033146	regulation	13/2653	39/17653	0.0033893	0.0232998
GO:0006998	BP	GO:0006998	nuclear en	23/2653	86/17653	0.0034243	0.0234697
GO:0034446	BP	GO:0034446	substrate	23/2653	86/17653	0.0034243	0.0234697
GO:0014066	BP	GO:0014066	regulation	26/2653	101/17653	0.0034335	0.0234697
GO:0046545	BP	GO:0046545	developmen	26/2653	101/17653	0.0034335	0.0234697
GO:0046822	BP	GO:0046822	regulation	26/2653	101/17653	0.0034335	0.0234697
GO:2001243	BP	GO:2001243	negative r	25/2653	96/17653	0.0034481	0.0235429
GO:0098813	BP	GO:0098813	nuclear ch	64/2653	307/17653	0.0035486	0.0242016
GO:0007611	BP	GO:0007611	learning c	52/2653	240/17653	0.0035893	0.024452
GO:0034616	BP	GO:0034616	response t	7/2653	15/17653	0.0036302	0.0245236
GO:0045725	BP	GO:0045725	positive r	7/2653	15/17653	0.0036302	0.0245236
GO:0072075	BP	GO:0072075	metanephri	7/2653	15/17653	0.0036302	0.0245236
GO:1901201	BP	GO:1901201	regulation	7/2653	15/17653	0.0036302	0.0245236
GO:1903798	BP	GO:1903798	regulation	7/2653	15/17653	0.0036302	0.0245236
GO:1904262	BP	GO:1904262	negative r	7/2653	15/17653	0.0036302	0.0245236
GO:0051090	BP	GO:0051090	regulation	83/2653	416/17653	0.003631	0.0245236
GO:0022406	BP	GO:0022406	membrane d	41/2653	180/17653	0.0036324	0.0245236
GO:0022409	BP	GO:0022409	positive r	55/2653	257/17653	0.0036983	0.0249218
GO:2000826	BP	GO:2000826	regulation	12/2653	35/17653	0.0036996	0.0249218
GO:1902903	BP	GO:1902903	regulation	69/2653	336/17653	0.0037261	0.0250722
GO:0010970	BP	GO:0010970	transport	34/2653	143/17653	0.0037603	0.0252462
GO:0099111	BP	GO:0099111	microtubul	34/2653	143/17653	0.0037603	0.0252462
GO:0048844	BP	GO:0048844	artery mor	20/2653	72/17653	0.0038311	0.0256529
GO:0006469	BP	GO:0006469	negative r	56/2653	263/17653	0.0038337	0.0256529
GO:0060828	BP	GO:0060828	regulation	56/2653	263/17653	0.0038337	0.0256529
GO:0045814	BP	GO:0045814	negative r	33/2653	138/17653	0.0038778	0.0259196
GO:0031018	BP	GO:0031018	endocrine	14/2653	44/17653	0.0038909	0.0259781
GO:0030218	BP	GO:0030218	erythrocyt	27/2653	107/17653	0.0039151	0.0260285
GO:0060964	BP	GO:0060964	regulation	27/2653	107/17653	0.0039151	0.0260285
GO:0010001	BP	GO:0010001	glial cell	44/2653	197/17653	0.0039184	0.0260285
GO:0014855	BP	GO:0014855	striated m	21/2653	77/17653	0.00392	0.0260285
GO:0072028	BP	GO:0072028	nephron mo	21/2653	77/17653	0.00392	0.0260285
GO:0051193	BP	GO:0051193	regulation	26/2653	102/17653	0.0039629	0.0262052
GO:1900180	BP	GO:1900180	regulation	26/2653	102/17653	0.0039629	0.0262052
GO:0140014	BP	GO:0140014	mitotic nu	57/2653	269/17653	0.0039668	0.0262052
GO:0044319	BP	GO:0044319	wound heal	11/2653	31/17653	0.0039769	0.0262052
GO:0090505	BP	GO:0090505	epiboly in	11/2653	31/17653	0.0039769	0.0262052
GO:0032436	BP	GO:0032436	positive r	22/2653	82/17653	0.0039771	0.0262052
GO:0072332	BP	GO:0072332	intrinsic	22/2653	82/17653	0.0039771	0.0262052
GO:0048010	BP	GO:0048010	vascular e	25/2653	97/17653	0.0039959	0.0263005
GO:0090559	BP	GO:0090559	regulation	23/2653	87/17653	0.0040063	0.0263399

GO:0060993	BP	GO:0060993	kidney mor	24/2653	92/17653	0.0040114	0.0263449
GO:1905330	BP	GO:1905330	regulation	41/2653	181/17653	0.0040293	0.0264336
GO:0000245	BP	GO:0000245	spliceosom	17/2653	58/17653	0.0040619	0.0265608
GO:0002066	BP	GO:0002066	columnar/c	17/2653	58/17653	0.0040619	0.0265608
GO:0030520	BP	GO:0030520	intracellu	17/2653	58/17653	0.0040619	0.0265608
GO:0006337	BP	GO:0006337	nucleosome	8/2653	19/17653	0.0041127	0.0266907
GO:0032332	BP	GO:0032332	positive r	8/2653	19/17653	0.0041127	0.0266907
GO:0033158	BP	GO:0033158	regulation	8/2653	19/17653	0.0041127	0.0266907
GO:0042762	BP	GO:0042762	regulation	8/2653	19/17653	0.0041127	0.0266907
GO:0061162	BP	GO:0061162	establishm	8/2653	19/17653	0.0041127	0.0266907
GO:0072074	BP	GO:0072074	kidney mes	8/2653	19/17653	0.0041127	0.0266907
GO:0097242	BP	GO:0097242	amyloid-be	8/2653	19/17653	0.0041127	0.0266907
GO:0045665	BP	GO:0045665	negative r	45/2653	203/17653	0.0041315	0.0267834
GO:0042026	BP	GO:0042026	protein re	10/2653	27/17653	0.0041822	0.0270254
GO:0045911	BP	GO:0045911	positive r	10/2653	27/17653	0.0041822	0.0270254
GO:0060251	BP	GO:0060251	regulation	10/2653	27/17653	0.0041822	0.0270254
GO:0002724	BP	GO:0002724	regulation	9/2653	23/17653	0.0042566	0.0272723
GO:0007530	BP	GO:0007530	sex determ	9/2653	23/17653	0.0042566	0.0272723
GO:0010288	BP	GO:0010288	response t	9/2653	23/17653	0.0042566	0.0272723
GO:0043457	BP	GO:0043457	regulation	9/2653	23/17653	0.0042566	0.0272723
GO:0071157	BP	GO:0071157	negative r	9/2653	23/17653	0.0042566	0.0272723
GO:1902895	BP	GO:1902895	positive r	9/2653	23/17653	0.0042566	0.0272723
GO:1902991	BP	GO:1902991	regulation	9/2653	23/17653	0.0042566	0.0272723
GO:2000679	BP	GO:2000679	positive r	9/2653	23/17653	0.0042566	0.0272723
GO:0002260	BP	GO:0002260	lymphocyte	18/2653	63/17653	0.0042729	0.0273472
GO:0051205	BP	GO:0051205	protein in	15/2653	49/17653	0.0043113	0.0275641
GO:0060412	BP	GO:0060412	ventricula	13/2653	40/17653	0.0043417	0.0276443
GO:0070317	BP	GO:0070317	negative r	13/2653	40/17653	0.0043417	0.0276443
GO:1904031	BP	GO:1904031	positive r	13/2653	40/17653	0.0043417	0.0276443
GO:0009612	BP	GO:0009612	response t	46/2653	209/17653	0.0043422	0.0276443
GO:0030177	BP	GO:0030177	positive r	39/2653	171/17653	0.0043748	0.027793
GO:0140056	BP	GO:0140056	organelle	39/2653	171/17653	0.0043748	0.027793
GO:0007052	BP	GO:0007052	mitotic sp	28/2653	113/17653	0.0044057	0.0279599
GO:0006901	BP	GO:0006901	vesicle co	19/2653	68/17653	0.0044324	0.0280702
GO:0060998	BP	GO:0060998	regulation	19/2653	68/17653	0.0044324	0.0280702
GO:0051053	BP	GO:0051053	negative r	32/2653	134/17653	0.0045064	0.0285091
GO:0000018	BP	GO:0000018	regulation	20/2653	73/17653	0.0045462	0.0287009
GO:0072078	BP	GO:0072078	nephron tu	20/2653	73/17653	0.0045462	0.0287009
GO:0008630	BP	GO:0008630	intrinsic	26/2653	103/17653	0.0045592	0.0287528
GO:0006641	BP	GO:0006641	triglyceri	25/2653	98/17653	0.0046149	0.0289494
GO:1903214	BP	GO:1903214	regulation	24/2653	93/17653	0.0046525	0.0289494
GO:0007405	BP	GO:0007405	neuroblast	16/2653	54/17653	0.0046532	0.0289494
GO:1904589	BP	GO:1904589	regulation	16/2653	54/17653	0.0046532	0.0289494
GO:0055001	BP	GO:0055001	muscle cel	40/2653	177/17653	0.0046562	0.0289494
GO:0003177	BP	GO:0003177	pulmonary	6/2653	12/17653	0.004672	0.0289494
GO:0003184	BP	GO:0003184	pulmonary	6/2653	12/17653	0.004672	0.0289494
GO:0016558	BP	GO:0016558	protein im	6/2653	12/17653	0.004672	0.0289494
GO:0032119	BP	GO:0032119	sequesteri	6/2653	12/17653	0.004672	0.0289494
GO:0033160	BP	GO:0033160	positive r	6/2653	12/17653	0.004672	0.0289494
GO:0043201	BP	GO:0043201	response t	6/2653	12/17653	0.004672	0.0289494
GO:0043922	BP	GO:0043922	negative r	6/2653	12/17653	0.004672	0.0289494

GO:0060736	BP	GO:0060736	prostate g	6/2653	12/17653	0.004672	0.0289494
GO:1900102	BP	GO:1900102	negative r	6/2653	12/17653	0.004672	0.0289494
GO:1900363	BP	GO:1900363	regulation	6/2653	12/17653	0.004672	0.0289494
GO:1902993	BP	GO:1902993	positive r	6/2653	12/17653	0.004672	0.0289494
GO:1903729	BP	GO:1903729	regulation	6/2653	12/17653	0.004672	0.0289494
GO:0010976	BP	GO:0010976	positive r	55/2653	260/17653	0.0047649	0.0294655
GO:0010594	BP	GO:0010594	regulation	46/2653	210/17653	0.0047687	0.0294655
GO:0045834	BP	GO:0045834	positive r	30/2653	124/17653	0.0047749	0.0294655
GO:0051291	BP	GO:0051291	protein he	30/2653	124/17653	0.0047749	0.0294655
GO:0045737	BP	GO:0045737	positive r	12/2653	36/17653	0.0048028	0.0295469
GO:0051973	BP	GO:0051973	positive r	12/2653	36/17653	0.0048028	0.0295469
GO:0097352	BP	GO:0097352	autophagos	12/2653	36/17653	0.0048028	0.0295469
GO:0048701	BP	GO:0048701	embryonic	14/2653	45/17653	0.0048808	0.0299964
GO:0071806	BP	GO:0071806	protein tr	17/2653	59/17653	0.0049225	0.0302219
GO:0033673	BP	GO:0033673	negative r	59/2653	283/17653	0.0049571	0.0304029
GO:0001649	BP	GO:0001649	osteoblast	45/2653	205/17653	0.0049967	0.0306148
GO:0031123	BP	GO:0031123	RNA 3'-end	32/2653	135/17653	0.0050744	0.0310595
GO:0051222	BP	GO:0051222	positive r	84/2653	427/17653	0.0050906	0.0311271
GO:0010543	BP	GO:0010543	regulation	11/2653	32/17653	0.0052499	0.032004
GO:0043403	BP	GO:0043403	skeletal m	11/2653	32/17653	0.0052499	0.032004
GO:0090504	BP	GO:0090504	epiboly	11/2653	32/17653	0.0052499	0.032004
GO:0055002	BP	GO:0055002	striated m	37/2653	162/17653	0.0052719	0.0320768
GO:0035914	BP	GO:0035914	skeletal m	19/2653	69/17653	0.0052725	0.0320768
GO:0006096	BP	GO:0006096	glycolytic	25/2653	99/17653	0.0053119	0.0322836
GO:0046634	BP	GO:0046634	regulation	20/2653	74/17653	0.0053683	0.0325935
GO:0050866	BP	GO:0050866	negative r	39/2653	173/17653	0.0053809	0.0326371
GO:0061418	BP	GO:0061418	regulation	21/2653	79/17653	0.0054206	0.0328119
GO:2001022	BP	GO:2001022	positive r	21/2653	79/17653	0.0054206	0.0328119
GO:0021700	BP	GO:0021700	developmen	54/2653	256/17653	0.005443	0.0328816
GO:1901988	BP	GO:1901988	negative r	54/2653	256/17653	0.005443	0.0328816
GO:0010955	BP	GO:0010955	negative r	13/2653	41/17653	0.0054983	0.0331162
GO:0060999	BP	GO:0060999	positive r	13/2653	41/17653	0.0054983	0.0331162
GO:1903318	BP	GO:1903318	negative r	13/2653	41/17653	0.0054983	0.0331162
GO:0031503	BP	GO:0031503	protein-co	52/2653	245/17653	0.0055392	0.0333292
GO:0045727	BP	GO:0045727	positive r	29/2653	120/17653	0.0055564	0.0333995
GO:0015919	BP	GO:0015919	peroxisoma	7/2653	16/17653	0.0056244	0.0335404
GO:0016246	BP	GO:0016246	RNA interf	7/2653	16/17653	0.0056244	0.0335404
GO:0033145	BP	GO:0033145	positive r	7/2653	16/17653	0.0056244	0.0335404
GO:0048070	BP	GO:0048070	regulation	7/2653	16/17653	0.0056244	0.0335404
GO:0051571	BP	GO:0051571	positive r	7/2653	16/17653	0.0056244	0.0335404
GO:0060413	BP	GO:0060413	atrial sep	7/2653	16/17653	0.0056244	0.0335404
GO:0061298	BP	GO:0061298	retina vas	7/2653	16/17653	0.0056244	0.0335404
GO:0080182	BP	GO:0080182	histone H3	7/2653	16/17653	0.0056244	0.0335404
GO:0001702	BP	GO:0001702	gastrulati	10/2653	28/17653	0.0056426	0.0335829
GO:0071634	BP	GO:0071634	regulation	10/2653	28/17653	0.0056426	0.0335829
GO:0008088	BP	GO:0008088	axo-dendri	16/2653	55/17653	0.0056648	0.0336482
GO:0051148	BP	GO:0051148	negative r	16/2653	55/17653	0.0056648	0.0336482
GO:0034101	BP	GO:0034101	erythrocyt	28/2653	115/17653	0.0057067	0.0338636
GO:0090596	BP	GO:0090596	sensory or	53/2653	251/17653	0.0057253	0.0339407
GO:0051153	BP	GO:0051153	regulation	27/2653	110/17653	0.0058482	0.0346353
GO:0031334	BP	GO:0031334	positive r	54/2653	257/17653	0.0059071	0.0348593

GO:0002068	BP	GO:0002068	glandular	9/2653	24/17653	0.005915	0.0348593
GO:0010714	BP	GO:0010714	positive r	9/2653	24/17653	0.005915	0.0348593
GO:0032967	BP	GO:0032967	positive r	9/2653	24/17653	0.005915	0.0348593
GO:0048103	BP	GO:0048103	somatic st	9/2653	24/17653	0.005915	0.0348593
GO:0043297	BP	GO:0043297	apical jun	17/2653	60/17653	0.0059265	0.0348593
GO:0048645	BP	GO:0048645	animal org	17/2653	60/17653	0.0059265	0.0348593
GO:0006109	BP	GO:0006109	regulation	39/2653	174/17653	0.0059531	0.0349351
GO:0003283	BP	GO:0003283	atrial sep	8/2653	20/17653	0.0059625	0.0349351
GO:0061339	BP	GO:0061339	establishm	8/2653	20/17653	0.0059625	0.0349351
GO:2000773	BP	GO:2000773	negative r	8/2653	20/17653	0.0059625	0.0349351
GO:0016574	BP	GO:0016574	histone ub	14/2653	46/17653	0.0060642	0.035462
GO:0045023	BP	GO:0045023	G0 to G1 t	14/2653	46/17653	0.0060642	0.035462
GO:0007389	BP	GO:0007389	pattern sp	83/2653	424/17653	0.0060816	0.0355291
GO:0006757	BP	GO:0006757	ATP genera	25/2653	100/17653	0.0060942	0.0355681
GO:0035794	BP	GO:0035794	positive r	18/2653	65/17653	0.0061146	0.0356528
GO:0034405	BP	GO:0034405	response t	12/2653	37/17653	0.0061532	0.035843
GO:0016049	BP	GO:0016049	cell growt	92/2653	477/17653	0.0061646	0.0358749
GO:0006979	BP	GO:0006979	response t	85/2653	436/17653	0.0061978	0.0360331
GO:0043433	BP	GO:0043433	negative r	38/2653	169/17653	0.0062185	0.0361186
GO:0038034	BP	GO:0038034	signal tra	19/2653	70/17653	0.006239	0.0361332
GO:0061180	BP	GO:0061180	mammary gl	19/2653	70/17653	0.006239	0.0361332
GO:0097192	BP	GO:0097192	extrinsic	19/2653	70/17653	0.006239	0.0361332
GO:0034599	BP	GO:0034599	cellular r	59/2653	286/17653	0.0062563	0.036199
GO:0032231	BP	GO:0032231	regulation	23/2653	90/17653	0.0062675	0.0362002
GO:0050870	BP	GO:0050870	positive r	46/2653	213/17653	0.0062685	0.0362002
GO:0033143	BP	GO:0033143	regulation	20/2653	75/17653	0.0063086	0.0363007
GO:0043470	BP	GO:0043470	regulation	20/2653	75/17653	0.0063086	0.0363007
GO:0072088	BP	GO:0072088	nephron ep	20/2653	75/17653	0.0063086	0.0363007
GO:0097305	BP	GO:0097305	response t	42/2653	191/17653	0.0063137	0.0363007
GO:0019217	BP	GO:0019217	regulation	22/2653	85/17653	0.006316	0.0363007
GO:0046660	BP	GO:0046660	female sex	28/2653	116/17653	0.0064685	0.0371418
GO:0035384	BP	GO:0035384	thioester	15/2653	51/17653	0.0065097	0.0372715
GO:0060997	BP	GO:0060997	dendritic	15/2653	51/17653	0.0065097	0.0372715
GO:0071616	BP	GO:0071616	acyl-CoA b	15/2653	51/17653	0.0065097	0.0372715
GO:0043902	BP	GO:0043902	positive r	39/2653	175/17653	0.0065758	0.0376143
GO:1902850	BP	GO:1902850	microtubul	31/2653	132/17653	0.0066239	0.0378536
GO:1901991	BP	GO:1901991	negative r	50/2653	236/17653	0.0066619	0.0380344
GO:0050931	BP	GO:0050931	pigment ce	11/2653	33/17653	0.0068241	0.038872
GO:0090218	BP	GO:0090218	positive r	11/2653	33/17653	0.0068241	0.038872
GO:0002562	BP	GO:0002562	somatic di	16/2653	56/17653	0.0068472	0.038872
GO:0016444	BP	GO:0016444	somatic ce	16/2653	56/17653	0.0068472	0.038872
GO:0031102	BP	GO:0031102	neuron pro	16/2653	56/17653	0.0068472	0.038872
GO:2001244	BP	GO:2001244	positive r	16/2653	56/17653	0.0068472	0.038872
GO:0006606	BP	GO:0006606	protein im	33/2653	143/17653	0.0068817	0.0390307
GO:0051489	BP	GO:0051489	regulation	13/2653	42/17653	0.0068884	0.0390323
GO:0001938	BP	GO:0001938	positive r	25/2653	101/17653	0.0069694	0.0394542
GO:0097237	BP	GO:0097237	cellular r	49/2653	231/17653	0.0070065	0.0396266
GO:0042255	BP	GO:0042255	ribosome a	17/2653	61/17653	0.0070904	0.0400263
GO:1902110	BP	GO:1902110	positive r	17/2653	61/17653	0.0070904	0.0400263
GO:0009416	BP	GO:0009416	response t	62/2653	305/17653	0.0071997	0.0406053
GO:0070371	BP	GO:0070371	ERK1 and E	66/2653	328/17653	0.0072184	0.0406165

GO:0043154	BP	GO:0043154	negative r	23/2653	91/17653	0.0072219	0.0406165
GO:0046632	BP	GO:0046632	alpha-beta	23/2653	91/17653	0.0072219	0.0406165
GO:0060395	BP	GO:0060395	SMAD prote	18/2653	66/17653	0.0072523	0.0407494
GO:0002042	BP	GO:0002042	cell migra	22/2653	86/17653	0.0073101	0.0410359
GO:0002456	BP	GO:0002456	T cell med	21/2653	81/17653	0.007365	0.0413055
GO:0048565	BP	GO:0048565	digestive	31/2653	133/17653	0.0074235	0.041595
GO:0030199	BP	GO:0030199	collagen f	14/2653	47/17653	0.0074666	0.0417509
GO:1901799	BP	GO:1901799	negative r	14/2653	47/17653	0.0074666	0.0417509
GO:0071604	BP	GO:0071604	transformi	10/2653	29/17653	0.0074721	0.0417509
GO:0014902	BP	GO:0014902	myotube di	27/2653	112/17653	0.0075337	0.0420563
GO:0010510	BP	GO:0010510	regulation	6/2653	13/17653	0.0075828	0.042135
GO:0034497	BP	GO:0034497	protein lc	6/2653	13/17653	0.0075828	0.042135
GO:0035791	BP	GO:0035791	platelet-c	6/2653	13/17653	0.0075828	0.042135
GO:0044794	BP	GO:0044794	positive r	6/2653	13/17653	0.0075828	0.042135
GO:0045198	BP	GO:0045198	establishm	6/2653	13/17653	0.0075828	0.042135
GO:0035329	BP	GO:0035329	hippo sign	12/2653	38/17653	0.0077868	0.0432289
GO:0007159	BP	GO:0007159	leukocyte	67/2653	335/17653	0.0078594	0.0435921
GO:0009166	BP	GO:0009166	nucleotide	37/2653	166/17653	0.0079416	0.0440072
GO:0003158	BP	GO:0003158	endothelit	29/2653	123/17653	0.0079729	0.0440998
GO:2001251	BP	GO:2001251	negative r	29/2653	123/17653	0.0079729	0.0440998
GO:0009648	BP	GO:0009648	photoperic	9/2653	25/17653	0.0080295	0.0442909
GO:0030878	BP	GO:0030878	thyroid gl	9/2653	25/17653	0.0080295	0.0442909
GO:0046856	BP	GO:0046856	phosphatic	9/2653	25/17653	0.0080295	0.0442909
GO:0072577	BP	GO:0072577	endothelia	16/2653	57/17653	0.0082203	0.0452603
GO:1903902	BP	GO:1903902	positive r	16/2653	57/17653	0.0082203	0.0452603
GO:0007062	BP	GO:0007062	sister chr	31/2653	134/17653	0.0083014	0.0455252
GO:0006625	BP	GO:0006625	protein ta	7/2653	17/17653	0.0083363	0.0455252
GO:0035313	BP	GO:0035313	wound heal	7/2653	17/17653	0.0083363	0.0455252
GO:0043923	BP	GO:0043923	positive r	7/2653	17/17653	0.0083363	0.0455252
GO:0061323	BP	GO:0061323	cell proli	7/2653	17/17653	0.0083363	0.0455252
GO:0070816	BP	GO:0070816	phosphoryl	7/2653	17/17653	0.0083363	0.0455252
GO:0070920	BP	GO:0070920	regulation	7/2653	17/17653	0.0083363	0.0455252
GO:0072662	BP	GO:0072662	protein lc	7/2653	17/17653	0.0083363	0.0455252
GO:0072663	BP	GO:0072663	establishm	7/2653	17/17653	0.0083363	0.0455252
GO:0050796	BP	GO:0050796	regulation	38/2653	172/17653	0.0083659	0.0456066
GO:0031498	BP	GO:0031498	chromatin	8/2653	21/17653	0.0083815	0.0456066
GO:0035162	BP	GO:0035162	embryonic	8/2653	21/17653	0.0083815	0.0456066
GO:0043153	BP	GO:0043153	entrainmer	8/2653	21/17653	0.0083815	0.0456066
GO:0043903	BP	GO:0043903	regulation	47/2653	222/17653	0.008433	0.0458452
GO:0022602	BP	GO:0022602	ovulation	13/2653	43/17653	0.0085429	0.0462175
GO:0043631	BP	GO:0043631	RNA polyac	13/2653	43/17653	0.0085429	0.0462175
GO:0071548	BP	GO:0071548	response t	13/2653	43/17653	0.0085429	0.0462175
GO:2000725	BP	GO:2000725	regulation	13/2653	43/17653	0.0085429	0.0462175
GO:0002200	BP	GO:0002200	somatic di	18/2653	67/17653	0.0085551	0.0462175
GO:0051705	BP	GO:0051705	multi-orga	18/2653	67/17653	0.0085551	0.0462175
GO:1905710	BP	GO:1905710	positive r	18/2653	67/17653	0.0085551	0.0462175
GO:0031023	BP	GO:0031023	microtubul	30/2653	129/17653	0.0086239	0.0465057
GO:0043280	BP	GO:0043280	positive r	30/2653	129/17653	0.0086239	0.0465057
GO:0002369	BP	GO:0002369	T cell cyt	11/2653	34/17653	0.008744	0.047027
GO:0097421	BP	GO:0097421	liver rege	11/2653	34/17653	0.008744	0.047027
GO:1904591	BP	GO:1904591	positive r	11/2653	34/17653	0.008744	0.047027

GO:0042866	BP	GO:0042866	pyruvate b	25/2653	103/17653	0.0090309	0.0485273
GO:0042982	BP	GO:0042982	amyloid pr	14/2653	48/17653	0.0091146	0.0489332
GO:1902115	BP	GO:1902115	regulation	36/2653	162/17653	0.0091725	0.0492003
GO:0006457	BP	GO:0006457	protein fo	53/2653	257/17653	0.0092345	0.0493799
GO:0032388	BP	GO:0032388	positive r	50/2653	240/17653	0.0092366	0.0493799
GO:0002224	BP	GO:0002224	toll-like	31/2653	135/17653	0.0092633	0.0493799
GO:0030260	BP	GO:0030260	entry into	31/2653	135/17653	0.0092633	0.0493799
GO:0044409	BP	GO:0044409	entry into	31/2653	135/17653	0.0092633	0.0493799
GO:0051806	BP	GO:0051806	entry into	31/2653	135/17653	0.0092633	0.0493799
GO:0051828	BP	GO:0051828	entry into	31/2653	135/17653	0.0092633	0.0493799
GO:0007098	BP	GO:0007098	centrosome	28/2653	119/17653	0.0092754	0.0494007
GO:0000790	CC	GO:0000790	nuclear ch	103/2754	354/18698	2.07E-12	1.48E-09
GO:0005925	CC	GO:0005925	focal adhe	106/2754	393/18698	1.48E-10	5.29E-08
GO:0005924	CC	GO:0005924	cell-subst	106/2754	396/18698	2.39E-10	5.68E-08
GO:0030055	CC	GO:0030055	cell-subst	106/2754	401/18698	5.19E-10	9.27E-08
GO:0017053	CC	GO:0017053	transcript	35/2754	85/18698	2.82E-09	4.02E-07
GO:0005813	CC	GO:0005813	centrosome	117/2754	490/18698	4.03E-08	4.80E-06
GO:1902911	CC	GO:1902911	protein ki	36/2754	99/18698	8.01E-08	8.17E-06
GO:0005667	CC	GO:0005667	transcript	87/2754	345/18698	1.82E-07	1.62E-05
GO:0016363	CC	GO:0016363	nuclear ma	36/2754	106/18698	5.63E-07	4.42E-05
GO:0031519	CC	GO:0031519	PcG protei	22/2754	50/18698	6.19E-07	4.42E-05
GO:0034399	CC	GO:0034399	nuclear pe	41/2754	131/18698	1.13E-06	7.23E-05
GO:0016442	CC	GO:0016442	RISC compl	9/2754	11/18698	1.34E-06	7.23E-05
GO:0031332	CC	GO:0031332	RNAi effec	9/2754	11/18698	1.34E-06	7.23E-05
GO:0005793	CC	GO:0005793	endoplasmi	39/2754	123/18698	1.42E-06	7.23E-05
GO:1902554	CC	GO:1902554	serine/thr	30/2754	85/18698	1.95E-06	9.26E-05
GO:0098644	CC	GO:0098644	complex of	12/2754	20/18698	4.03E-06	0.0001799
GO:0061695	CC	GO:0061695	transferas	69/2754	277/18698	5.15E-06	0.0002165
GO:0000307	CC	GO:0000307	cyclin-dep	18/2754	41/18698	6.73E-06	0.0002668
GO:0000792	CC	GO:0000792	heterochro	26/2754	73/18698	7.63E-06	0.0002866
GO:0030496	CC	GO:0030496	midbody	46/2754	166/18698	1.05E-05	0.0003763
GO:0098687	CC	GO:0098687	chromosoma	81/2754	347/18698	1.17E-05	0.00039
GO:0031252	CC	GO:0031252	cell leadi	88/2754	385/18698	1.20E-05	0.00039
GO:0030117	CC	GO:0030117	membrane c	31/2754	98/18698	1.73E-05	0.0005114
GO:0048475	CC	GO:0048475	coated mem	31/2754	98/18698	1.73E-05	0.0005114
GO:0000775	CC	GO:0000775	chromosome	51/2754	194/18698	1.79E-05	0.0005114
GO:0016607	CC	GO:0016607	nuclear sp	86/2754	381/18698	2.46E-05	0.0006768
GO:0005819	CC	GO:0005819	spindle	75/2754	324/18698	3.28E-05	0.0008681
GO:0055037	CC	GO:0055037	recycling	44/2754	165/18698	4.51E-05	0.0011509
GO:0036464	CC	GO:0036464	cytoplasmi	49/2754	191/18698	5.12E-05	0.0012601
GO:0031116	CC	GO:0031116	endoplasmi	23/2754	68/18698	6.42E-05	0.0015278
GO:0035770	CC	GO:0035770	ribonuclec	50/2754	200/18698	8.78E-05	0.0020232
GO:0005776	CC	GO:0005776	autophagos	28/2754	92/18698	9.33E-05	0.0020807
GO:0000151	CC	GO:0000151	ubiquitin	70/2754	311/18698	0.0001471	0.0029532
GO:0031093	CC	GO:0031093	platelet a	22/2754	67/18698	0.0001509	0.0029532
GO:0010008	CC	GO:0010008	endosome m	95/2754	451/18698	0.0001541	0.0029532
GO:0005911	CC	GO:0005911	cell-cell	93/2754	440/18698	0.0001585	0.0029532
GO:0008287	CC	GO:0008287	protein se	18/2754	50/18698	0.0001597	0.0029532
GO:1903293	CC	GO:1903293	phosphatas	18/2754	50/18698	0.0001597	0.0029532
GO:0031091	CC	GO:0031091	platelet a	27/2754	90/18698	0.0001613	0.0029532
GO:0044420	CC	GO:0044420	extracellu	33/2754	119/18698	0.0001738	0.0031025

GO:0044440	CC	GO:0044440	endosomal	102/2754	496/18698	0.0002302	0.0040086
GO:0044798	CC	GO:0044798	nuclear tr	46/2754	187/18698	0.0002453	0.0041693
GO:0090575	CC	GO:0090575	RNA polyme	40/2754	157/18698	0.0002795	0.0046168
GO:0000779	CC	GO:0000779	condensed	32/2754	117/18698	0.0002845	0.0046168
GO:0000421	CC	GO:0000421	autophagos	13/2754	32/18698	0.0003297	0.0052314
GO:0000776	CC	GO:0000776	kinetochor	35/2754	133/18698	0.0003399	0.0052442
GO:0005798	CC	GO:0005798	Golgi-assc	43/2754	174/18698	0.0003452	0.0052442
GO:0031965	CC	GO:0031965	nuclear me	67/2754	304/18698	0.0003776	0.0056161
GO:0031256	CC	GO:0031256	leading ec	41/2754	165/18698	0.0004146	0.0060354
GO:0005778	CC	GO:0005778	peroxisoma	19/2754	58/18698	0.0004311	0.0060354
GO:0031903	CC	GO:0031903	microbody	19/2754	58/18698	0.0004311	0.0060354
GO:0035098	CC	GO:0035098	ESC/E(Z) c	9/2754	18/18698	0.0004416	0.0060639
GO:0005938	CC	GO:0005938	cell corte	65/2754	296/18698	0.0005071	0.0068313
GO:0031461	CC	GO:0031461	cullin-RIN	45/2754	188/18698	0.0005388	0.0071247
GO:0005604	CC	GO:0005604	basement m	26/2754	92/18698	0.0005925	0.0073443
GO:0001741	CC	GO:0001741	XY body	7/2754	12/18698	0.0005966	0.0073443
GO:0005583	CC	GO:0005583	fibrillar	7/2754	12/18698	0.0005966	0.0073443
GO:0098643	CC	GO:0098643	banded col	7/2754	12/18698	0.0005966	0.0073443
GO:0000803	CC	GO:0000803	sex chromc	11/2754	26/18698	0.0006267	0.0075847
GO:0030119	CC	GO:0030119	AP-type me	16/2754	47/18698	0.0007461	0.0088784
GO:0000777	CC	GO:0000777	condensed	28/2754	104/18698	0.0008676	0.010027
GO:0000118	CC	GO:0000118	histone de	19/2754	61/18698	0.0008707	0.010027
GO:0030027	CC	GO:0030027	lamellipoc	44/2754	187/18698	0.0009013	0.010215
GO:0032587	CC	GO:0032587	ruffle mem	25/2754	90/18698	0.0009846	0.010984
GO:0005635	CC	GO:0005635	nuclear er	91/2754	455/18698	0.001221	0.0134123
GO:0031588	CC	GO:0031588	nucleotide	6/2754	10/18698	0.0012484	0.0135058
GO:0000791	CC	GO:0000791	euchromati	12/2754	32/18698	0.0012929	0.0137779
GO:0099568	CC	GO:0099568	cytoplasmi	93/2754	468/18698	0.001338	0.0140485
GO:0010494	CC	GO:0010494	cytoplasmi	15/2754	45/18698	0.0013816	0.0141349
GO:0001726	CC	GO:0001726	ruffle	39/2754	164/18698	0.0013858	0.0141349
GO:0120111	CC	GO:0120111	neuron prc	20/2754	68/18698	0.0014297	0.0143776
GO:0030120	CC	GO:0030120	vesicle cc	17/2754	55/18698	0.0017539	0.0171761
GO:0030135	CC	GO:0030135	coated ves	59/2754	276/18698	0.0017561	0.0171761
GO:0000164	CC	GO:0000164	protein ph	7/2754	14/18698	0.0019709	0.0187625
GO:0010369	CC	GO:0010369	chromocent	7/2754	14/18698	0.0019709	0.0187625
GO:0034708	CC	GO:0034708	methyltrar	25/2754	95/18698	0.0022502	0.020894
GO:0030134	CC	GO:0030134	COPII-coat	24/2754	90/18698	0.0022533	0.020894
GO:0030131	CC	GO:0030131	clathrin a	11/2754	30/18698	0.0025175	0.0230449
GO:0005876	CC	GO:0005876	spindle mi	17/2754	57/18698	0.0026852	0.0242688
GO:0005774	CC	GO:0005774	vacuolar m	82/2754	414/18698	0.0027265	0.0243345
GO:0001650	CC	GO:0001650	fibrillar	32/2754	133/18698	0.0029192	0.0257322
GO:0045120	CC	GO:0045120	pronucleus	7/2754	15/18698	0.0032284	0.0281105
GO:0090568	CC	GO:0090568	nuclear tr	11/2754	31/18698	0.0033897	0.0291597
GO:0030175	CC	GO:0030175	filopodium	25/2754	98/18698	0.0035348	0.0292299
GO:0030118	CC	GO:0030118	clathrin c	15/2754	49/18698	0.0035485	0.0292299
GO:0043296	CC	GO:0043296	apical jur	33/2754	140/18698	0.0035871	0.0292299
GO:0032839	CC	GO:0032839	dendrite c	10/2754	27/18698	0.0036021	0.0292299
GO:0035097	CC	GO:0035097	histone me	20/2754	73/18698	0.0036026	0.0292299
GO:0032154	CC	GO:0032154	cleavage f	16/2754	54/18698	0.0038029	0.0305085
GO:0005923	CC	GO:0005923	bicellular	29/2754	121/18698	0.0047285	0.0375124
GO:0070822	CC	GO:0070822	Sin3-type	7/2754	16/18698	0.0050161	0.039357

GO:0005719	CC	GO:0005719	nuclear eu	9/2754	24/18698	0.0051635	0.0400732
GO:0005884	CC	GO:0005884	actin fila	25/2754	101/18698	0.0053872	0.0413595
GO:0005874	CC	GO:0005874	microtubul	79/2754	408/18698	0.0058674	0.0445676
GO:0005788	CC	GO:0005788	endoplasmi	60/2754	297/18698	0.0060711	0.0456292
GO:0005741	CC	GO:0005741	mitochondr	39/2754	178/18698	0.0063232	0.0470289
GO:0030660	CC	GO:0030660	Golgi-asso	27/2754	113/18698	0.0065345	0.048099
GO:0070160	CC	GO:0070160	occluding	29/2754	124/18698	0.006797	0.0489561
GO:1904813	CC	GO:1904813	ficolin-1-	29/2754	124/18698	0.006797	0.0489561
GO:0016580	CC	GO:0016580	Sin3 compl	6/2754	13/18698	0.0068566	0.0489561
GO:0055038	CC	GO:0055038	recycling	19/2754	72/18698	0.0069489	0.0490053
GO:0005769	CC	GO:0005769	early endo	66/2754	334/18698	0.0070008	0.0490053
GO:0000982	MF	GO:0000982	transcript	115/2657	415/17548	2.17E-11	2.46E-08
GO:0000978	MF	GO:0000978	RNA polyme	118/2657	436/17548	6.15E-11	3.48E-08
GO:0000987	MF	GO:0000987	proximal p	120/2657	451/17548	1.37E-10	5.15E-08
GO:0001047	MF	GO:0001047	core promo	52/2657	142/17548	2.34E-10	6.60E-08
GO:0046332	MF	GO:0046332	SMAD bindi	32/2657	74/17548	6.19E-09	1.40E-06
GO:0001228	MF	GO:0001228	transcript	107/2657	417/17548	1.17E-08	2.21E-06
GO:0001076	MF	GO:0001076	transcript	59/2657	191/17548	2.62E-08	4.23E-06
GO:0001227	MF	GO:0001227	transcript	68/2657	233/17548	2.99E-08	4.23E-06
GO:0001046	MF	GO:0001046	core promo	34/2657	87/17548	4.60E-08	5.77E-06
GO:0048407	MF	GO:0048407	platelet-d	10/2657	11/17548	5.92E-08	6.70E-06
GO:0019787	MF	GO:0019787	ubiquitin-	115/2657	472/17548	7.27E-08	7.47E-06
GO:0008013	MF	GO:0008013	beta-cater	32/2657	82/17548	1.18E-07	1.11E-05
GO:0001077	MF	GO:0001077	transcript	75/2657	279/17548	2.50E-07	2.03E-05
GO:0004842	MF	GO:0004842	ubiquitin-	108/2657	446/17548	2.51E-07	2.03E-05
GO:0061659	MF	GO:0061659	ubiquitin-	64/2657	229/17548	4.25E-07	3.20E-05
GO:0044389	MF	GO:0044389	ubiquitin-	83/2657	323/17548	4.70E-07	3.32E-05
GO:0003714	MF	GO:0003714	transcript	63/2657	232/17548	1.53E-06	0.0001019
GO:0003713	MF	GO:0003713	transcript	80/2657	317/17548	1.63E-06	0.0001021
GO:0031625	MF	GO:0031625	ubiquitin	78/2657	309/17548	2.16E-06	0.0001286
GO:0061630	MF	GO:0061630	ubiquitin	60/2657	224/17548	4.33E-06	0.0002449
GO:0001078	MF	GO:0001078	transcript	42/2657	141/17548	7.09E-06	0.0003818
GO:0005161	MF	GO:0005161	platelet-d	10/2657	15/17548	8.98E-06	0.0004613
GO:0070491	MF	GO:0070491	repressing	24/2657	64/17548	9.86E-06	0.0004843
GO:0019888	MF	GO:0019888	protein ph	27/2657	78/17548	1.58E-05	0.000741
GO:0019208	MF	GO:0019208	phosphatas	30/2657	91/17548	1.64E-05	0.000741
GO:0045296	MF	GO:0045296	cadherin b	77/2657	324/17548	2.57E-05	0.0011183
GO:0070888	MF	GO:0070888	E-box binc	16/2657	37/17548	3.89E-05	0.0016287
GO:0016307	MF	GO:0016307	phosphatic	10/2657	17/17548	4.34E-05	0.0017516
GO:0019838	MF	GO:0019838	growth fac	38/2657	133/17548	5.26E-05	0.0020501
GO:0019902	MF	GO:0019902	phosphatas	45/2657	168/17548	6.49E-05	0.0024449
GO:0050839	MF	GO:0050839	cell adhes	105/2657	489/17548	9.59E-05	0.00342
GO:0035257	MF	GO:0035257	nuclear ho	41/2657	151/17548	9.68E-05	0.00342
GO:0042826	MF	GO:0042826	histone de	32/2657	109/17548	0.0001135	0.0038867
GO:0019903	MF	GO:0019903	protein ph	34/2657	119/17548	0.0001272	0.004227
GO:0043021	MF	GO:0043021	ribonucleo	34/2657	120/17548	0.000152	0.0049078
GO:0000400	MF	GO:0000400	four-way j	9/2657	16/17548	0.0001711	0.0053702
GO:0003730	MF	GO:0003730	mRNA 3'-UT	22/2657	66/17548	0.000177	0.0054071
GO:0001104	MF	GO:0001104	RNA polyme	30/2657	103/17548	0.0002136	0.0062619
GO:0070412	MF	GO:0070412	R-SMAD bir	11/2657	23/17548	0.0002161	0.0062619
GO:0035326	MF	GO:0035326	enhancer b	31/2657	108/17548	0.0002258	0.0063798

GO:0070851	MF	GO:0070851	growth fac	36/2657	133/17548	0.0002674	0.0073709
GO:0044325	MF	GO:0044325	ion channe	33/2657	119/17548	0.0002921	0.0078583
GO:0051427	MF	GO:0051427	hormone re	45/2657	179/17548	0.0003146	0.0080205
GO:0005159	MF	GO:0005159	insulin-li	9/2657	17/17548	0.0003147	0.0080205
GO:0033613	MF	GO:0033613	activating	23/2657	73/17548	0.0003265	0.0080205
GO:0043560	MF	GO:0043560	insulin re	7/2657	11/17548	0.0003399	0.0080205
GO:0070411	MF	GO:0070411	I-SMAD bin	7/2657	11/17548	0.0003399	0.0080205
GO:0000217	MF	GO:0000217	DNA second	11/2657	24/17548	0.0003446	0.0080205
GO:0000900	MF	GO:0000900	translatio	8/2657	14/17548	0.0003478	0.0080205
GO:0001190	MF	GO:0001190	transcript	21/2657	65/17548	0.0003999	0.0090375
GO:0001085	MF	GO:0001085	RNA polyme	37/2657	141/17548	0.0004292	0.0095107
GO:0000979	MF	GO:0000979	RNA polyme	14/2657	36/17548	0.0004499	0.0095913
GO:0097472	MF	GO:0097472	cyclin-dep	14/2657	36/17548	0.0004499	0.0095913
GO:0031490	MF	GO:0031490	chromatin	30/2657	108/17548	0.0005222	0.0109277
GO:0035173	MF	GO:0035173	histone ki	9/2657	18/17548	0.0005451	0.0111071
GO:0001158	MF	GO:0001158	enhancer s	27/2657	94/17548	0.0005504	0.0111071
GO:0004712	MF	GO:0004712	protein se	15/2657	41/17548	0.0006117	0.0120491
GO:0043539	MF	GO:0043539	protein se	13/2657	33/17548	0.0006184	0.0120491
GO:0035925	MF	GO:0035925	mRNA 3'-UT	7/2657	12/17548	0.0007091	0.0135804
GO:0003705	MF	GO:0003705	transcript	28/2657	101/17548	0.0008155	0.0153578
GO:1990841	MF	GO:1990841	promoter-s	14/2657	39/17548	0.0011451	0.0207856
GO:0003924	MF	GO:0003924	GTPase act	65/2657	297/17548	0.0011493	0.0207856
GO:0004693	MF	GO:0004693	cyclin-dep	13/2657	35/17548	0.0011857	0.0207856
GO:0030332	MF	GO:0030332	cyclin bin	12/2657	31/17548	0.0011956	0.0207856
GO:0097718	MF	GO:0097718	disordered	12/2657	31/17548	0.0011956	0.0207856
GO:0000287	MF	GO:0000287	magnesium	46/2657	196/17548	0.0013269	0.0224656
GO:0003725	MF	GO:0003725	double-str	20/2657	66/17548	0.0013439	0.0224656
GO:0047485	MF	GO:0047485	protein N-	29/2657	109/17548	0.0013519	0.0224656
GO:0042393	MF	GO:0042393	histone bi	45/2657	194/17548	0.0019054	0.0312048
GO:0001221	MF	GO:0001221	transcript	14/2657	41/17548	0.0019898	0.0321218
GO:0005525	MF	GO:0005525	GTP bindin	77/2657	371/17548	0.0020777	0.032545
GO:0031072	MF	GO:0031072	heat shock	31/2657	122/17548	0.0020978	0.032545
GO:0008757	MF	GO:0008757	S-adenosyl	38/2657	158/17548	0.0021026	0.032545
GO:0036002	MF	GO:0036002	pre-mRNA b	13/2657	37/17548	0.0021313	0.032545
GO:0001205	MF	GO:0001205	transcript	12/2657	33/17548	0.002243	0.0332918
GO:0016538	MF	GO:0016538	cyclin-dep	12/2657	33/17548	0.002243	0.0332918
GO:0030371	MF	GO:0030371	translatio	10/2657	25/17548	0.0022833	0.0332918
GO:0032549	MF	GO:0032549	ribonucleo	78/2657	378/17548	0.002298	0.0332918
GO:0001882	MF	GO:0001882	nucleoside	79/2657	385/17548	0.0025335	0.0362385
GO:0032550	MF	GO:0032550	purine rib	77/2657	374/17548	0.0025981	0.0366976
GO:0061578	MF	GO:0061578	Lys63-spec	6/2657	11/17548	0.0027818	0.038808
GO:0071837	MF	GO:0071837	HMG box do	8/2657	18/17548	0.0028744	0.0396102
GO:0000980	MF	GO:0000980	RNA polyme	21/2657	75/17548	0.0030491	0.0415113
GO:0004843	MF	GO:0004843	thiol-depe	22/2657	80/17548	0.0031422	0.0422706
GO:0001883	MF	GO:0001883	purine nuc	77/2657	377/17548	0.0032292	0.0429299
GO:0016303	MF	GO:0016303	1-phosphat	14/2657	43/17548	0.0032943	0.0432854
GO:0004722	MF	GO:0004722	protein se	20/2657	71/17548	0.003507	0.0450325
GO:0052813	MF	GO:0052813	phosphatid	20/2657	71/17548	0.003507	0.0450325
GO:0035004	MF	GO:0035004	phosphatid	21/2657	76/17548	0.0036237	0.0460088
GO:0004708	MF	GO:0004708	MAP kinase	7/2657	15/17548	0.0037914	0.0476028
GO:0005057	MF	GO:0005057	signal tra	39/2657	169/17548	0.0040249	0.0499797

qvalue	geneID	Count
7.03E-09	HDAC4/EZH2	127
7.03E-09	HDAC4/EZH2	124
4.34E-08	APP/BCL2/V	87
4.34E-08	TGFB3/COL1	106
6.28E-08	HDAC4/COL3	109
6.48E-08	TGFB3/MECP	100
6.48E-08	ARNTL/GSK3	120
6.48E-08	TGFB3/MECP	98
8.24E-08	CDK6/CCND1	77
1.04E-07	HDAC4/FOS/	104
1.91E-07	TGFB3/HDAC	126
2.79E-07	WEE1/CDK6/	85
2.79E-07	CCND1/APP/	102
4.12E-07	BCL2L11/MC	68
4.12E-07	APP/BCL2/V	90
5.09E-07	WEE1/CDK6/	80
5.87E-07	TGFB2/TGFB	63
6.05E-07	TGFB3/MECP	105
6.81E-07	TGFB1/MTOR	46
8.81E-07	EZH2/ARID1	81
1.21E-06	COL7A1/MCF	96
1.38E-06	TGFB3/COL1	102
1.56E-06	TGFB3/LAMC	77
1.61E-06	ARNTL/GSK3	104
1.61E-06	ATM/PTEN/G	102
1.90E-06	TGFB3/CCND	109
1.90E-06	ATM/PTEN/G	90
3.08E-06	COL1A1/CDK	59
3.08E-06	COL1A1/COL	40
3.25E-06	COL1A1/COL	28
3.26E-06	TGFB3/HDAC	48
3.54E-06	TGFB3/MECP	65
3.54E-06	GAPDH/BCL2	72
3.54E-06	CTNNBIP1/B	85
3.54E-06	TGFB3/HDAC	63
3.54E-06	EZH2/ARID1	51
3.61E-06	SP1/FBN1/T	84
3.61E-06	APP/BCL2/T	42
3.61E-06	CCND1/BCL2	108
3.61E-06	TGFB2/MTOR	54
3.61E-06	TGFB3/COL1	46
3.61E-06	TGFB2/TGFB	21
3.72E-06	BCL2/DUSP1	82
3.83E-06	BCL2L11/SP	98
4.35E-06	CCND1/BCL2	107
4.59E-06	TGFB3/HDAC	48
4.63E-06	COL1A1/COL	60
5.63E-06	CCND1/APP/	76
6.04E-06	MTOR/GSK3B	39
8.20E-06	COL5A3/COL	84

8.65E-06	MECP2/DNMT	45
8.65E-06	BCL2L11/SF	78
8.65E-06	MYCN/ESR1/	21
9.18E-06	COL1A1/SP1	105
9.67E-06	TGFB3/ATM/	79
9.67E-06	TGFB3/COL4	66
9.89E-06	TGFB3/BCL2	67
1.02E-05	ARID1A/NCC	49
1.02E-05	TGFB2/TGFE	19
1.02E-05	TGFB3/COL4	65
1.02E-05	MYCN/ESR1/	22
1.02E-05	MYCN/ESR1/	22
1.13E-05	TGFB3/CCND	55
1.13E-05	TGFB3/COL4	79
1.38E-05	CCND1/BCL2	58
1.42E-05	TGFB3/CCND	90
1.56E-05	HDAC4/EZH2	92
1.57E-05	COL1A1/APF	116
1.57E-05	ATXN1/SRSF	89
1.65E-05	LAMC1/BCL2	63
1.70E-05	TGFB3/COL1	60
1.71E-05	ATXN1/SRSF	88
1.71E-05	GAPDH/MCL1	114
1.71E-05	GAPDH/MCL1	114
1.71E-05	MECP2/DNMT	50
1.71E-05	SP1/SPARC/	48
1.71E-05	HDAC4/COL1	80
1.82E-05	APP/SP1/FE	89
1.99E-05	SPARC/PTGS	62
2.04E-05	HDAC4/EZH2	96
2.12E-05	COL1A1/COL	84
2.35E-05	BCL2L11/DN	70
2.42E-05	CTNNBIP1/E	74
2.42E-05	CCND1/BCL2	53
2.44E-05	BCL2L11/ME	33
2.48E-05	SPARC/PTGS	57
2.48E-05	BCL2/VEGFA	31
2.76E-05	EZH2/MECP2	53
2.76E-05	EZH2/MECP2	53
2.76E-05	SRSF10/FMR	39
3.08E-05	COL5A3/CDK	59
3.40E-05	GAPDH/RAB5	76
3.45E-05	COL7A1/MCF	58
3.48E-05	COL4A1/BCL	86
3.67E-05	HDAC4/BCL2	45
3.90E-05	GAPDH/PTEN	48
4.34E-05	VEGFA/ARID	61
4.70E-05	TGFB3/HDAC	80
4.70E-05	MECP2/SFPQ	79
4.70E-05	PTGS2/TGFE	55
4.70E-05	ESR1/PTEN/	75

4.70E-05	BCL2L11/BC	86
4.70E-05	TGFB3/HDAC	73
4.94E-05	SRSF10/FMR	39
5.22E-05	TGFB1/RAF1	54
5.90E-05	HDAC4/COL1	112
5.90E-05	TGFB3/VEGF	47
6.18E-05	APP/BCL2/T	43
6.81E-05	COL4A2/ARI	53
6.81E-05	BCL2/DUSP1	107
6.81E-05	SRSF10/FMR	29
6.81E-05	MYCN/ESR1/	26
7.50E-05	CDK6/CCND1	97
7.51E-05	COL1A1/PTG	66
8.13E-05	CDK6/CCND1	31
8.58E-05	TGFB3/COL1	56
8.69E-05	STMN1/CCDC	12
9.31E-05	COL4A1/VEG	75
9.31E-05	MYCN/TGFB1	13
9.56E-05	ESR1/TGFB1	66
9.56E-05	CDK6/CCND1	32
9.91E-05	TGFB3/EZH2	84
0.0001002	MCL1/BCL2/	58
0.0001122	SP1/PTGS2/	88
0.0001144	BCL2/VEGFA	40
0.000115	CCND1/BCL2	48
0.0001158	SRSF10/MTC	73
0.0001194	HDAC4/BCL2	43
0.0001314	CCND1/BCL2	58
0.0001314	HDAC4/PTGS	30
0.000135	COL1A1/MYC	63
0.000135	ARID1A/ESR	53
0.0001391	SRSF10/ATM	108
0.0001488	CCND1/BCL2	44
0.0001488	CCND1/BCL2	59
0.0001514	CTNNBIP1/E	64
0.0001615	HDAC4/CTNN	84
0.000172	MECP2/DNMT	29
0.0001783	TGFB1/MTOR	21
0.0001784	RAB5A/ATG4	32
0.0001829	MCL1/BCL2/	45
0.0001938	CDK6/EZH2/	62
0.0002033	CCND1/BCL2	41
0.0002109	GAPDH/MCL1	76
0.0002128	CTNNBIP1/E	67
0.0002154	TGFB3/HDAC	58
0.0002236	TGFB3/HDAC	51
0.0002242	HDAC4/BCL2	43
0.0002242	CDK6/BCL2/	34
0.0002242	TGFB2/PTEN	98
0.0002302	RAB5A/ATG4	31
0.0002312	ESR1/PTEN/	75

0.0002316	CCND1/BCL2	41
0.0002318	VEGFA/PTEN	21
0.0002356	BCL2L11/SF	49
0.0002356	VEGFA/ATM/	61
0.0002397	LAMC1/BCL2	30
0.000246	TGFB3/BCL2	76
0.0002534	CCND1/APP/	43
0.0002597	MECP2/DNMT	16
0.0002597	CTNNBIP1/C	82
0.0002597	STMN1/CDC4	48
0.0002607	BCL2L11/BC	59
0.0002639	STMN1/INCE	28
0.0002658	ESR1/TGFB2	12
0.0002658	MYCN/TGFB1	12
0.000266	FMR1/MBNL2	19
0.000266	ATG4D/SH3G	96
0.0002753	MCL1/BCL2/	33
0.0002753	HDAC4/COL3	92
0.0002768	TGFB3/COL1	65
0.0002798	PTEN/CDC42	30
0.0002798	MECP2/DNMT	30
0.0002925	BCL2L11/BC	38
0.0002925	HDAC4/FOS/	17
0.0003027	CCND1/ATM/	45
0.0003078	COL5A3/COL	81
0.0003082	CTNNBIP1/M	53
0.0003082	GAPDH/RAB5	46
0.0003082	CDK6/CCND1	84
0.0003086	ATXN1/SRSF	54
0.0003097	TGFB1/SOX9	28
0.0003112	CCND1/ATM/	47
0.0003112	MECP2/STMN	47
0.0003129	HDAC4/EZH2	69
0.000315	EZH2/MECP2	33
0.000315	CCND1/ATM/	35
0.0003165	COL1A1/CCN	78
0.000336	TGFB3/HDAC	52
0.000336	SP1/VEGFA/	26
0.000352	EZH2/MECP2	39
0.0003721	PTEN/MTOR/	21
0.000389	SRSF10/ATM	53
0.0004323	SRSF10/MTO	26
0.0004412	TGFB3/COL5	37
0.0004412	ARID1A/ESR	40
0.0004449	DNMT3A/BCL	54
0.0004682	STMN1/INCE	24
0.0004682	ATM/HMGA2/	24
0.0004718	CTNNBIP1/C	90
0.0004786	BCL2/WNT4/	48
0.0004848	CTNNBIP1/E	50
0.0004848	BCL2/ATM/F	21

0.0004848	CCND1/BCL2	67
0.0004848	CCND1/SP1/	51
0.0004848	DNMT3B/DNM	57
0.0004848	SRSF10/FMR	52
0.0004848	SRSF10/FMR	52
0.0004868	CTNNBIP1/M	16
0.0004868	EZH2/MECP2	71
0.0004868	COL1A1/COL	44
0.0004907	BCL2/WNT4/	40
0.0004928	SRSF10/CTR	14
0.0005188	VEGFA/TGFB	61
0.0005266	SP1/BCL2L1	33
0.0005563	FGG/FGB/FG	28
0.0005606	MECP2/SFPQ	44
0.0005642	APP/RAVER2	108
0.0005642	COL1A1/CCN	108
0.0005642	HDAC4/CDK6	104
0.0005688	CTNNBIP1/C	30
0.000577	CCND1/APP/	13
0.0005813	APP/VEGFA/	71
0.0005813	ARID1A/ESR	59
0.0005894	CDK6/PTEN/	15
0.0005919	TGFB3/COL1	19
0.0005925	CCND1/DNMT	42
0.0005925	VEGFA/ARID	42
0.0005925	DNMT3A/BCL	52
0.0006169	PTEN/CDC42	82
0.0006371	APP/BCL2L1	81
0.0006491	DICER1/AGO	12
0.0006491	CCND1/ATM/	57
0.0006491	BCL2/VEGFA	25
0.0006491	BCL2/VEGFA	25
0.000656	ATM/HMGA2/	16
0.000656	CTNNBIP1/M	49
0.0006902	EZH2/MECP2	35
0.0007178	TGFB3/TGFB	23
0.0007178	PTEN/GSK3B	58
0.0007523	SP1/PTEN/C	52
0.0007544	HDAC4/COL1	26
0.0007597	SFPQ/FMR1/	21
0.0007597	VEGFA/PTEN	21
0.0007597	SP1/SPARC/	34
0.0007638	TGFB3/COL1	19
0.0007639	MECP2/TGFB	20
0.0007716	TGFB3/COL1	28
0.0007716	CCND1/APP/	28
0.0007716	COL1A1/FOS	59
0.0007716	IFNG/SH3GL	32
0.0007848	FGG/FGB/FG	25
0.0008154	PTEN/GSK3B	54
0.0008204	TGFB3/COL3	48

0.0008204	MECP2/STMN	51
0.0008204	BCL2L11/BC	50
0.0008246	WNT4/NFE2L	36
0.0008246	CCND1/BCL2	66
0.0008259	CDK6/DUSP1	96
0.0008537	COL3A1/COL	101
0.0008537	SRSF10/ATM	58
0.0008537	FGG/FGB/FG	72
0.0008537	CTNNBIP1/M	42
0.000863	CDK6/VEGFA	88
0.0008644	ARID1A/TGF	43
0.0008644	TGFB1/MTOR	16
0.0008644	WNT4/PAFAH	16
0.00087	CDK6/EZH2/	44
0.00087	COL1A1/CCN	74
0.00087	SP1/PTEN/C	62
0.0008813	COL1A1/BCL	53
0.0008815	SH3GLB1/CA	22
0.0008858	PTGS2/TGFE	51
0.0008858	TGFB3/MYCN	50
0.0008881	HMGA2/TGFE	30
0.000907	MECP2/TGFE	21
0.0009181	TGFB2/STMN	20
0.0009181	VEGFA/PTEN	20
0.0009181	VEGFA/PTEN	20
0.0009181	VEGFA/TGFE	20
0.0009181	CCND1/EZH2	80
0.0009359	MTOR/IFNG/	14
0.0009359	CDK6/PTEN/	14
0.0009359	COL1A1/CCN	101
0.0009359	CCND1/BCL2	37
0.0009359	CDK6/CCND1	99
0.0009359	TGFB3/EZH2	66
0.0009359	TGFB3/VEGF	27
0.0009485	CCND1/ATM/	24
0.0009485	CCND1/ATM/	24
0.0009485	COL7A1/MCF	24
0.0009589	SP1/MECP2/	75
0.0009589	COL1A1/MYC	48
0.0009874	TGFB2/TGFE	35
0.0009874	VEGFA/ARID	35
0.0009975	HDAC4/MCL1	57
0.0010265	BCL2/FBN1/	28
0.0010362	AP3M1/VPS4	11
0.0010421	ATM/PPP1R1	65
0.001054	CDC42/RCC2	8
0.001062	HDAC4/FOS/	44
0.0010677	BCL2L11/BC	50
0.0011491	FGG/FGB/FG	20
0.0011698	CCND1/ATM/	24
0.0011761	SRSF10/ATM	53

0.0012005	CDK6/ESR1/	51
0.0012013	FGG/FGB/FG	43
0.0012013	MYCN/ESR1/	30
0.0012013	FOS/COX2/F	58
0.0012013	CDK6/EZH2/	58
0.0012013	CCND1/EZH2	48
0.0012013	VEGFA/PTEN	26
0.0012013	SRSF10/TGF	47
0.0012051	BCL2L11/BC	33
0.0012051	APP/CTR9/D	28
0.0012051	PTGS2/ATM/	37
0.0012136	SP1/MECP2/	75
0.0012371	CCND1/EZH2	38
0.0012815	HDAC4/FOS/	14
0.0012825	SP1/MECP2/	76
0.0012954	MYCN/APP/C	70
0.0012967	EED/KDM5B/	29
0.0012967	EED/KDM5B/	29
0.0013145	TGFB3/VEGF	75
0.0013145	EZH2/MECP2	80
0.0013258	TGFB3/COL1	48
0.0013471	CHUK/TAB3/	21
0.0013471	COL1A1/PTE	21
0.001368	SRSF10/ATM	55
0.0014113	TGFB3/RAC1	20
0.0014486	TGFB3/BCL2	34
0.0014486	MECP2/COL3	77
0.0014486	NCOA3/TGFB	16
0.0014486	VEGFA/TGFB	16
0.001483	SKI/TGFB1/	23
0.0014846	CTNNBIP1/C	97
0.0014897	HDAC4/FOS/	45
0.0014897	GSK3B/NFE2	45
0.0015374	TGFB3/FBN1	32
0.001541	HDAC4/BCL2	25
0.0015488	CDK6/ESR1/	27
0.0015584	MMP24/IGF2	11
0.0015584	BCL2/BFAR/	69
0.0015603	TGFB1/KDM5	13
0.0015604	SRSF10/FMR	58
0.0016071	TGFB3/COL3	76
0.0016071	HDAC4/MYCN	61
0.0016528	FGG/FGB/FG	94
0.0016625	CTNNBIP1/E	28
0.0016625	PTGS2/ATM/	45
0.0016911	SKP2/PAGR1	7
0.0016964	COL4A2/ARI	35
0.0017191	CDK6/CCND1	91
0.0017204	CDC42/BOD1	14
0.0017204	HDAC4/COL1	71
0.0017204	HDAC4/COL1	71

0.001724	CCND1/PTGS	52
0.0017992	APP/ESR1/T	38
0.0017992	CCND1/EZH2	38
0.001812	CDK6/ESR1/	27
0.001812	TGFB3/COL1	27
0.0018136	TGFB3/EZH2	63
0.0018136	COL4A2/LAM	25
0.0018414	CCND1/BCL2	42
0.0018414	APP/SKI/HM	44
0.0018414	AP3M1/BLOC	18
0.0018609	TGFB3/TGFB	34
0.0018609	HDAC4/MTOR	34
0.0018609	BCL2L11/BC	17
0.0018795	GPD1L/DDIT	12
0.0019231	INSIG1/SEC	8
0.0019286	CCND1/ATM/	31
0.0019458	PTEN/GSK3B	50
0.0019493	CDK6/EZH2/	81
0.0019527	CD276/FGG/	85
0.0019558	SP1/MECP2/	63
0.0019959	ATM/BTG2/S	21
0.0019959	TGFB3/FBN1	32
0.0020327	MYCN/BCL2L	45
0.0020327	MYCN/BCL2L	45
0.0020622	MYCN/APP/C	61
0.0020879	MECP2/ESR1	96
0.0020879	FGG/FGB/FG	72
0.0021177	PTEN/ALAD/	13
0.0021177	BCL2L11/BC	13
0.0021177	BCL2L11/BC	13
0.0021254	COL7A1/MCF	25
0.0021439	ATM/MTOR/F	78
0.0021574	ATM/MTOR/F	84
0.0022522	ESR1/TGFB1	53
0.0022733	TGFB2/TGFB	22
0.0022905	TGFB3/MYCN	43
0.0022905	SP1/PTGS2/	43
0.0023006	DNMT3B/DNM	18
0.0023009	TGFB3/CTNN	26
0.0023427	BCL2/CD276	98
0.0023502	DNMT3A/FGG	29
0.0023506	ATM/ARNTL/	17
0.0023506	FZD6/GNB1/	15
0.0023506	BCL2L11/BC	15
0.0024103	WEE1/CDC42	34
0.0024108	ATM/BTG2/S	21
0.0024108	ATM/BTG2/S	21
0.0024299	PTGS2/TGFB	61
0.0025201	FGG/FGB/FG	23
0.0025252	PTEN/GSK3B	37
0.0025473	BCL2L11/BC	43

0.0025499	CCND1/APP/	94
0.0025573	TGFB2/PTEN	41
0.0025649	MECP2/DNMT	20
0.0025649	FGG/FGB/FG	20
0.0026143	HDAC4/CTNN	32
0.0026181	VEGFA/CALM	12
0.0026416	MYCN/APP/C	64
0.0026439	EZH2/DNMT3	76
0.0027252	TGFB3/CTNN	19
0.0027509	RAVER2/SFP	92
0.0027601	TGFB3/HDAC	81
0.002794	MECP2/COL3	70
0.0028205	FGG/FGB/FG	36
0.0028236	HDAC4/CTNN	101
0.0028236	SP1/MECP2/	43
0.0028236	CDK6/BCL2L	37
0.0028236	DNMT3B/TGF	27
0.0028236	RAB5A/ATG4	41
0.0028236	MYCN/BCL2L	39
0.0028236	MYCN/BCL2L	39
0.0028236	SRSF10/FMR	39
0.0028236	APP/TGFB1/	40
0.002877	ATM/BTG2/T	31
0.0028853	ATM/NEK7/H	21
0.0029296	BCL2L11/BC	17
0.0029809	BCL2L11/BC	14
0.002987	EZH2/APP/M	52
0.002991	PTPRJ/HGS/	9
0.0030381	MECP2/COL3	67
0.0030458	BCL2L11/GA	51
0.0030589	TGFB3/BCL2	97
0.0030589	COL1A1/CCN	34
0.0030589	CDK6/RAF1/	34
0.0030812	COL5A3/COL	85
0.0030939	MMP2/RAC1/	26
0.0030939	TGFB3/SKI/	26
0.0031062	SPARC/S10C	98
0.0031062	EZH2/DNMT3	75
0.0031062	RAF1/HMGCR	36
0.0031238	CTNNBIP1/B	37
0.0031307	FGG/FGB/FG	57
0.0031307	TGFB2/TGFB	11
0.0031307	PTPRJ/HGS/	11
0.0031307	SRSF10/DYR	11
0.0031332	HDAC4/SOX9	7
0.0031332	ATM/BTG2/C	24
0.0031332	ATM/BTG2/C	24
0.0031337	MECP2/TGFB	22
0.0031691	DICER1/MRP	8
0.0032234	ATM/MTOR/A	31
0.0032992	BCL2/VEGFA	58

0.0032992	CTNNBIP1/S	32
0.0032992	ESR1/FGG/F	45
0.0033436	EZH2/APP/D	91
0.0033564	BCL2/FGG/F	25
0.0033564	COL7A1/MCF	28
0.0033573	TGFB3/MYCN	43
0.0033573	CTNNBIP1/C	57
0.0033573	ATM/BTG2/S	21
0.0033573	ATM/BTG2/S	21
0.0033573	ATM/BTG2/S	21
0.0033573	APP/CDC42/	42
0.0033573	SFPQ/ATM/D	78
0.0033573	COL3A1/TGF	18
0.0033573	CCND3/CDK4	18
0.0033573	HDAC4/BCL2	18
0.0033779	TGFB2/PTEN	41
0.0034131	CTNNBIP1/E	29
0.0034131	ESR1/PIK3C	29
0.0034131	MYCN/VEGFA	12
0.0034131	CCND1/ESR1	12
0.0034131	ATM/CDKN1A	12
0.0034149	RAF1/HMGCR	52
0.0034149	RAF1/HMGCR	52
0.0034462	MECP2/COL3	77
0.0034526	ATM/HMGA2/	26
0.0034526	TGFB3/STMN	26
0.0034662	TGFB2/STMN	17
0.0034662	TGFB3/COL1	17
0.0034662	WEE1/CDK6/	58
0.0035982	ATM/BTG2/C	24
0.0036135	BCL2L11/BC	13
0.0036743	TGFB2/TGFE	10
0.0036743	RACK1/ROCK	10
0.0036743	SP1/TGFB2/	56
0.0036743	HDAC4/EZH2	42
0.0036743	CD276/TGFE	71
0.0036813	SKI/MTOR/D	14
0.0037284	WEE1/CDK6/	61
0.0037284	APP/VEGFA/	61
0.0037284	HDAC4/SFPQ	28
0.0037284	CTNNBIP1/E	28
0.0037284	CTNNBIP1/E	28
0.0037284	MECP2/NCOA	39
0.0037284	CDK6/CCND1	47
0.0037284	MECP2/NCOA	38
0.0037284	CTNNBIP1/E	36
0.0037284	CDK6/DUSP1	37
0.003747	BCL2L11/BC	25
0.003747	BCL2/TGFB1	25
0.0037822	TGFB3/STMN	46
0.0038816	ATM/RAB5A/	94

0.0038836	CDC42/NEFH	23
0.0039329	HDAC4/SFPQ	30
0.0039329	WEE1/CCL4/	49
0.0039455	TGFB3/STMN	26
0.0039455	TGFB3/STMN	26
0.0040042	TGFB3/MYCN	90
0.0040051	CCND1/ATM/	31
0.0040051	CDK6/ESR1/	18
0.0041873	PTGS2/TGFB	84
0.0041873	APP/BCL2/A	37
0.0041873	COL1A1/SOX	11
0.0041873	TGFB3/CCND	36
0.0041996	BCL2L11/AT	95
0.0043134	MTOR/AGTR2	9
0.0044512	APP/CDC42/	48
0.0045426	HDAC4/SFPQ	30
0.0045818	BCL2L11/BC	12
0.0046071	ESR1/PIK3C	31
0.0046071	BCL2/COL7A	85
0.0046071	MECP2/NCOA	40
0.0046071	FGG/FGB/FG	23
0.0046141	COL3A1/VEG	26
0.0046174	MECP2/NCOA	46
0.0046174	FGG/FGB/FG	15
0.0046174	RAC1/WNT4/	39
0.0046174	MECP2/ESR1	97
0.0046174	COL1A1/COL	74
0.0046174	APP/MECP2/	38
0.0046174	ARID1A/NCO	21
0.0046174	TGFB2/TGFB	33
0.0046174	COL1A1/EZH	53
0.0046174	BCL2/VEGFA	37
0.0046174	VEGFA/TGFB	13
0.0046174	MYCN/BCL2L	34
0.0046174	MYCN/BCL2L	34
0.0046174	EZH2/APP/M	101
0.0046174	ATM/BTG2/T	35
0.0046174	AP3M1/ROCK	14
0.0046174	HDAC4/MCL1	61
0.0047362	COL1A1/COL	75
0.0047996	CDK6/CCND1	43
0.0048426	TGFB2/TGFB	28
0.0048625	DDIT4/CALM	8
0.0048625	MAP2K1/CAM	8
0.0048698	MECP2/NCOA	54
0.0048728	SFPQ/ESR1/	76
0.0049196	HDAC4/GAPD	66
0.0049309	SP1/MECP2/	89
0.0050212	DNMT3B/TGF	25
0.0051399	CCND1/APP/	10
0.0051707	ATM/PTEN/R	37

0.0051812	ATM/PIK3CG	17
0.0051946	VEGFA/NFE2	33
0.0051946	VEGFA/TGFE	35
0.0051946	TGFB2/PTEN	34
0.005207	MECP2/DNMT	76
0.005207	TGFB1/PTEN	26
0.0052798	CTNNBIP1/C	23
0.0052802	TGFB3/FOS/	51
0.0054238	ATM/PTEN/F	61
0.0054278	TGFB3/MMP2	19
0.0054278	TGFB3/MMP2	19
0.0054347	COL5A3/COL	21
0.0054537	CCND1/ATM/	41
0.0054537	PTGS2/TGFE	46
0.0054794	BCL2/HMGA2	69
0.0055048	ATM/DUSP1/	28
0.005537	CDC42/SH3G	24
0.005537	SP1/WNT4/I	24
0.0055594	TGFB3/FOS/	45
0.0055728	HDAC4/TDG/	52
0.0056972	CDK6/BCL2/	30
0.0056972	VEGFA/JUN/	15
0.0057425	CCND1/EZH2	31
0.0057425	HDAC4/MECF	59
0.0057425	EZH2/DNMT3	36
0.0057962	CCND1/APP/	22
0.0058231	COL4A2/MMF	18
0.0058231	SKI/HMGA2/	18
0.0058231	BCL2L11/BC	71
0.0058587	FGG/FGB/FG	14
0.0058664	TGFB3/COL1	12
0.0058664	BCL2L11/BC	42
0.0059527	HDAC4/PTGS	26
0.0060093	CCND1/APP/	72
0.006086	HDAC4/ARID	45
0.006086	TGFB3/MECF	45
0.0060908	VEGFA/TGFE	23
0.0060908	TGFB3/STMN	23
0.0060908	BCL2L11/NF	23
0.0060908	CTNNBIP1/E	27
0.0061742	SRSF10/DYF	9
0.0061742	EZH2/SUZ12	9
0.0062254	DCTN4/AP1S	28
0.0062673	BCL2L11/BC	65
0.0063307	CDK6/VEGFA	29
0.00634	BCL2L11/DN	21
0.00634	COL1A1/COL	74
0.0063451	ARF4/KIF2C	24
0.0063451	BCL2/TGFB1	24
0.0063451	BCL2/TGFB1	24
0.0063696	PTEN/DDX21	72

0.0064115	APP/TGFB1/	34
0.0064115	HDAC4/MYCN	42
0.0064115	RAF1/HMGCR	32
0.0066214	RUNX2/SOX9	16
0.0066214	CDC42/IFNG	16
0.0066214	CDC42/IFNG	16
0.0066675	BCL2L11/BC	40
0.0067089	CTNNBIP1/S	48
0.0067755	ATM/DUSP1/	44
0.0067755	MECP2/NCOA	39
0.0068947	SP1/BCL2L1	38
0.0069226	BCL2L11/VE	50
0.0069226	TGFB3/HDAC	75
0.0069226	DCTN4/AP1S	27
0.0069226	EZH2/SFPQ/	27
0.0069226	TGFB1/BMP1	20
0.0070111	AGTR2/PDGF	10
0.0070378	CCND1/BCL2	36
0.0070629	TGFB3/TGFB	71
0.00717	RAF1/HMGCR	34
0.0071932	ATM/PTEN/R	63
0.0071932	TGFB3/APP/	33
0.0071932	TGFB3/COL1	32
0.0071932	CCND1/EZH2	32
0.0072068	SP1/CCL4/H	45
0.0072912	SP1/MECP2/	56
0.0072912	TGFB2/TGFB	8
0.0072912	AKT2/INSR/	8
0.0072912	TGFB2/NOTC	8
0.0072912	PTGS2/TGFB	40
0.0073273	CDK6/MYCN/	58
0.0073791	ESR1/PTEN/	62
0.0073791	INSIG1/SEC	11
0.0073791	SP1/CCL4/H	11
0.0073791	SP1/CCL4/H	11
0.0073791	BCL2L11/BF	11
0.0073791	BCL2L11/SF	39
0.0074009	TGFB3/BCL2	81
0.0074314	COL3A1/TGF	13
0.0074314	MMP2/JUN/M	13
0.0074358	HDAC4/SP1/	98
0.0074605	PTGS2/ATM/	43
0.0074877	FGG/FGB/FG	12
0.0074966	TGFB3/TGFB	79
0.0076077	HDAC4/PTEN	49
0.0076847	TGFB3/FBN1	22
0.0076847	SP1/GAPDH/	22
0.0076847	TGFB3/STMN	36
0.0077034	BCL2/CD276	93
0.0077757	CDC42/RAB3	35
0.0078702	TGFB3/VEGF	60

0.0079476	CTNNBIP1/M	16
0.0079476	HDAC4/BCL2	16
0.0079476	RAF1/HMGCR	32
0.0079573	VEGFA/NFE2	31
0.0080349	MECP2/VEGF	20
0.0080349	RAF1/ARNTL	47
0.0081065	COL1A1/CCN	39
0.0083201	HDAC4/SFPQ	24
0.0083201	NAP1L2/CHE	7
0.0083201	COL1A1/COL	7
0.0085823	CCND3/CDK4	21
0.0086715	DICER1/CEC	9
0.0086715	NFE2L2/RES	9
0.0086715	S100B/ARNT	9
0.0087429	PTEN/GSK3E	26
0.0087522	SFPQ/ESR1/	65
0.0087908	HDAC4/GAPD	34
0.0088526	RAVER2/SFF	70
0.0088923	RAF1/HMGCR	32
0.0088923	CCND1/PLEK	6
0.0088923	FMR1/SYT4/	6
0.0088923	SLC30A5/SL	6
0.0088923	LDLR/PICAL	6
0.0088923	PDGFRB/PDG	6
0.0089994	MECP2/DNMT	14
0.0091882	BCL2/TGFB1	51
0.0093151	ATM/SECISE	48
0.0094405	RACK1/ROCK	10
0.0094405	HDAC4/BCL2	70
0.0094405	ATM/MTOR/C	13
0.0094405	TGFB3/COL1	13
0.009734	COL1A1/FBN	16
0.009734	CDC42/IFNG	16
0.009734	TGFB2/TGFE	12
0.0097508	HDAC4/GAPD	54
0.0097508	SP1/CCL4/H	11
0.009769	BFAR/UBE2E	18
0.009769	SH3GLB1/AF	18
0.0099532	BCL2/VEGFA	26
0.0099974	COL7A1/MCF	21
0.0100553	DCTN4/AP1S	27
0.0101199	ESR1/TGFB1	38
0.0101254	VEGFA/HMGA	29
0.0103289	ESR1/ATM/F	50
0.0104277	CCND1/EZH2	22
0.0104277	TGFB3/FBN1	22
0.0104972	TGFB3/FOS/	15
0.0104972	RAVER2/SFF	69
0.0104972	RAVER2/SFF	69
0.0105701	BCL2L11/AT	84
0.0107236	CDC42/MYO9	8

0.0107236	IL6/CTR9/J	8
0.0107236	SOX9/PIM1/	8
0.0110436	COL7A1/MCF	24
0.0111104	FGG/FGB/FG	66
0.0112597	APP/TGFB1/	14
0.0112597	RAB5A/SH3G	14
0.0112597	AP3M1/BLOC	14
0.0114144	BCL2L11/BC	30
0.0114144	CTNNBIP1/M	67
0.0114298	SFPQ/ATM/M	29
0.0114298	CDK6/MYCN/	29
0.0116022	APP/VEGFA/	49
0.0116228	CTNNBIP1/E	18
0.0116228	TGFB2/TGFE	21
0.0116228	SKI/RAF1/S	21
0.0116228	APP/PTEN/C	21
0.0116228	TGFB3/STMN	36
0.0116698	APP/CDC42/	40
0.0116776	ESR1/FGG/F	61
0.0116776	COL1A1/RUN	16
0.0116776	COL1A1/RUN	16
0.0116776	FGG/FGB/FG	16
0.0117406	SFPQ/ATM/T	57
0.0117406	PTEN/RAB5A	57
0.0117464	PTEN/GSK3E	35
0.0117464	TGFB1/MTOR	13
0.0117464	BCL2/NFE2L	13
0.0117464	CHUK/TAB3/	13
0.0117464	RAB5A/SH3G	13
0.0118939	TGFB2/PTEN	22
0.0118939	ATM/MTOR/G	22
0.0118982	COL1A1/EZH	34
0.0118982	TGFB3/MECF	34
0.0118982	TGFB2/TGFE	9
0.0118982	ARID1A/SMA	9
0.0118982	S100B/ESR1	50
0.0120536	MYCN/DNMT1	33
0.0120536	S100B/ESR1	56
0.0120536	CTNNBIP1/C	56
0.0121364	COL7A1/MCF	19
0.0121364	COL7A1/MCF	19
0.0121364	CTNNBIP1/E	19
0.0121473	CCND1/BCL2	12
0.0121473	AKT2/PMAIF	12
0.0121473	TGFB1/MTOR	12
0.0121975	CTNNBIP1/C	44
0.0123042	HDAC4/PDHX	24
0.0124023	DNMT3B/DNM	10
0.0124023	CCNE1/CDC6	11
0.0124023	TGFB2/SOX9	11
0.0124023	ATM/C9orf7	11

0.0124023	TGFB3/MYCN	25
0.0124023	SFPQ/MCL1/	15
0.0124023	BCL2L11/GA	15
0.0124023	TGFB2/SOX9	15
0.0124023	SFPQ/ATM/M	29
0.0124023	ARNTL/CLOC	17
0.0124023	CHEK1/ROCK	17
0.0124023	BCL2L11/BC	17
0.0124023	SP1/MECP2/	36
0.0124023	MCL1/ATM/H	43
0.0124023	SP1/WNT4/I	28
0.0124023	SP1/FMR1/J	27
0.0124356	TGFB3/CDK6	86
0.0124356	TGFB3/GAPD	86
0.0124356	DDX39A/EIF	20
0.0124356	TGFB2/TGFB	20
0.0124356	TGFB1/SMAD	7
0.0124356	HDAC4/DDIT	7
0.0124356	AGTR2/PDGF	7
0.0125067	TGFB3/GAPD	91
0.013158	EZH2/VEGFA	90
0.0131601	SP1/CCL4/H	41
0.0131794	SFPQ/ESR1/	86
0.0131823	CD276/TGFB	64
0.013199	NAP1L2/CHE	18
0.013199	CTNNBIP1/F	18
0.013199	APP/TSPAN6	18
0.0132608	ESR1/TNIP3	14
0.0132935	FGG/FGB/FG	46
0.0132935	BCL2L11/BC	22
0.0134899	CCND3/CDK4	31
0.0135543	BFAR/UBE2E	16
0.0135882	TGFB3/FBN1	50
0.0135882	ARID1A/PTG	36
0.0136103	GSK3B/NFE2	30
0.0136103	PTEN/GSK3B	30
0.0138398	CTNNBIP1/E	28
0.0138398	ATM/ARNTL/	25
0.0138398	NSFL1C/TOR	35
0.0138398	BCL2L11/DN	19
0.0138398	CTNNBIP1/E	19
0.0138398	BTG2/TNRC6	19
0.0138457	GSK3B/NFE2	27
0.0141018	SP1/IFNG/H	34
0.0141018	BCL2L11/TG	13
0.0141018	MECP2/DNMT	13
0.0141018	CHUK/TAB3/	13
0.0141018	CTNNBIP1/E	13
0.0141071	ATM/DUSP1/	38
0.0143454	FMR1/EIF4A	6
0.0143454	RPL10/EN2/	6

0.0143454	NAP1L2/CHE	6
0.0143454	CCND1/BCL2	33
0.0143454	PTEN/CDC42	20
0.0144872	PTGS2/TGFE	17
0.0144872	MCL1/BCL2/	56
0.0145236	FGG/PRKCD/	8
0.0145236	CDC42/MYO9	8
0.0145236	CHUK/JAK2/	8
0.0145236	CCND1/KDM5	8
0.0145236	CCND1/FOS/	8
0.0145236	TGFB1/SOX9	8
0.0145437	TGFB3/COL1	15
0.0145437	CDC42/ARF4	15
0.0145437	TGFB1/ARNT	15
0.0145437	CDC42/ARF4	15
0.0145437	MECP2/DNMT	15
0.0146362	CDK6/EZH2/	21
0.0146362	COL1A1/CCN	98
0.0146945	CTNNBIP1/E	88
0.0147247	AP3M1/VPS4	12
0.0147247	TGFB2/TGFE	12
0.0149561	ATM/PTEN/F	53
0.0152162	IL6/TBX21/	18
0.0152162	BCL2L11/DN	18
0.0153542	MYCN/EZH2/	27
0.0153542	CLIC4/NR2F	27
0.0153542	BCL2L11/PT	27
0.0153542	BCL2L11/GA	11
0.0153542	MTOR/GSK3E	11
0.0153542	HDAC4/BCL1	25
0.0153542	SFPQ/ATM/D	26
0.0153995	APP/VEGFA/	48
0.0153995	RAB5A/COL7	54
0.0153995	VEGFA/TGFE	9
0.0156343	MTOR/GSK3E	10
0.0156343	MTOR/GSK3E	10
0.0157519	CDC42/SOX9	16
0.0157519	CCNE1/CDC6	14
0.0157519	APP/AGER/A	14
0.015786	UBE2W/UBE2	19
0.0158496	MECP2/ESR1	74
0.0158868	COL1A1/FGG	55
0.0158977	TGFB1/IL6/	32
0.0160239	PTEN/MTOR/	42
0.0160284	SFPQ/PTGS2	92
0.0162862	EZH2/SFPQ/	44
0.0162862	HDAC4/CDK6	20
0.0162862	HDAC4/NUPL	20
0.0162862	SP1/GAPDH/	20
0.0163627	FGG/FGB/FG	56
0.0163637	TGFB3/TAB3	35

0.0163637	SRSF10/NUF	35
0.0163979	CCND1/FOS/	30
0.0163979	HDAC4/CTNN	46
0.0165181	SFPQ/ATM/D	52
0.01652	VEGFA/FGG/	96
0.01652	HDAC4/GAPD	77
0.0166135	CDK6/ARNTL	21
0.0167317	VEGFA/FGG/	34
0.0168212	MYCN/ESR1/	28
0.0168212	MYCN/ESR1/	28
0.0169311	ESR1/SKP2/	13
0.0170546	NSFL1C/TOR	23
0.0170546	LAMC1/FGG/	23
0.0170546	TGFB2/PTEN	26
0.0170546	BCL2/VEGFA	26
0.0170546	PTGS2/TGFB	26
0.0171078	MCL1/BCL2/	25
0.0175864	SFPQ/ATM/D	64
0.0177684	ATXN1/APP/	52
0.0178204	TGFB3/TGFB	7
0.0178204	AKT2/INSR/	7
0.0178204	WNT4/SMAD4	7
0.0178204	TGFB1/SOX9	7
0.0178204	MYCN/ESR1/	7
0.0178204	ATM/BMT2/S	7
0.0178204	HDAC4/CTNN	83
0.0178204	RAB8B/BLOC	41
0.0181098	CD276/FGG/	55
0.0181098	TGFB2/SOX9	12
0.0182191	TGFB3/APP/	69
0.0183455	APP/CDC42/	34
0.0183455	APP/CDC42/	34
0.018641	COL3A1/VEG	20
0.018641	CDK6/DUSP1	56
0.018641	COL1A1/WNT	56
0.0188348	EZH2/MECP2	33
0.0188774	CDK6/ARNTL	14
0.0189139	CDK6/VEGFA	27
0.0189139	MYCN/ESR1/	27
0.0189139	CDK6/MYCN/	44
0.0189139	TGFB2/PTEN	21
0.0189139	CTNNBIP1/E	21
0.0190423	HDAC4/RAC1	26
0.0190423	PTGS2/TGFB	26
0.0190423	ATM/DUSP1/	57
0.0190423	PTEN/MTOR/	11
0.0190423	PTEN/MTOR/	11
0.0190423	GSK3B/NFE2	22
0.0190423	PPP1R13B/F	22
0.0191116	VEGFA/RAC1	25
0.0191403	BCL2L11/BC	23

0.0191439	CTNNBIP1/E	24
0.0192083	VEGFA/ESR1	41
0.0193008	SRSF10/SCA	17
0.0193008	CDK6/ARNTL	17
0.0193008	ARID1A/ESR	17
0.0193952	ARID1A/SMA	8
0.0193952	RUNX2/SOX9	8
0.0193952	IFNG/JAK2/	8
0.0193952	SP1/NFE2L2	8
0.0193952	CDC42/MYO9	8
0.0193952	WNT4/AMER1	8
0.0193952	IGF1R/LDLR	8
0.0194625	APP/PTEN/C	45
0.0196384	B2M/HSPA1E	10
0.0196384	TGFB1/TBX2	10
0.0196384	SKI/MTOR/D	10
0.0198178	IL6/TBX21/	9
0.0198178	WNT4/SOX9/	9
0.0198178	APP/SPARC/	9
0.0198178	CISD1/CDK1	9
0.0198178	CCND1/CDK4	9
0.0198178	FOS/TGFB2/	9
0.0198178	APP/ROCK2/	9
0.0198178	HMGA2/TGFE	9
0.0198722	BCL2L11/BC	18
0.0200298	BCL2L11/BC	15
0.0200881	TGFB2/TGFE	13
0.0200881	EZH2/SUZ12	13
0.0200881	CCND1/CCND	13
0.0200881	HDAC4/COL1	46
0.0201962	COL1A1/SKI	39
0.0201962	RAB8B/BLOC	39
0.0203174	MECP2/STMN	28
0.0203976	COL7A1/MCF	19
0.0203976	PTEN/MTOR/	19
0.0207165	TGFB3/TET1	32
0.0208559	TGFB1/TBX2	20
0.0208559	CTNNBIP1/E	20
0.0208936	BCL2L11/MC	26
0.0210364	PIK3CG/INS	25
0.0210364	SH3GLB1/UE	24
0.0210364	VEGFA/TGFE	16
0.0210364	PTGS2/TGFE	16
0.0210364	HDAC4/BCL2	40
0.0210364	TGFB2/NOTC	6
0.0210364	TGFB2/NOTC	6
0.0210364	PEX13/PEX2	6
0.0210364	SLC30A5/SL	6
0.0210364	IFNG/JAK2/	6
0.0210364	MTOR/RPS6K	6
0.0210364	CCL4/HMGA2	6

0.0210364	ESR1/PTEN/	6
0.0210364	BFAR/RACK1	6
0.0210364	CTR9/RNF2C	6
0.0210364	APP/ROCK2/	6
0.0210364	TGFB2/SH3G	6
0.0214115	EZH2/VEGFA	55
0.0214115	SP1/MECP2/	46
0.0214115	PTGS2/TGFB	30
0.0214115	COL1A1/COL	30
0.0214706	CCND1/CCND	12
0.0214706	NEK7/HMBOX	12
0.0214706	CALCOCO2/A	12
0.0217973	TGFB3/RUNX	14
0.0219611	MCL1/TIMM4	17
0.0220927	CDK6/DUSP1	59
0.0222466	HDAC4/CTNN	45
0.0225698	APP/CTR9/D	32
0.0226189	TGFB3/GAPD	84
0.0232561	FGG/PRKCD/	11
0.0232561	EZH2/BCL9/	11
0.0232561	PTEN/MTOR/	11
0.0233091	HDAC4/BCL2	37
0.0233091	FOS/S100B/	19
0.0234593	HDAC4/GAPD	25
0.0236845	TBX21/IFNG	20
0.0237162	FGG/ATM/TG	39
0.0238432	VEGFA/NFE2	21
0.0238432	ATM/HMGA2/	21
0.0238939	APP/MECP2/	54
0.0238939	CCND1/EZH2	54
0.0240643	C4BPB/XIAP	13
0.0240643	MTOR/FMR1/	13
0.0240643	C4BPB/XIAP	13
0.0242191	SRSF10/ATM	52
0.0242702	ESR1/TGFB1	29
0.0243726	RAB8B/PEX1	7
0.0243726	DICER1/MRP	7
0.0243726	SKP2/PAGR1	7
0.0243726	BCL2L11/BC	7
0.0243726	DNMT3B/DNM	7
0.0243726	TGFB2/NOTC	7
0.0243726	COL4A1/CLI	7
0.0243726	CTR9/HIST1	7
0.0244035	ACVR2B/FRS	10
0.0244035	PTGS2/TGFB	10
0.0244509	APP/FMR1/A	16
0.0244509	HDAC4/EZH2	16
0.0246075	CDK6/VEGFA	28
0.0246635	BCL2/VEGFA	53
0.0251682	HDAC4/EZH2	27
0.025331	BCL2L11/ME	54

0.025331	CDK6/ARNTL	9
0.025331	TGFB3/TGFE	9
0.025331	TGFB3/TGFE	9
0.025331	TGFB2/NUME	9
0.025331	CLDN1/ROCK	17
0.025331	MAP2K1/FRS	17
0.025386	HDAC4/TGFE	39
0.025386	TGFB2/NOTC	8
0.025386	CDC42/MYO9	8
0.025386	CDK6/HMGA2	8
0.0257689	SUZ12/UBE2	14
0.0257689	EZH2/SUZ12	14
0.0258177	CTNNBIP1/E	83
0.025846	HDAC4/GAPD	25
0.0259076	BCL2L11/BC	18
0.0260458	TGFB3/PTGS	12
0.0260689	APP/BCL2/V	92
0.0261839	COL1A1/EZH	85
0.026246	HDAC4/CTNN	38
0.0262567	BCL2L11/MC	19
0.0262567	CCND1/ESR1	19
0.0262567	BCL2L11/MC	19
0.0263045	EZH2/FOS/S	59
0.0263054	TGFB3/STMN	23
0.0263054	CD276/TGFE	46
0.0263784	ESR1/ARNTL	20
0.0263784	HDAC4/NUPL	20
0.0263784	CTNNBIP1/E	20
0.0263784	CCND1/SPAR	42
0.0263784	PTGS2/MTOR	22
0.0269896	BCL2/VEGFA	28
0.0270838	PDHX/PPT1/	15
0.0270838	PTEN/CDC42	15
0.0270838	PDHX/PPT1/	15
0.027333	SP1/BCL2L1	39
0.0275068	MECP2/STMN	31
0.0276382	CCND1/EZH2	50
0.0282468	BCL2/GNAQ/	11
0.0282468	TGFB1/CDC4	11
0.0282468	ATM/TGFB1/	16
0.0282468	ATM/TGFB1/	16
0.0282468	BCL2/PTEN/	16
0.0282468	BCL2L11/SF	16
0.0283622	PTGS2/TGFE	33
0.0283633	TGFB3/RAB5	13
0.0286699	SP1/VEGFA/	25
0.0287952	EZH2/DNMT3	49
0.0290856	NOP2/RPS10	17
0.0290856	BCL2L11/BC	17
0.0295064	CCND1/APP/	62
0.0295145	APP/CCL4/F	66

0.0295145	VEGFA/PTGS	23
0.0295145	BCL2/IL6/T	23
0.0296111	TGFB3/FOS/	18
0.0298193	VEGFA/PTGS	22
0.0300152	IL6/TBX21/	21
0.0302255	TGFB3/COL3	31
0.0303388	COL5A3/COL	14
0.0303388	ALAD/USP25	14
0.0303388	PTGS2/TGFB	10
0.0305607	HDAC4/BCL2	27
0.0306179	PDHX/PDHA1	6
0.0306179	MFN2/ATG16	6
0.0306179	PDGFRB/PDG	6
0.0306179	VAPA/HSPA8	6
0.0306179	CDC42/MYO9	6
0.0314129	AMOTL1/LAT	12
0.0316767	CD276/TGFB	67
0.0319784	HDAC4/TDG/	37
0.0320457	CLIC4/NR2F	29
0.0320457	MECP2/DNMT	29
0.0321846	CLOCK/PER1	9
0.0321846	RAF1/MAP2K	9
0.0321846	PTEN/SACM1	9
0.032889	FGG/FGB/FG	16
0.032889	FMR1/VAPA/	16
0.0330815	SFPQ/INCEN	31
0.0330815	RAB8B/PEX1	7
0.0330815	PTEN/MTOR/	7
0.0330815	SP1/JUN/TF	7
0.0330815	SOX9/PIM1/	7
0.0330815	CCNT2/PPIL	7
0.0330815	MYCN/ESR1/	7
0.0330815	RAB8B/PEX1	7
0.0330815	RAB8B/PEX1	7
0.0331407	ARNTL/IFNG	38
0.0331407	ARID1A/SMA	8
0.0331407	VEGFA/TGFB	8
0.0331407	PER1/BHLHE	8
0.033314	SP1/BCL2L1	47
0.0335845	TGFB3/ESR1	13
0.0335845	APP/CTR9/R	13
0.0335845	DNMT3B/TGF	13
0.0335845	TGFB1/MTOR	13
0.0335845	ATM/TGFB1/	18
0.0335845	ATXN1/APP/	18
0.0335845	BCL2L11/BC	18
0.033794	CEP68/PAFA	30
0.033794	BCL2L11/BB	30
0.0341728	IL6/TBX21/	11
0.0341728	CCND1/EZH2	11
0.0341728	PTGS2/TGFB	11

0.035263	HDAC4/GAPD	25
0.035558	APP/DYRK1A	14
0.0357521	RAB5A/SH3G	36
0.0358825	DNAJB11/GN	53
0.0358825	PTGS2/TGFB	50
0.0358825	ESR1/FGG/F	31
0.0358825	ITGAV/CD46	31
0.0358825	ITGAV/CD46	31
0.0358825	ITGAV/CD46	31
0.0358825	ITGAV/CD46	31
0.0358977	CEP68/CHEK	28
1.15E-09	EZH2/SP1/D	103
4.13E-08	RAC1/CDC42	106
4.43E-08	RAC1/CDC42	106
7.23E-08	RAC1/CDC42	106
3.14E-07	HDAC4/CCND	35
3.74E-06	CDK6/MECP2	117
6.37E-06	CDK6/CCND1	36
1.26E-05	E2F3/FOS/S	87
3.45E-05	ATXN1/SPAR	36
3.45E-05	EZH2/SUZ12	22
5.64E-05	ATXN1/SPAR	41
5.64E-05	DICER1/AGO	9
5.64E-05	DICER1/AGO	9
5.64E-05	COL7A1/DIO	39
7.22E-05	CDK6/CCND1	30
0.0001403	COL5A3/COL	12
0.0001688	CDK6/CCND1	69
0.0002081	CDK6/CCND1	18
0.0002235	MECP2/DNMT	26
0.0002935	CDC42/SH3G	46
0.0003042	DNMT3A/DNM	81
0.0003042	CDK6/APP/S	88
0.0003988	AP3M1/AP1S	31
0.0003988	AP3M1/AP1S	31
0.0003988	DNMT3A/DNM	51
0.0005278	SFPQ/SRSF1	86
0.000677	APP/ATM/TE	75
0.0008976	RAC1/RAB8B	44
0.0009828	RAC1/CDC42	49
0.0011915	COL7A1/MCF	23
0.0015779	RAC1/CDC42	50
0.0016227	SH3GLB1/CA	28
0.0023032	KLHL28/CDC	70
0.0023032	TGFB3/APP/	22
0.0023032	BACE1/RAB5	95
0.0023032	CCND1/APP/	93
0.0023032	PPP2R2C/PP	18
0.0023032	PPP2R2C/PP	18
0.0023032	TGFB3/APP/	27
0.0024196	COL5A3/COL	33

0.0031263	APP/BACE1/	102
0.0032516	E2F3/FOS/S	46
0.0036006	E2F3/FOS/S	40
0.0036006	KMT5C/INCE	32
0.0040799	SH3GLB1/CA	13
0.0040899	INCENP/DCT	35
0.0040899	APP/BACE1/	43
0.0043799	GAPDH/BCL2	67
0.004707	RAC1/CDC42	41
0.004707	HMGCR/RAB8	19
0.004707	HMGCR/RAB8	19
0.0047292	EZH2/SUZ12	9
0.0053277	FGB/FGA/CD	65
0.0055565	KLHL28/CDC	45
0.0057278	COL4A2/SPA	26
0.0057278	DNMT3A/UBE	7
0.0057278	COL5A3/COL	7
0.0057278	COL5A3/COL	7
0.0059152	DNMT3A/SUZ	11
0.0069242	AP3M1/AP1G	16
0.0078199	INCENP/BOD	28
0.0078199	HDAC4/ELMS	19
0.0079666	APP/RAC1/N	44
0.0085663	RAC1/ARF4/	25
0.0104601	APP/GAPDH/	91
0.010533	PRKAB2/PRK	6
0.0107453	DNMT3A/JUN	12
0.0109563	FGB/FGA/CD	93
0.0110236	FMR1/C9orf	15
0.0110236	CDK6/S100B	39
0.011213	FMR1/AP3M1	20
0.0133955	AP1G1/COPE	17
0.0133955	APP/RAB5A/	59
0.0146327	SHOC2/PPP1	7
0.0146327	FMR1/OIP5/	7
0.016295	EZH2/SUZ12	25
0.016295	APP/COL7A1	24
0.0179725	AP3M1/AP1G	11
0.018927	BOD1/PAFAH	17
0.0189782	MTOR/SH3GL	82
0.0200683	ICK/SPATA2	32
0.0219231	EZH2/EED/T	7
0.0227414	SIRT1/SMAR	11
0.0227962	APP/CDC42/	25
0.0227962	AP3M1/AP1G	15
0.0227962	CCND1/CDK4	33
0.0227962	CANX/GABAR	10
0.0227962	EZH2/SUZ12	20
0.0237933	STAMBP/SPI	16
0.0292556	CCND1/CDK4	29
0.0306942	MORF4L1/BR	7

0.0312527	JUN/HIST1H	9
0.0322559	RAC1/NCKAP	25
0.0347578	STMN1/INCE	79
0.0355858	COL5A3/COL	60
0.0366774	BCL2L11/MC	39
0.037512	MCFD2/AP1G	27
0.0381804	CCND1/CDK4	29
0.0381804	CAB39/PSMD	29
0.0381804	MORF4L1/PH	6
0.0382188	RAC1/RAB8B	19
0.0382188	BACE1/RAB5	66
2.12E-08	MYCN/SP1/M	115
3.00E-08	MYCN/EZH2/	118
4.45E-08	MYCN/EZH2/	120
5.70E-08	HDAC4/E2F3	52
1.21E-06	COL3A1/FOS	32
1.91E-06	E2F3/MYCN/	107
3.65E-06	FOS/TDG/AR	59
3.65E-06	MECP2/HMGA	68
4.98E-06	EZH2/SP1/F	34
5.78E-06	COL1A1/COL	10
6.45E-06	CDC42/KLHL	115
9.59E-06	CTNNBIP1/E	32
1.75E-05	MYCN/SP1/F	75
1.75E-05	CDC42/KLHL	108
2.76E-05	CDC42/BFAR	64
2.87E-05	BCL2/SKI/C	83
8.80E-05	HDAC4/CCND	63
8.81E-05	ARID1A/NCO	80
0.000111	BCL2/SKI/C	78
0.0002114	CDC42/BFAR	60
0.0003297	MECP2/HMGA	42
0.0003984	VEGFA/PTEN	10
0.0004182	HDAC4/SP1/	24
0.0006399	PPP1R14C/P	27
0.0006399	PPP1R14C/P	30
0.0009657	MMP24/MKL2	77
0.0014065	ARNTL/CLOC	16
0.0015126	PIK3CG/PIP	10
0.0017703	TGFB3/COL1	38
0.0021113	BCL2/HMGCR	45
0.0029533	COL3A1/FBN	105
0.0029533	ARID1A/ESR	41
0.0033563	HDAC4/CCND	32
0.0036502	BCL2/HMGCR	34
0.004238	EZH2/MTOR/	34
0.0046373	MSH2/MSH6/	9
0.0046692	FMR1/SECIS	22
0.0054073	TDG/MKL2/H	30
0.0054073	FOS/JUN/ZE	11
0.0055092	SFPQ/SOX9/	31

0.006365	APP/VEGFA/	36
0.0067859	FMR1/KCND3	33
0.0069259	ARID1A/ESR	45
0.0069259	PIK3R1/YWH	9
0.0069259	HDAC4/FOS/	23
0.0069259	PIK3R1/JAK	7
0.0069259	TGFBR1/SMA	7
0.0069259	MSH2/MSH6/	11
0.0069259	DHFR/CPEB1	8
0.0078041	FOS/ARNTL/	21
0.0082127	HDAC4/SP1/	37
0.0082823	EZH2/FOS/F	14
0.0082823	CDK6/CCND1	14
0.0094364	EZH2/SUZ12	30
0.0095913	CHEK1/JAK2	9
0.0095913	SFPQ/SOX9/	27
0.0104048	MAP2K1/ACV	15
0.0104048	TCL1A/TGFE	13
0.0117271	CPEB1/CPEE	7
0.0132619	SOX9/NFE2L	28
0.017949	HDAC4/EZH2	14
0.017949	RAB5A/RAC1	65
0.017949	CDK6/CCND1	13
0.017949	CDK6/CDK4/	12
0.017949	GAPDH/NCOA	12
0.0193997	WEE1/TDG/F	46
0.0193997	FMR1/DDX21	20
0.0193997	MECP2/NCOA	29
0.0269463	SUZ12/NASF	45
0.0277382	ESR1/NFE2L	14
0.0281036	RAB5A/RAC1	77
0.0281036	ARNTL/HSPA	31
0.0281036	EZH2/DNMT3	38
0.0281036	SOX9/TARBF	13
0.0287485	NFE2L2/SUE	12
0.0287485	CCND1/CDK4	12
0.0287485	FMR1/DHFR/	10
0.0287485	RAB5A/RAC1	78
0.031293	RAB5A/RAC1	79
0.0316895	RAB5A/RAC1	77
0.0335119	OTUD4/STAM	6
0.0342046	SP1/JUN/HC	8
0.0358462	SFPQ/NFE2L	21
0.0365019	OTULIN/OTU	22
0.0370713	RAB5A/RAC1	77
0.0373782	ATM/PIK3CG	14
0.038887	PTEN/PPP2R	20
0.038887	ESR1/PIK3C	20
0.03973	ESR1/ATM/F	21
0.0411064	MAP2K1/MAF	7
0.043159	RAF1/TGFBR	39

	ID	Descriptic	GeneRatio	BgRatio	pvalue	p.adjust	qvalue
hsa05210	hsa05210	Colorectal	48/1336	86/8050	9.74E-17	3.08E-14	1.93E-14
hsa04510	hsa04510	Focal adhe	81/1336	201/8050	4.67E-16	7.37E-14	4.62E-14
hsa05225	hsa05225	Hepatocell	68/1336	168/8050	8.79E-14	9.26E-12	5.80E-12
hsa04933	hsa04933	AGE-RAGE s	48/1336	100/8050	2.19E-13	1.73E-11	1.09E-11
hsa01521	hsa01521	EGFR tyros	40/1336	79/8050	2.55E-12	1.61E-10	1.01E-10
hsa04068	hsa04068	FoxO signa	55/1336	131/8050	3.78E-12	1.83E-10	1.15E-10
hsa04151	hsa04151	PI3K-Akt s	110/1336	354/8050	4.06E-12	1.83E-10	1.15E-10
hsa05211	hsa05211	Renal cell	36/1336	69/8050	1.03E-11	4.06E-10	2.54E-10
hsa05205	hsa05205	Proteoglyc	73/1336	205/8050	1.89E-11	6.64E-10	4.16E-10
hsa04218	hsa04218	Cellular s	60/1336	156/8050	3.33E-11	1.05E-09	6.59E-10
hsa05215	hsa05215	Prostate c	43/1336	97/8050	1.05E-10	3.02E-09	1.89E-09
hsa05214	hsa05214	Glioma	36/1336	75/8050	2.26E-10	5.96E-09	3.73E-09
hsa05220	hsa05220	Chronic my	36/1336	76/8050	3.62E-10	8.33E-09	5.22E-09
hsa04140	hsa04140	Autophagy	53/1336	137/8050	3.69E-10	8.33E-09	5.22E-09
hsa05226	hsa05226	Gastric ca	56/1336	149/8050	4.28E-10	9.02E-09	5.65E-09
hsa04152	hsa04152	AMPK signa	48/1336	120/8050	6.67E-10	1.32E-08	8.24E-09
hsa05223	hsa05223	Non-small	33/1336	68/8050	8.93E-10	1.66E-08	1.04E-08
hsa05222	hsa05222	Small cell	40/1336	92/8050	9.64E-10	1.69E-08	1.06E-08
hsa04390	hsa04390	Hippo sigr	57/1336	157/8050	1.38E-09	2.30E-08	1.44E-08
hsa04115	hsa04115	p53 signal	34/1336	73/8050	1.96E-09	3.10E-08	1.94E-08
hsa05224	hsa05224	Breast car	54/1336	147/8050	2.31E-09	3.48E-08	2.18E-08
hsa05218	hsa05218	Melanoma	33/1336	72/8050	5.63E-09	8.09E-08	5.07E-08
hsa05165	hsa05165	Human papi	96/1336	331/8050	6.06E-09	8.32E-08	5.21E-08
hsa04150	hsa04150	mTOR signa	55/1336	155/8050	6.98E-09	9.08E-08	5.68E-08
hsa05212	hsa05212	Pancreatic	34/1336	76/8050	7.18E-09	9.08E-08	5.68E-08
hsa04010	hsa04010	MAPK signa	86/1336	294/8050	2.51E-08	2.94E-07	1.84E-07
hsa04110	hsa04110	Cell cycle	46/1336	124/8050	2.51E-08	2.94E-07	1.84E-07
hsa01522	hsa01522	Endocrine	39/1336	98/8050	3.17E-08	3.44E-07	2.15E-07
hsa05231	hsa05231	Choline me	39/1336	98/8050	3.17E-08	3.44E-07	2.15E-07
hsa04919	hsa04919	Thyroid hc	45/1336	121/8050	3.26E-08	3.44E-07	2.15E-07
hsa04910	hsa04910	Insulin si	49/1336	137/8050	3.47E-08	3.54E-07	2.22E-07
hsa05213	hsa05213	Endometria	27/1336	58/8050	9.22E-08	9.10E-07	5.70E-07
hsa04550	hsa04550	Signaling	49/1336	143/8050	1.62E-07	1.56E-06	9.74E-07
hsa05161	hsa05161	Hepatitis	53/1336	162/8050	2.83E-07	2.58E-06	1.62E-06
hsa04066	hsa04066	HIF-1 sigr	40/1336	109/8050	2.86E-07	2.58E-06	1.62E-06
hsa04810	hsa04810	Regulatior	64/1336	214/8050	6.76E-07	5.84E-06	3.65E-06
hsa05206	hsa05206	MicroRNAs	85/1336	310/8050	6.83E-07	5.84E-06	3.65E-06
hsa04015	hsa04015	Rap1 signa	63/1336	210/8050	7.33E-07	6.09E-06	3.82E-06
hsa04926	hsa04926	Relaxin si	44/1336	129/8050	8.00E-07	6.48E-06	4.06E-06
hsa05418	hsa05418	Fluid shea	46/1336	139/8050	1.20E-06	9.46E-06	5.93E-06
hsa04722	hsa04722	Neurotroph	41/1336	119/8050	1.41E-06	1.08E-05	6.78E-06
hsa04014	hsa04014	Ras signal	67/1336	232/8050	1.49E-06	1.12E-05	7.04E-06
hsa05167	hsa05167	Kaposi sar	57/1336	189/8050	2.05E-06	1.51E-05	9.44E-06
hsa04012	hsa04012	ErbB signa	32/1336	85/8050	2.31E-06	1.66E-05	1.04E-05
hsa05230	hsa05230	Central ca	28/1336	70/8050	2.45E-06	1.72E-05	1.08E-05
hsa05163	hsa05163	Human cytc	64/1336	225/8050	4.48E-06	3.08E-05	1.93E-05
hsa04371	hsa04371	Apelin sig	44/1336	137/8050	4.99E-06	3.30E-05	2.06E-05
hsa05170	hsa05170	Human immu	61/1336	212/8050	5.01E-06	3.30E-05	2.06E-05
hsa05216	hsa05216	Thyroid ca	18/1336	37/8050	5.99E-06	3.87E-05	2.42E-05
hsa04070	hsa04070	Phosphatic	34/1336	97/8050	7.18E-06	4.53E-05	2.84E-05

hsa05131	hsa05131	Shigellosi	67/1336	242/8050	7.37E-06	4.57E-05	2.86E-05
hsa05202	hsa05202	Transcript	56/1336	192/8050	7.80E-06	4.74E-05	2.97E-05
hsa05221	hsa05221	Acute myel	26/1336	67/8050	1.07E-05	6.39E-05	4.00E-05
hsa04520	hsa04520	Adherens j	27/1336	71/8050	1.13E-05	6.63E-05	4.15E-05
hsa05162	hsa05162	Measles	43/1336	139/8050	1.82E-05	0.0001048	6.57E-05
hsa04211	hsa04211	Longevity	31/1336	89/8050	2.08E-05	0.0001174	7.35E-05
hsa04210	hsa04210	Apoptosis	42/1336	136/8050	2.38E-05	0.0001321	8.27E-05
hsa04213	hsa04213	Longevity	24/1336	62/8050	2.44E-05	0.0001331	8.34E-05
hsa04071	hsa04071	Sphingolip	38/1336	119/8050	2.51E-05	0.0001332	8.34E-05
hsa05132	hsa05132	Salmonella	59/1336	213/8050	2.53E-05	0.0001332	8.34E-05
hsa04917	hsa04917	Prolactin	26/1336	70/8050	2.67E-05	0.0001382	8.65E-05
hsa05219	hsa05219	Bladder ca	18/1336	41/8050	3.51E-05	0.0001791	0.0001121
hsa05166	hsa05166	Human T-ce	59/1336	219/8050	6.09E-05	0.000301	0.0001885
hsa04935	hsa04935	Growth hor	37/1336	119/8050	6.10E-05	0.000301	0.0001885
hsa04931	hsa04931	Insulin re	34/1336	108/8050	9.09E-05	0.0004421	0.0002769
hsa04660	hsa04660	T cell rec	33/1336	104/8050	9.68E-05	0.0004633	0.0002901
hsa04370	hsa04370	VEGF signa	22/1336	59/8050	0.0001024	0.0004827	0.0003023
hsa04664	hsa04664	Fc epsilon	24/1336	68/8050	0.0001378	0.0006403	0.000401
hsa04912	hsa04912	GnRH signa	30/1336	93/8050	0.0001422	0.0006514	0.0004079
hsa05235	hsa05235	PD-L1 expr	29/1336	89/8050	0.0001498	0.0006762	0.0004235
hsa04310	hsa04310	Wnt signal	45/1336	160/8050	0.0001535	0.0006834	0.000428
hsa04512	hsa04512	ECM-recept	28/1336	88/8050	0.0003033	0.0013313	0.0008337
hsa04611	hsa04611	Platelet a	36/1336	124/8050	0.0003499	0.0014943	0.0009358
hsa04666	hsa04666	Fc gamma R	29/1336	93/8050	0.0003499	0.0014943	0.0009358
hsa05203	hsa05203	Viral carc	53/1336	204/8050	0.0003799	0.0016006	0.0010024
hsa05135	hsa05135	Yersinia i	37/1336	130/8050	0.0004467	0.0018574	0.0011632
hsa00562	hsa00562	Inositol p	24/1336	73/8050	0.0004683	0.0018731	0.001173
hsa01524	hsa01524	Platinum d	24/1336	73/8050	0.0004683	0.0018731	0.001173
hsa05100	hsa05100	Bacterial	24/1336	73/8050	0.0004683	0.0018731	0.001173
hsa04625	hsa04625	C-type lec	31/1336	104/8050	0.0005357	0.0021161	0.0013252
hsa04934	hsa04934	Cushing sy	42/1336	155/8050	0.0005929	0.002313	0.0014485
hsa04915	hsa04915	Estrogen s	38/1336	138/8050	0.0007607	0.0029314	0.0018358
hsa05169	hsa05169	Epstein-Ba	51/1336	202/8050	0.0009853	0.0037168	0.0023277
hsa04350	hsa04350	TGF-beta s	28/1336	94/8050	0.000988	0.0037168	0.0023277
hsa04921	hsa04921	Oxytocin s	41/1336	154/8050	0.0010024	0.0037266	0.0023338
hsa04728	hsa04728	Dopaminerg	36/1336	132/8050	0.0012493	0.0045906	0.0028748
hsa04141	hsa04141	Protein pr	43/1336	167/8050	0.0015781	0.0057321	0.0035897
hsa04922	hsa04922	Glucagon s	30/1336	106/8050	0.0016464	0.005912	0.0037024
hsa04540	hsa04540	Gap juncti	26/1336	88/8050	0.00168	0.0059648	0.0037355
hsa04144	hsa04144	Endocytosi	59/1336	248/8050	0.0019567	0.0068702	0.0043024
hsa05145	hsa05145	Toxoplasmo	31/1336	112/8050	0.0020502	0.0071195	0.0044586
hsa05168	hsa05168	Herpes sim	105/1336	490/8050	0.0023708	0.008143	0.0050996
hsa05160	hsa05160	Hepatitis	40/1336	157/8050	0.0027768	0.0094213	0.0059001
hsa05017	hsa05017	Spinocereb	35/1336	133/8050	0.0028025	0.0094213	0.0059001
hsa04662	hsa04662	B cell rec	24/1336	82/8050	0.0028557	0.0094989	0.0059486
hsa04916	hsa04916	Melanogene	28/1336	101/8050	0.0032144	0.0105807	0.0066262
hsa05146	hsa05146	Amoebiasis	28/1336	102/8050	0.0037463	0.0122043	0.0076429
hsa04668	hsa04668	TNF signal	30/1336	112/8050	0.0040969	0.0132106	0.0082731
hsa04530	hsa04530	Tight junc	40/1336	162/8050	0.0050136	0.0158431	0.0099217
hsa04630	hsa04630	JAK-STAT s	40/1336	162/8050	0.0050136	0.0158431	0.0099217
hsa04730	hsa04730	Long-term	18/1336	60/8050	0.0069176	0.0215086	0.0134697

hsa04920	hsa04920	Adipocytok	20/1336	69/8050	0.0069426	0.0215086	0.0134697
hsa05142	hsa05142	Chagas dis	27/1336	102/8050	0.0074154	0.0227502	0.0142473
hsa04710	hsa04710	Circadian	11/1336	31/8050	0.0085044	0.0258403	0.0161825
hsa04914	hsa04914	Progesterc	26/1336	99/8050	0.0094582	0.0284646	0.0178259
hsa04072	hsa04072	Phospholip	36/1336	148/8050	0.0095929	0.0285976	0.0179093
hsa04750	hsa04750	Inflammatc	26/1336	100/8050	0.0108205	0.0319558	0.0200123
hsa04928	hsa04928	Parathyroi	27/1336	106/8050	0.0125798	0.0366712	0.0229653
hsa04137	hsa04137	Mitophagy	19/1336	68/8050	0.0126493	0.0366712	0.0229653
hsa04659	hsa04659	Th17 cell	27/1336	107/8050	0.0142444	0.0409201	0.0256262
hsa04392	hsa04392	Hippo sigr	10/1336	29/8050	0.0147885	0.0421006	0.0263655
hsa04725	hsa04725	Cholinergi	28/1336	113/8050	0.0162282	0.0457867	0.0286739
hsa04114	hsa04114	Oocyte mei	31/1336	128/8050	0.0164226	0.0459251	0.0287605

geneID	Count
7043/595/1	48
1284/1277/	81
7043/1871/	68
7043/1284/	48
10018/596/	40
7043/595/1	55
1284/1277/	110
7043/7422/	36
1277/595/1	73
7043/1871/	60
1871/595/5	43
1871/1021/	36
7043/1871/	36
596/5894/5	53
7043/1871/	56
595/2475/5	48
1871/1021/	33
1284/1871/	40
7043/595/2	57
1021/595/5	34
1871/1021/	54
1871/1021/	33
1284/1277/	96
5894/5728/	55
7043/1871/	34
7043/2353/	86
7043/7465/	46
1871/595/6	39
6667/2353/	39
595/2099/8	45
5894/2475/	49
595/5894/5	27
5894/54361	49
7043/1871/	53
2597/596/7	40
5894/5879/	64
9759/1871/	85
7422/5894/	63
1284/1277/	44
2353/596/7	46
596/5894/5	41
7422/5894/	67
1871/1021/	57
5894/2475/	32
5894/5728/	28
1871/1021/	64
9759/595/5	44
7465/2353/	61
595/5604/1	18
5728/5295/	34

596/472/58	67
4613/6667/	56
595/5894/2	26
5879/998/7	27
1021/595/2	43
2475/10000	31
10018/2353	42
2475/10000	24
596/5894/5	38
2353/2597/	59
595/2353/2	26
1871/595/7	18
7043/1871/	59
2353/5894/	37
5728/2475/	34
2353/5894/	33
7422/5743/	22
5894/5879/	24
4313/5894/	30
2353/5894/	29
56998/595/	45
1284/1277/	28
1277/1281/	36
5894/5879/	29
9759/1021/	53
2353/5879/	37
5728/5294/	24
596/472/27	24
5879/998/5	24
5743/5894/	31
1871/1021/	42
6667/2353/	38
1871/1021/	51
7043/6667/	28
595/2353/5	41
2353/406/2	36
51726/596/	43
10000/5565	30
5894/79861	26
5868/998/5	59
7043/3915/	31
596/2475/3	105
1871/1021/	40
6310/6667/	35
2353/5894/	24
5894/54361	28
7043/1284/	28
2353/5743/	30
595/1019/5	40
595/4170/5	40
5894/1392/	18

2475/1147/	20
7043/2353/	27
406/5565/9	11
5894/10000	26
5894/2475/	36
2776/5295/	26
6667/2353/	27
6667/3725/	19
2353/7040/	27
122786/977	10
2353/596/5	28
898/8697/5	31
