# Supplementary material for: A Four-miRNA-Based Diagnostic Signature for Rheumatoid Arthritis
Source: Dis Markers. 2022 Feb 22;2022:6693589. doi: 10.1155/2022/6693589 (PMC8889404; doi:10.1155/2022/6693589)
Supplement: Supplementary 3 — Table S3: the correlation analysis results of four key miRNAs. [file 6693589.f3.pdf]

Table S2 Correlation analysis resullt

	hsa-miR-1	hsa-miR-1	hsa-miR-1	hsa-miR-99b-5p
hsa-miR-1	1	0.423709	0.752385	-0.19644
hsa-miR-1	0.423709	1	0.465549	-0.38759
hsa-miR-1	0.752385	0.465549	1	-0.2127
hsa-miR-9	-0.19644	-0.38759	-0.2127	1
