# Supplementary figures and images for: A Four-miRNA-Based Diagnostic Signature for Rheumatoid Arthritis
Source: Dis Markers. 2022 Feb 22;2022:6693589. doi: 10.1155/2022/6693589 (PMC8889404; doi:10.1155/2022/6693589)

A

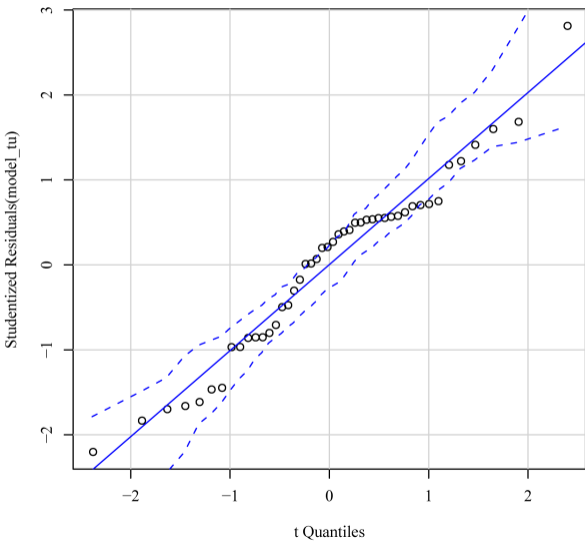

B

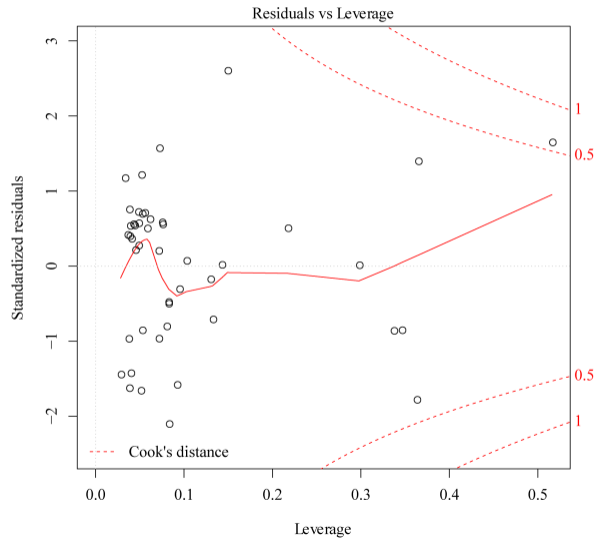

Supplement: Supplementary 4 — Fig. S1: the diagnosis diagram of the logistic regression model. (A) The normal Q-Q graph. The points on the graph should fall on a line at an angle of 45 degrees. If the deviation is too large, then the model violates the normal assumption. (B) The diagram of residuals vs. leverage. The red dotted line indicates the COOK distance. Generally, a point with the COOK greater than 0.5 is a very “influential” point, which affects the reliability of the model. [file 6693589.f4.pdf]
